# Systematic synthetic study of four diastereomerically distinct limonene-1,2-diols and their corresponding cyclic carbonates

**DOI:** 10.3762/bjoc.15.13

**Published:** 2019-01-14

**Authors:** Hiroshi Morikawa, Jun-ichi Yamaguchi, Shun-ichi Sugimura, Masato Minamoto, Yuuta Gorou, Hisatoyo Morinaga, Suguru Motokucho

**Affiliations:** 1 Department of Applied Chemistry, Kanagawa Institute of Technology, 1030, Shimo-ogino, Atsugi, Kanagawa 243-0292, Japan; 2 Faculty of Education, Graduate Faculty of Interdisciplinary Research, University of Yamanashi, 4-4-37, Takeda, Kofu, Yamanashi 400-8510, Japan; 3 Graduate School of Engineering, Nagasaki University, 1-14, Bunkyo-machi, Nagasaki-city 852-8521, Japan

**Keywords:** cyclic carbonate, diastereomer, diol, limonene, NMR

## Abstract

In order to produce versatile and potentially functional terpene-based compounds, a (*R*)-limonene-derived diol and its corresponding five-membered cyclic carbonate were prepared. The diol (cyclic carbonate) comprises four diastereomers based on the stereochemical configuration of the diol (and cyclic carbonate) moiety. By choosing the appropriate starting compounds (*trans*- and *cis*-limonene oxide) and conditions, the desired diastereomers were synthesised in moderate to high yields with, in most cases, high stereoselectivity. Comparison of the NMR data of the obtained diols and carbonates revealed that the four different diastereomers of each compound could be distinguished by reference to their characteristic signals.

## Introduction

(*R*)-Limonene (LM) is a naturally occurring terpene, and therefore a very attractive and renewable resource [1]. Its derivatives have versatilely and widely been studied [1–4].

**Figure 1:**
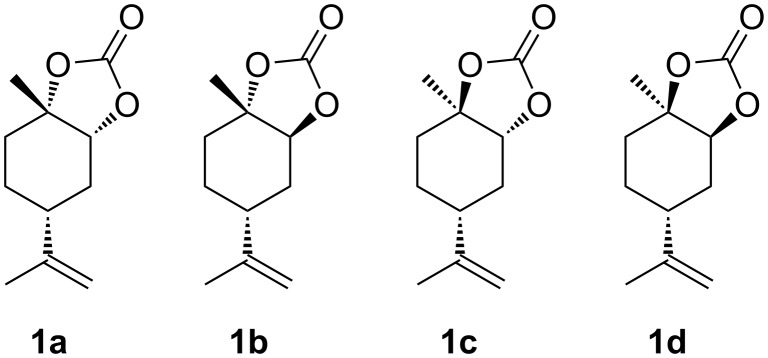
Diastereomers of LM5CCs, **1a**–**d**.

**Figure 2:**
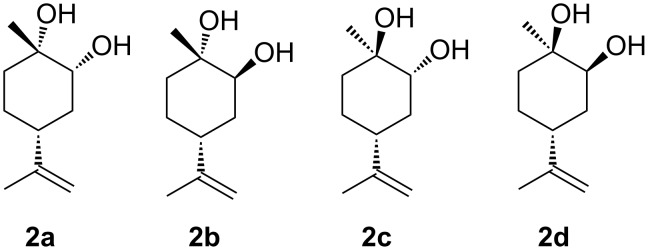
Diastereomers of LMdiols, **2a**–**d**.

Otherwise, syntheses of five-membered cyclic carbonates (5CCs) have been intensively investigated [5–7] in terms of utilisation of CO_2_ and the further reactions to produce functional chemicals such as oxazolizin-2-ones [8] and polyurethanes [9]. For (*R*)-limonene-derived 5CC (LM5CC), four diastereomers are considered from the different stereochemical configurations at the 1- and 2-positions of LM (Figure 1). A few studies on **1a** and **1d** have been recently reported [10–11], whereas **1b** and **1c** have never been synthesised. Consequently, the systematic and stereoselective syntheses of these four LM5CCs are of deep significance, largely because LM derivatives with their versatile functionality can be effectively utilised in organic and polymer chemistry.

(4*R*)-Limonene-1,2-diols (LMdiols) are some of the most important LM derivatives because they act as precursors of bioactive molecules [12–14]. Furthermore, LMdiol is detected as metabolite in vivo in biochemistry [15], and is known to react in the atmosphere to afford the secondary organic aerosol as air pollutants [16]. Among the four diastereomers of LMdiols (Figure 2), **2b** and **2c** have already been reported [14,17–20]. Conversely, the distinct characterisations of **2a** and **2d** remain unreported.

**Scheme 1:**
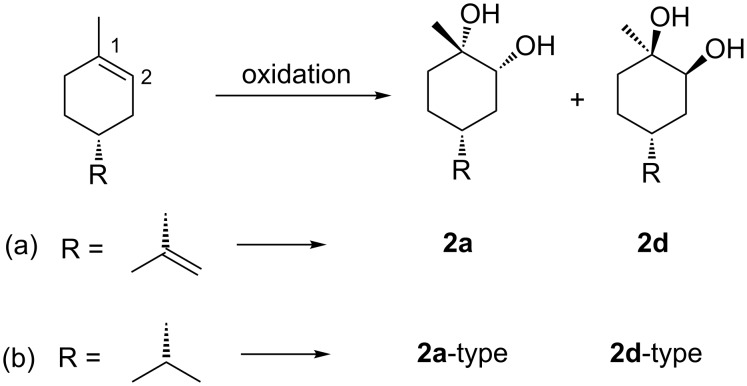
Dihydroxylation of (a) (*R*)-limonene and (b) (*R*)-dihydrolimonene.

Very recently, Wang et al. synthesised **2a** and **2d** using OsO_4,_ and exclusively **2d** using Sharpless AD-mix-β [21] as a catalyst (Scheme 1a) [16]. In contrast to that, Mori reported that (*R*)-dihydrolimonene, the structure of which is similar to LM, was oxidised using Sharpless AD-mix-β to afford a **2a**-type main product (Scheme 1b) [22]. Hao et al. also reported the synthesis of **2a** and **2d** using OsO_4_ (Scheme 1a) [23]. However, the NMR data of **2a** and **2d** were not consistent with those [16] reported by Wang. Moreover, they referred to a published paper [24] for the assignment of **2d**. However, the reaction mechanism in the referred paper indicates the formation of **2b**. A rational explanation for the syntheses of **2a** and **2d** is not available at the present stage.

Since Royals et al. presented a pioneering study despite insufficient data for three diastereomers of LMdiol in 1966 [25], no further reports on the systematic synthesis and comparison of the four LMdiols have been published. The stereochemical structures of LMdiols are not depicted in several reports [26] presumably due to a lack of reliable characterisation data for the four LMdiols. Therefore, reliable characterisation data of LMdiols and LM5CCs are required, particularly if the confusion surrounding LMdiols should be resolved.

Herein, we focus on two compounds, LMdiol and LM5CC, as versatile and functional LM derivatives, and report the systematic synthesis of each of their four diastereomers and their characterisation using spectroscopic methods, mainly NMR. Comparing the data, unambiguous distinction of the four diastereomers is achieved in both cases.

## Results and Discussion

### Syntheses of LM5CC **1a** and **1d** and LMdiol **2a** and **2d**

Facile syntheses of two LM5CCs **1a** and **1d** and one LMdiol **2d** have been preliminarily reported in our previous study [27]. Of these, **1a** has already been synthesised by other groups [10–11] as well as our group. Particularly, Kleij et al. revealed the solid-state structure of **1a** via X-ray analysis [11], but the isolation and characterisation of **1d** has only been reported by our group.

Herein, we report the synthesis of **1a** from the *trans*-isomer of (*R*)-limonene oxide (LO) and **1d** from *cis*-LO using CO_2_ and tetrabutylammonium chloride (TBAC) as a catalyst. Both reactions proceeded without any side reactions and produced no unwanted isomers, such as **1c**. Though the CO_2_ pressure employed in our previous study was 3 MPa [27], 5 MPa CO_2_ was applied in the present study to promote a higher consumption of LO. The result showed the isolated yields were improved to 84% yield for **1a** and 30% yield for **1d**.

**Scheme 2:**
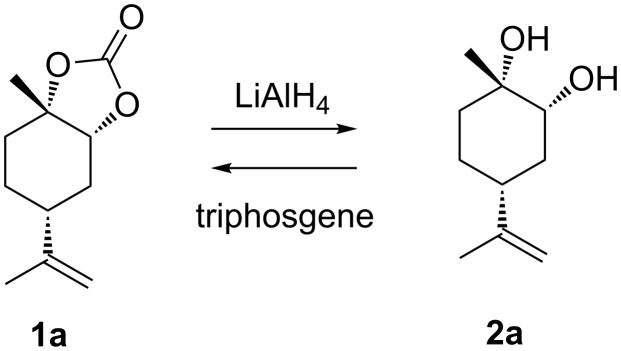
Reduction of LM5CC (**1a**) and carbonation of LMdiol (**2a**).

**Scheme 3:**
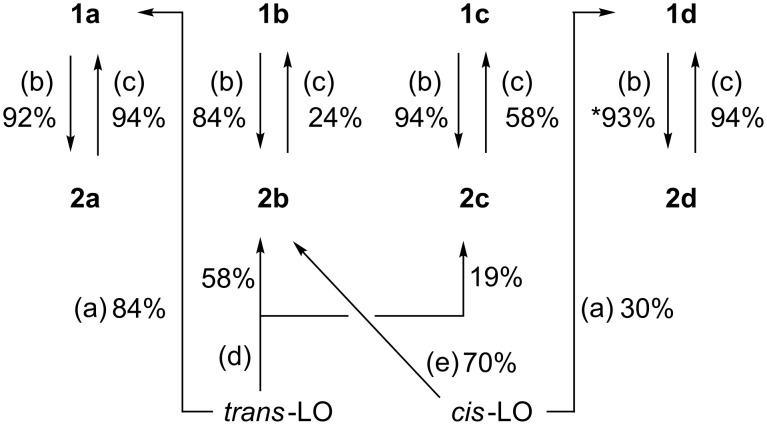
Overall synthetic routes to LM5CCs and LMdiols. The 93% value (standing for *) from **1d** to **2d** was cited from ref [27]. Reagents and conditions: (a) 5 MPa CO_2_, 10 mol % TBAC, 100 °C, 72 h; (b) LAH, rt, 2 h; (c) triphosgene, rt, 2 h; (d) H_2_O/dioxane (1:1, v/v), 120 °C, 72 h; (e) H_2_O, 90 °C, 12 h.

In our previous report, **1d** was reduced by lithium aluminium hydride (LAH) to obtain the corresponding LMdiol **2d** [27]. Notably, the structure of **2d** was confirmed by X-ray analysis. It has been reported that reduction of the 5CC moiety with LAH gives the corresponding diol with the same stereochemical configuration at the carbon atoms as of the original 5CC moiety [28–29]. Such a reduction with LAH was initially applied in this study to **1a** (Scheme 2) as an analogy to the reaction of **1d**. The reaction proceeded smoothly giving the corresponding LMdiol **2a** in an excellent yield (Scheme 3).

It is also possible to synthesise 5CCs from diols by reaction with triphosgene (Scheme 2). It is well known that this carbonation reaction maintains the stereochemical configuration of the original diol [30–31]. Accordingly, the reactions of **2a** and **2d** with triphosgene successfully afforded **1a** and **1d**, respectively, in this study (Scheme 3). The two carbonates obtained from the diols and triphosgene were spectroscopically identical to those synthesised from LO and CO_2_, as confirmed by ^1^H and ^13^C NMR analyses.

### Syntheses of LM5CC **1b** and **1c** and LMdiol **2b** and **2c**

The reaction of *trans*-LO and water in dioxane afforded a mixture of two LMdiols **2b**/**2c**. After purification, **2b** and **2c** were isolated in 58% and 19% yields, respectively (Scheme 3). Conversely, the reaction of *cis*-LO with water afforded only **2b**, which was obtained in 70% yield after purification. These results are in good agreement with those of previous reports [18,20,32].

Similarly to the aforementioned carbonation of **2a** (Scheme 2), **2b** and **2c** were also reacted with triphosgene. Neither of the desired products **1b** and **1c** have been synthesised until now. When **2c** was used, the carbonation proceeded smoothly to afford the corresponding carbonate **1c** exclusively. The isolated yield after purification was moderate (58%). Conversely, the reaction of **2b** was accompanied by side reactions. Although the starting material **2b** was completely consumed, two major products were detected by thin-layer chromatography. After purification, the desired **1b** was obtained in a relatively low yield of 24%.

**Scheme 4:**
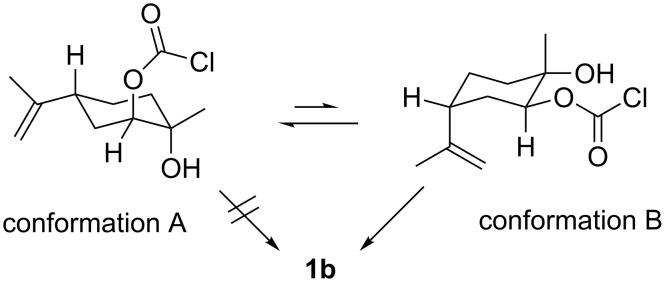
Plausible conformations of the intermediate in the reaction of LMdiol (**2b**) with triphosgene.

Two conformations (A and B in Scheme 4) are possible for the intermediate when **2b** is reacted with triphosgene. The conformation A is expected to be more stable because the isopropenyl group remains in the equatorial position on the cyclohexane ring. In this conformation, the remaining hydroxy group is located in the axial position and away from the carbonyl group of the chloroformate moiety. This conformation makes the formation of the five-membered cyclic structure by ring-closing very difficult, leading to the low yield. However, although the yield of **1b** was low, both **1b** and **1c** were successfully synthesised for the first time in the present study.

The carbonates **1b** and **1c** were also reacted with LAH in the same manner as **1a** (Scheme 2). After reaction and purification, **2b** and **2c** were afforded in high yields (Scheme 3). The two resultant compounds were characterised by ^1^H and ^13^C NMR analyses, and the spectroscopic data were identical to those of **2b** and **2c** obtained from LO and water. Therefore, these results confirm the formation of the two carbonates **1b** and **1c**.

### Overview of synthetic routes to the LM5CCs and LMdiols

The overall results for the syntheses of the four LM5CCs and the four LMdiols are shown in Scheme 3. All the reactions do not lead to the formation of other diastereomers as byproducts except for the reaction of *trans*-LO with water (Scheme 3, conditions (d)). The reduction under conditions (b) and carbonation under conditions (a) and (c) retain the diastereomeric conformations around the epoxy, 5CC and diol moieties.

In all the reactions, the yields for the reduction of LM5CC with LAH were high at 84–94%. Conversely, the yields of the carbonations to LM5CC differ depending on both the starting material and reaction conditions, i.e., (a) and (c) in Scheme 3. Thus, all the LM5CCs and LMdiols can be synthesised by choosing the appropriate starting LO and reaction conditions with high diastereomeric stereoselectivity and, in most cases, moderate to high yields.

To compare the four LMdiols and the four LM5CCs, a set of the characteristic analytical data such as melting points, optical rotation values and Kovats retention index values in gas chromatography was listed in Tables S1 and S2 in Supporting Information File 1.

### Analysis and comparison of the four LM5CCs

In our previous study, the assignments of the NMR signals for **1a** and **1d** were not adequate [27]. Table 1 lists the ^13^C NMR chemical shift values for all LM5CCs, showing the assignments for each of the carbon atoms. The multiplicities were ascertained using DEPT135 analysis (Supporting Information File 1, pages S13, S18, S23, and S28).

**Table 1:** Chemical shift values (ppm) of four LM5CCs with the assignments^a^.

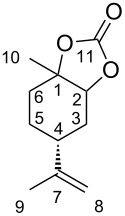							

**1a**		**1b**		**1c**		**1d**	

154.6	(C11)	155.1	(C11)	155.2	(C11)	154.4	(C11)
147.2	(C7)	145.7	(C7)	146.4	(C7)	147.3	(C7)
110.0	(C8)	111.9	(C8)	110.7	(C8)	109.8	(C8)
82.0	(C1)	86.2	(C1)	85.4	(C1)	82.6	(C1)
80.4	(C2)	81.6	(C2)	84.8	(C2)	81.7	(C2)
39.8	(C4)	38.5	(C4)	43.0	(C4)	37.3	(C4)
33.9	(C3)	31.7	(C6)	33.0	(C6)	34.1	(C6)
32.9	(C6)	25.8	(C3)	28.9	(C3)	30.5	(C3)
26.1	(C10)	25.1	(C5)	27.9	(C5)	26.2	(C5)
25.6	(C5)	22.5	(C9)	20.8	(C9)	22.2	(C10)
20.5	(C9)	16.8	(C10)	16.9	(C10)	20.8	(C9)

^a^In CDCl_3_.

As characteristic signals, the C9 values are observed at higher magnetic fields than the C10 values for **1a** and **1d**. However, the relationship is reversed for **1b** and **1c**. In addition, the values for C10 in **1b** and **1c** are observed at the higher magnetic fields (16.8 and 16.9 ppm, respectively) compared with those for **1a** and **1d**. The order of C3, C6 and C5 signals for **1a** is also different from that (C6, C3, C5) of the other three LM5CCs.

**Figure 3:**
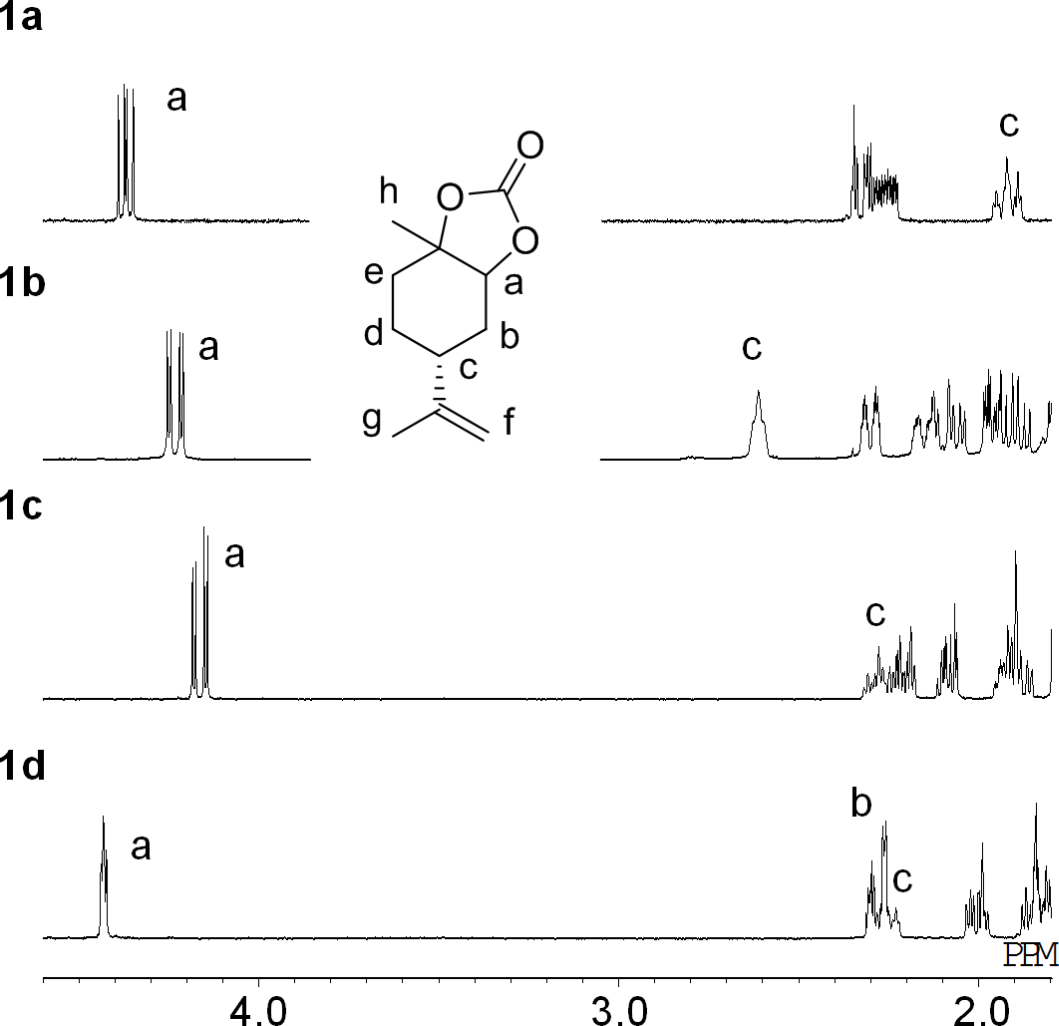
^1^H NMR spectra of LM5CCs **1a**–**d** in CDCl_3_.

^1^H NMR spectra of the four LM5CCs are shown in Figure 3 (selected range) and Supporting Information File 1, page S32 (full range) with the assignments of signals a–h. Three characteristic signals f, a and c are clearly observed at 5.0–4.7, 4.4–4.2 and 2.4–1.9 ppm, respectively. Obviously, the respective diastereomers can be distinguished by comparison of the characteristic signals. The determination of all the signals in ^1^H and ^13^C NMR is also supported by ^1^H,^1^H COSY and HETCOR (Supporting Information File 1).

### Analysis and comparison for the four LMdiols

Table 2 lists the chemical shifts in the ^13^C NMR spectra with the assignments for all LMdiols. The listed values for **2a** and **2c** are close, but distinguishable. Conversely, **2b** and **2d** present rather similar values for all the signals. In the 28–24 ppm region, two characteristic signals ascribed to C5 and C10, which are clearly distinguishable by DEPT 135, are observed. The two values are approximately 4 ppm apart for **2d**, but less than 1 ppm apart for **2b**. Furthermore, the order is reversed for the two samples; the signal of C10 appears at higher field than that for C5 in case of **2d**. This demonstrates that **2b** and **2d** are similar but can be differentiated by their characteristic NMR signals.

**Table 2:** Chemical shift values (ppm) of four LMdiols with the assignments^a^.

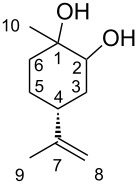							

**2a**		**2b**		**2c**		**2d**	

149.0	(C7)	149.1	(C7)	148.4	(C7)	149.0	(C7)
108.8	(C8)	109.0	(C8)	109.1	(C8)	108.9	(C8)
75.1	(C2)	73.8	(C2)	77.2	(C2)	73.7	(C2)
70.8	(C1)	71.4	(C1)	74.0	(C1)	71.9	(C1)
43.7	(C4)	37.4	(C4)	43.6	(C4)	37.5	(C4)
37.3	(C6)	33.9	(C3)	38.5	(C6)	34.5	(C3)
35.5	(C3)	33.7	(C6)	36.1	(C3)	34.1	(C6)
27.1	(C10)	26.5	(C10)	28.7	(C5)	28.2	(C5)
26.1	(C5)	26.1	(C5)	20.9	(C9)	24.4	(C10)
20.8	(C9)	21.1	(C9)	18.9	(C10)	21.2	(C9)

^a^In CDCl_3_.

**Figure 4:**
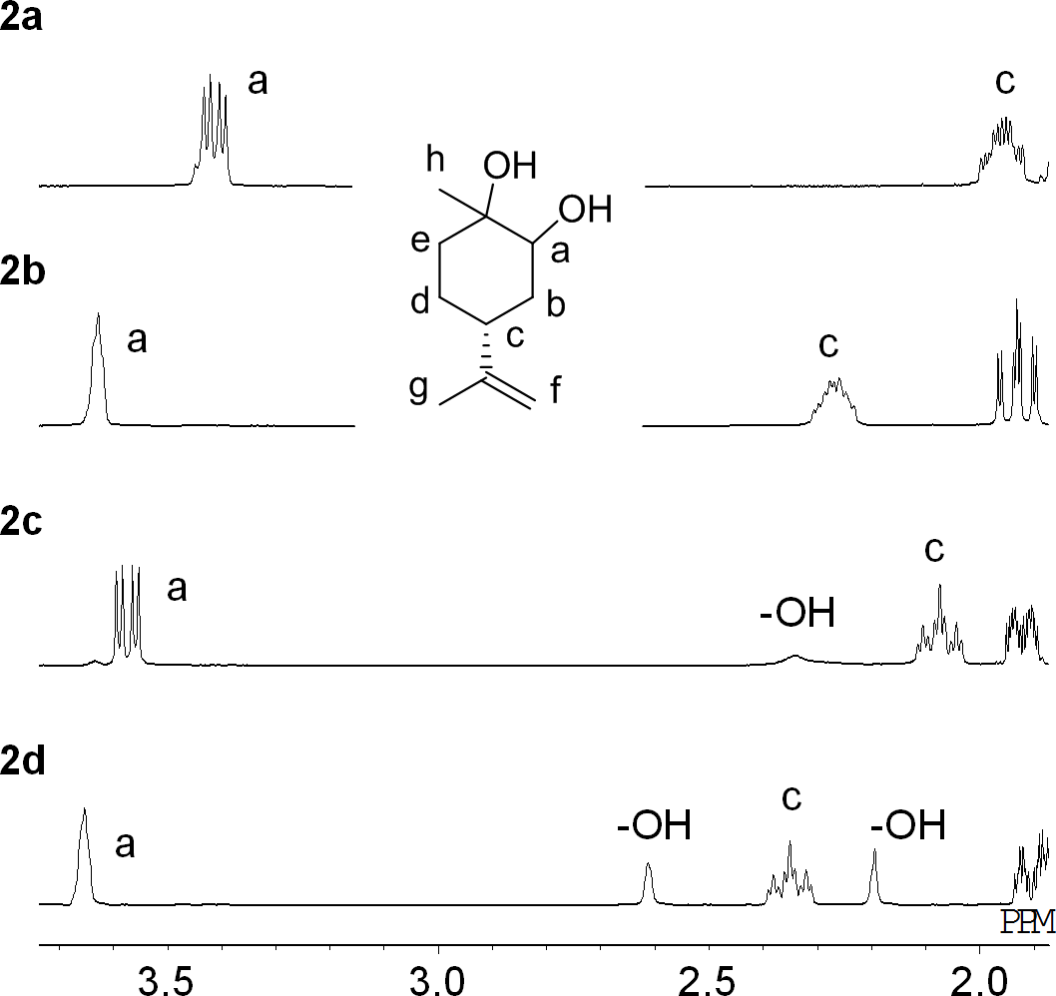
^1^H NMR spectra of LMdiols **2a**–**d** in CDCl_3_.

In ^1^H NMR spectra of the four LMdiols (Figure 4 and Supporting Information File 1, page S61), characteristic signals a are observed at 3.41 for **2a** and 3.58 ppm for **2c**, demonstrating that they are different. However, signals a and c as well as others show similar values for **2b** and **2d**. The most significant difference is observed for the signal at 1.3 ppm of **2d** (Supporting Information File 1, page S55), allowing the two samples to be distinguished.

The assignments in the ^1^H and ^13^C NMR signals were supported by DEPT135, ^1^H,^1^H COSY, HETCOR and HMBC (heteronuclear multiple bond correlation) analyses. Two samples of **2a** and **2b** were also subjected to 1,1-ADEQUATE (adequate double quantum transfer experiment) analysis.

### Structures of four LMdiols and four LM5CCs

**Figure 5:**
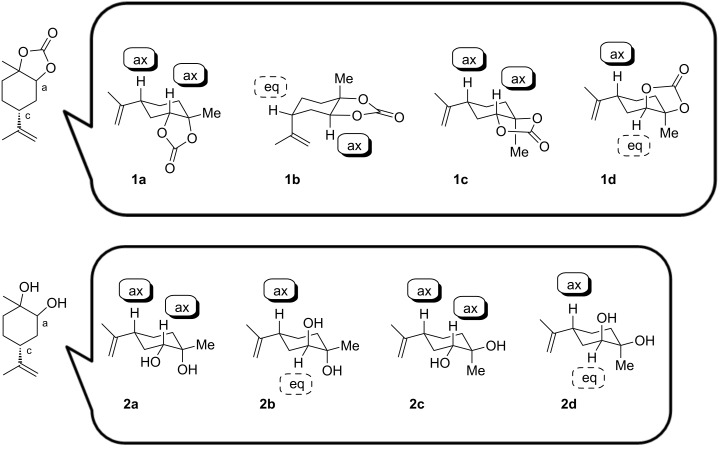
Plausible conformations of LM5CCs and LMdiols in CDCl_3_.

Plausible conformations for the LM5CCs and LMdiols in CDCl_3_ are proposed in Figure 5. In Figure 3, large coupling constants (*J* > ca. 10 Hz) for the signal a are observed for **1a**–**c**. Conversely, the signal for **1d** exhibits a small coupling constant (*J* < ca. 3 Hz), showing a largely singlet shape. For the former three LM5CCs, this means that the proton a locates at the axial position on the cyclohexane ring and interacts strongly with the neighbouring proton b. Conversely, for **1d**, the proton locates at the equatorial position; thus, the interaction with proton b is weak. These interactions support the structures shown in Figure 5. For **1b**, two oxygen atoms located at the equatorial positions form a 5CC cyclic structure; then, the isopropenyl group is located at the axial position. This speculation is also supported by the low 24% yield in the synthesis of **1b** from **2b**, as shown in Scheme 3 and Scheme 4.

In these conformations, proton c is expected to be located in the axial position for **1a**, **1c** and **1d**, and in the equatorial position for **1b**. In fact, the coupling constants match well the conformations in Figure 5 although the signal c for **1d** unfortunately overlaps with other signals. The structure of **1a** is in good agreement with the solid-state structure reported previously [11].

Conformations of the four LMdiols were also considered in the same manner as those of LM5CCs by reference to the a and c signals in Figure 4. The coupling constants for the signal a support the structures shown in Figure 5. Indeed, the equatorial position of the isopropenyl group for **2c** and **2d** in the solid state was confirmed by X-ray diffraction analysis, as reported previously [20,27]. The c protons are expected to stay in the axial positions for all the LMdiols. These considerations are consistent with the fact that the signals exhibit large coupling constants for **2a**, **2c** and **2d**. For **2b** in CDCl_3_, the coupling constants could not be estimated because of the multiplet shape; however, a large coupling constant was clearly observed in CD_3_OD and in benzene-*d*_6_ (Supporting Information File 1, page S42).

## Conclusion

Four diastereomers of (*R*)-limonene-derived diols (LMdiols) and the corresponding five-membered cyclic carbonates (LM5CCs) were synthesised and characterised by NMR analysis. In particular, the NMR data for **2b** and **2d** exhibit rather similar values. The assignments of ^1^H and ^13^C NMR signals were found to provide a means of differentiating the respective diastereomers for the first time. These fundamental studies will contribute to the organic, bioorganic and environmental chemistry of (*R*)-limonene-based compounds as well as promoting their potential application.

## Supporting Information

File 1 Experimental, synthesis, and NMR and FTIR spectra of all the compounds.
