# Supplementary material for: Systematic synthetic study of four diastereomerically distinct limonene-1,2-diols and their corresponding cyclic carbonates
Source: Beilstein J Org Chem. 2019 Jan 14;15:130–6. doi: 10.3762/bjoc.15.13 (PMC6350878; doi:10.3762/bjoc.15.13)
Supplement: File 1 — Experimental, synthesis, and NMR and FTIR spectra of all the compounds. [file Beilstein_J_Org_Chem-15-130-s001.pdf]

BEILSTEIN JOURNAL OF ORGANIC CHEMISTRY

Supporting Information

for

Systematic synthetic study of four diastereomerically distinct limonene-1,2-diols and their corresponding cyclic carbonates

Hiroshi Morikawa, Jun-ichi Yamaguchi, Shun-ichi Sugimura, Masato Minamoto, Yuuta Gorou, Hisatoyo Morinaga and Suguru Motokucho

Beilstein J. Org. Chem. **2019,** *15,* 130–136. doi:10.3762/bjoc.15.13

Experimental, synthesis, and NMR and FTIR spectra of all the compounds

License and Terms: This is a supporting information file under the terms of the Creative Commons Attribution License (http://creativecommons.org/licenses/by/4.0). Please note that the reuse, redistribution and reproduction in particular requires that the authors and source are credited.
The license is subject to the *Beilstein Journal of Organic Chemistry* terms and conditions: (https://www.beilstein-journals.org/bjoc)

Table of contents

1. General ……………………………………………………………………………S3

2. Synthesis…………………………………………………………………………S4–S10

3. Characteristic data (Tables S1 and S2) ………………………………………S11

4. NMR Spectra

For **1a**
^{1}H NMR spectrum……………………………………………………………S12
^{13}C and DEPT135 spectra…………………………………………………S13
HETCOR spectra………………………………………………………………S14
^{1}H-^{1}H COSY spectra…………………………………………………S15–S16
For **1b**
^{1}H NMR spectrum……………………………………………………………S17
^{13}C and DEPT135 spectra…………………………………………………S18
HETCOR spectra………………………………………………………………S19
^{1}H-^{1}H COSY spectra…………………………………………………S20–S21
For **1c**
^{1}H NMR spectrum……………………………………………………………S22
^{13}C and DEPT135 spectra…………………………………………………S23
HETCOR spectra………………………………………………………………S24
^{1}H-^{1}H COSY spectra…………………………………………………S25–S26
For **1d**
^{1}H NMR spectrum……………………………………………………………S27
^{13}C and DEPT135 spectra…………………………………………………S28
HETCOR spectra………………………………………………………………S29
^{1}H-^{1}H COSY spectra…………………………………………………S30–S31
For comparison among **1a**–**1d**
^{1}H NMR spectra……………………………………………………………S32
^{13}C NMR spectra ……………………………………………………………S33

For **2a**
^{1}H NMR spectrum……………………………………………………………S34
^{13}C and DEPT135 spectra…………………………………………………S35
HETCOR spectra………………………………………………………………S36
^{1}H-^{1}H COSY spectra…………………………………………………S37–S38
HMBC spectra…………………………………………………………………S39
1,1-ADEQUATE spectrum……………………………………………………S40
For **2b**
^{1}H NMR spectrum……………………………………………………………S41
^{1}H NMR spectra (in benzene-d_6 and in CD_3OD)……………………S42
^{13}C and DEPT135 spectra…………………………………………………S43
HETCOR spectra………………………………………………………………S44
^{1}H-^{1}H COSY spectra…………………………………………………S45–S46
HMBC spectra…………………………………………………………………S47
1,1-ADEQUATE spectrum……………………………………………………S48
For **2c**
^{1}H NMR spectrum……………………………………………………………S49
^{13}C and DEPT135 spectra…………………………………………………S50
HETCOR spectra………………………………………………………………S51
^{1}H-^{1}H COSY spectra…………………………………………………S52–S53
HMBC spectra…………………………………………………………………S54
For **2d**
^{1}H NMR spectrum……………………………………………………………S55
^{13}C and DEPT135 spectra…………………………………………………S56
HETCOR spectra………………………………………………………………S57

^{1}H-^{1}H COSY spectra……………………………………………………………………..S58–S59
HMBC spectra…………………………………………………………………………………S60
For comparison among **2a**–**2d**
^{1}H NMR spectra……………………………………………………………………………..S61
^{13}C NMR spectra ……………………………………………………………………………S62

5. FTIR spectra ……………………………………………………………………...S63–S65

6. GC chart (typical example) ………………………………………………….......S66–S68

7. References ………………………………………………………………………......S69

1. General

(*R*)-Limonene oxide (LO) comprising a mixture of *cis*- and *trans*-isomers (43:57) was purchased from Wako (Osaka, Japan) and was used as received. Pure *trans*-LO (>99% de) and *cis*-LO (96% de) were individually prepared from the LO mixture [1]. Triphosgene (TCI, Japan), tetrabutylammonium chloride (TBAC, TCI, Japan) and lithium aluminium hydride (LAH, Wako, Japan) were used as received. Carbon dioxide was used as dry-ice. Pyridine, diethyl ether and CH_2Cl_2 were dried by distillation over CaH_2, CaH_2 and P_2O_5, respectively. All other reagents and solvents were reagent grade and used as received. Syntheses of **1a**, **1d** and **2d** were performed in a similar manner to the previous study [2].

^1H and ^{13}C NMR spectra (400 MHz ^1H; 100 MHz ^{13}C), DEPT 135 and two-dimensional NMR spectra (^1H-^1H COSY, HETCOR, HMBC) were recorded on a JEOL JNM-ECA 400 spectrometer at room temperature. The solvents were $CDCl_3$ except for **2b** in CD_3OD or in benzene-d_6 in Supporting Information page S42. The concentration was adjusted to 5–15 mg mL^{-1} dependent on the measurements. Chemical shifts were determined using tetramethylsilane as an internal standard. All the chemical shift values (ppm) in ^{13}C NMR analyses of the LM5CCs and LMdiols are listed in Tables 1 and 2. 1,1-ADEQUATE spectra were measured at the Office for Research Initiative and Development / Common Facilities Division in Nagasaki University by a Varian NMR System 500 at 24 °C in $CDCl_3$, and operated using VnmrJ 4.2 Rev. A software. The concentration was adjusted to approximately 100 mg mL^{-1}.

FTIR spectra were recorded on a JASCO FTIR 460 spectrometer, and the transmission spectra were measured from KBr discs. High-resolution mass spectra (HRMS) were recorded using a JEOL JMS-700N instrument using electron ionisation

(EI) mass spectrometry, and measured at the Office for Research Initiative and Development / Common Facilities Division in Nagasaki University. Melting point (mp) was measured on a YANAKO MP-500D hot-stage apparatus. Optical rotations were measured in a 100 mm length cell on a JASCO Model P-2200 digital polarimeter at 25 °C, and an average value from ten measurements was calculated. Gas chromatography (GC) analysis was performed on a SHIMADZU GC2014. The injector and detector temperatures were set at 230 and 350 °C, respectively, The temperature of column (Rxi-5Sil MS column, GL Sciences Inc., Japan) was initially at 100 °C for 1min, then raised to 330 °C at 20 °C /min, and finally hold at 330 °C. Kovats retention index of each compound was estimated according to the literature [3] as references to mixed hydrocarbon standards (C9–C40) in *n*-hexane (GL Sciences Inc., Japan).

Thin-layer chromatography (TLC) was performed using GF254 silica gel plates (Merck). Acidic *p*-anisaldehyde was used for TLC visualisation.

2. Synthesis

Synthesis of carbonate 1a from *trans*-LO

Conditions (a) in Scheme 3

LM5CC **1a** was synthesised by a modification of the procedure from our previous report [2]. Briefly, a mixture of pure *trans*-LO (762 mg, 5.01 mmol), TBAC (140 mg, 0.504 mmol), and dry-ice was heated in a autoclave (Type TVS-N2, Taiatsu Glass Kogyo Co., Japan) with stirring at 100 °C for 72 h under 5 MPa CO_2. After carbonation, the reaction mixture was purified using SiO_2 column chromatography (*n*-hexane/ethyl acetate (10:1, v/v) as an eluent) to afford **1a** (829 mg, 4.23 mmol) as a white solid in an 84% yield. Though the mp and ^{1}H and ^{13}C NMR were measured

previously [2], their measurements were re-performed in this study for more detail characterization.

1a [2,4,5]: mp 35–37 °C (lit [2]; 37–38 °C); ^{1}H NMR ($CDCl_3$) δ 4.76 (q, *J* = 1.4 Hz, 1H), 4.72 (s, 1H), 4.37 (dd, *J* = 9.6 and 6.9 Hz, 1H), 2.31 (ddd, *J* = 15.5, 3.7 and 3.7 Hz, 1H), 2.28–2.22 (m, 1H), 1.92 (dddd, *J* = 11.9, 11.9, 3.2 and 3.2 Hz, 1H), 1.72 (s, 3H), 1.71–1.59 (m, 2H), 1.51–1.40 (m, 2H), 1.47 (s, 3H). The IR, HRMS data and the optical rotation value were reported previously [2].

Synthesis of carbonate 1d from *cis*-LO

Conditions (a) in Scheme 3

In a similar manner to that used for **1a**, **1d** [2] was synthesised from a mixture of pure *cis*-LO (766 mg, 5.03 mmol), TBAC (136 mg, 4.89 mmol) and dry-ice, which was reacted under 5 MPa CO_2 at 100 °C for 72 h. The carbonate **1d** (301 mg, 1.53 mmol) was obtained as a white solid in a 30% yield following SiO_2 column chromatography (*n*-hexane/ethyl acetate (10:1, v/v) as an eluent). Though the mp and ^{1}H and ^{13}C NMR were measured previously [2], their measurements were re-performed in this study for more detail characterization.

1d[2]: mp 40–41 °C(lit [2]; 40–41 °C); ^{1}H NMR ($CDCl_3$) δ 4.78 (q, *J* = 1.4 Hz, 1H), 4.72 (m, 1H), 4.44 (dd, *J* = 3.2 and 2.3 Hz, 1H), 2.31–2.22 (m, 2H), 2.01 (ddd, *J* = 13.3, 4.6 and 4.1 Hz, 1H), 1.88–1.79 (m, 2H), 1.74 (s, 3H), 1.56 (ddd, *J* = 14.3, 13.3 and 3.6 Hz, 1H), 1.51 (s, 3H), 1.25–1.15 (m, 1H). The IR, HRMS data and the optical rotation value were reported previously [2].

Synthesis of carbonate 1a from diol 2a

Conditions (c) in Scheme 3

In a 100 mL round-bottomed flask, **2a** (341.7 mg, 2.007 mmol) and dry pyridine (652.9 mg, 8.254 mmol) were dissolved in dry CH_2Cl_2 (18 mL). Triphosgene (289.7 mg, 0.9763 mmol) in dry CH_2Cl_2 (2 mL) was added to the solution under cooling in an ice bath. After stirring at room temperature for 2 h under N_2 atmosphere, the consumption of **2a** was confirmed by TLC. Then, water (20 mL) was added to the solution. The organic layer was separated and washed with saturated aqueous NH_4Cl solution. The organic layer was collected, dried over anhydrous $MgSO_4$ and filtered. After evaporation, the crude product was purified by SiO_2 column chromatography eluted with *n*-hexane/ethyl acetate (10:1, v/v) to obtain **1a** (370.5 mg, 1.888 mmol) in a 94% yield. The spectroscopic data were identical to those of an authentic sample prepared from *trans*-LO and CO_2.

Synthesis of carbonate 1d from diol 2d

Conditions (c) in Scheme 3

LM5CC **1d** was synthesised in a similar manner to that used to prepare **1a** from **2a** and triphosgene. From **2d** (346.8 mg, 2.037 mmol) and triphosgene (286.0 mg, 0.9638 mmol), **1d** (374.5 mg, 1.908 mmol) was obtained as a white solid in a 94% yield. The spectroscopic data were identical to those of the authentic sample prepared from *cis*-LO and CO_2.

Synthesis of carbonate 1b from diol 2b

Conditions (c) in Scheme 3

LM5CC **1b** was synthesised in a similar manner to **1a**. From **2b** (340.5 mg, 2.000 mmol) and triphosgene (623.6 mg, 2.102 mmol), **1b** (96.1 mg, 0.489 mmol) was obtained as a white solid in a 24% yield.

1b: mp 35–36 °C; IR (KBr) $\nu_{C=O}$ 1805 cm^{-1}; ^{1}H NMR ($CDCl_3$) δ 4.98 (bs, 1H), 4.89 (s, 1H), 4.37 (dd, *J* = 13.7 and 3.7 Hz, 1H), 2.61 (t, *J* = 5.0 Hz,1H), 2.30 (ddd, *J* = 12.4, 1.8 and 1.8 Hz, 1H), 2.17–2.12 (m, 1H) 2.13–2.03 (m, 1H), 1.98–1.95 (m, 1H), 1.89 (ddd, *J* = 12.8, 12.8 and 6.0 Hz, 1H), 1.79 (s, 3H), 1.77–1.71 (m, 1H), 1.41 (s, 3H); HRMS (EI, *m*/*z*) $[M]^+$ Calcd. for $C_{11}H_{16}O_3$, 196.1099; found, 196.1099; $[\alpha]^{25}{}_{D}$ = -25.4 (*c* 1.00, $CHCl_3$).

Synthesis of carbonate 1c from diol 2c

Conditions (c) in Scheme 3

LM5CC **1c** was synthesised in a similar manner to **1a**. From **2c** (341.3 mg, 2.004 mmol) and triphosgene (609.0 mg, 2.053 mmol), **1c** (227.3 mg, 1.158 mmol) was obtained as a white solid in a 58% yield.

1c: mp 34–36 °C; IR (KBr) $\nu_{C=O}$ 1806 cm^{-1}; ^{1}H NMR ($CDCl_3$) δ 4.80 (q, *J* = 1.4 Hz, 1H), 4.78 (s, 1H), 4.17 (dd, *J* = 12.8 and 3.7 Hz, 1H), 2.28 (dddd, *J* = 12.4, 11.9, 4.6 and 4.1 Hz, 1H), 2.20 (ddd, *J* = 11.9, 3.7 and 3.2 Hz, 1H), 2.11–2.06 (m, 1H), 1.96–1.85 (m, 2H), 1.76 (s, 3H), 1.75 (ddd, *J* = 12.8, 11.9 and 11.9 Hz, 1H), 1.55–1.45(m, 1H), 1.38 (s, 3H); HRMS (EI, *m*/*z*) $[M]^+$ Calcd. for $C_{11}H_{16}O_3$, 196.1099; found, 196.1092; $[\alpha]^{25}{}_{D}$ = +3.3 (*c* 1.00, $CHCl_3$).

Synthesis of diol 2a from carbonate 1a

Conditions (b) in Scheme 3

In a 200-mL round-bottomed flask, **1a** (2.0177 g, 10.28 mmol) was dissolved in dry diethyl ether (40 mL) under N_2 atmosphere. The flask was immersed into an ice bath and LAH (726.7 mg, 19.14 mmol) was added portionwise with cooling. After stirring for 2 h at room temperature, the consumption of **1a** was confirmed by TLC. Then, ethyl acetate (100 mL) and brine (100 mL) were carefully added to the mixture. The

organic layer was separated, dried over anhydrous $MgSO_4$, and filtered. After evaporation and purification using SiO_2 column chromatography (*n*-hexane/ethyl acetate, 1:5, v/v), **2a** (1.6135 g, 9.4773 mmol) was obtained as a white solid in a 92% yield.

2a: mp 75 °C; IR (KBr) ν_{OH} 3348, $\nu_{C\text{-}H}$ 2933, 2860 cm^{-1}; ^1H NMR ($CDCl_3$) δ 4.71–4.69 (m, 2H), 3.41(dd, *J* = 11.4 and 4.6 Hz, 1H), 1.96 (dddd, *J* = 12.4, 11.9, 3.2 and 3.2Hz, 1H), 1.84 (ddd, *J* = 13.7, 3.2 and 3.2 Hz, 1H), 1.79 (ddd, *J* = 12.4, 3.3 and 3.2 Hz, 1H), 1.73 (s, 3H), 1.54–1.48 (m, 3H), 1.42–1.34 (m, 1H), 1.27 (s, 3H); HRMS (EI, *m*/*z*) $[M]^+$ Calcd. for $C_{10}H_{18}O_2$, 170.1307; found, 170.1307; $[\alpha]^{25}{}_D$ = +4.8 (*c* 1.00, $CHCl_3$).

Synthesis of diol 2b from carbonate 1b

Conditions (b) in Scheme 3

Diol **2b** was synthesised in a similar manner to **2a**. Carbonate **1b** (103.3 mg, 0.5264 mmol) and LAH (41.2 mg, 1.09 mmol) were reacted to obtain **2b** (75.5 mg, 0.443 mmol) as a white solid in an 84% yield. The spectroscopic data were identical to those of a sample prepared from *cis*-LO and water.

2b: mp 72–73 °C (lit [6]; 69–71 °C, lit [7]; 68–70 °C); IR (KBr) ν_{OH} 3330, $\nu_{C\text{-}H}$ 2938, 2869 cm^{-1}; ^1H NMR ($CDCl_3$) δ 4.73 (s, 2H), 3.63 (t, *J* = 3.2 Hz, 1H), 2.26 (ddd, *J* = 11.8, 4.2 and 3.2Hz, 1H), 1.93 (ddd *J* = 13.7, 4.2 and 4.2 Hz, 1H), 1.78–1.72 (m, 1H), 1.73 (s, 3H), 1.66 (ddd, *J* = 13.7, 4.2 and 3.2 Hz, 1H), 1.60–1.50 (m, 3H), 1.26 (s, 3H); HRMS (EI, *m*/*z*) $[M]^+$ Calcd. for $C_{10}H_{18}O_2$, 170.1307; found, 170.1302; $[\alpha]^{25}{}_D$ = +25.8 (*c* 1.00, $CHCl_3$), (lit [6]; $[\alpha]^{18}{}_D$ = +27.2 (*c* 0.49, $CHCl_3$), lit [7]; $[\alpha]^{20}{}_D$ = +18.1 (*c* 0.01, $CHCl_3$)).

Synthesis of diol 2c from carbonate 1c

Conditions (b) in Scheme 3

Diol **2c** was synthesised in a similar manner to **2a**. From **1c** (149.3 mg, 0.7608 mmol) and LAH (58.6 mg, 1.54 mmol), **2c** (122.4 mg, 0.7189 mmol) was obtained as a white solid in a 94% yield. The spectroscopic data were identical to those of a sample prepared from *trans*-LO and water.

2c: mp 73–74 °C (lit [7]; 74–76 °C, lit [8]; 71–73 °C); IR (KBr) ν_{OH} 3363, $\nu_{C\text{-}H}$ 2936, 2864 cm^{-1}; ^{1}H NMR ($CDCl_3$) δ 4.71 (s, 2H), 3.58 (dd, *J* = 11.4 and 4.1 Hz, 1H), 2.07 (dddd, *J* = 12.4, 12.4, 3.7 and 3.7 Hz, 1H), 1.95–1.89 (m, 1H), 1.80 (ddd, *J* = 12.8, 3.2 and 3.2 Hz, 1H), 1.73(s, 3H), 1.73–1.69 (m, 1H), 1.49 (ddd, *J* = 13.7, 12.8 and 4.6 Hz, 1H), 1.36–1.24 (m, 2H), 1.21 (s, 3H); HRMS (EI, *m*/*z*) $[M]^+$ Calcd. for $C_{10}H_{18}O_2$, 170.1307; found, 170.1311; $[\alpha]^{25}{}_{D}$ = -5.0 (*c* 1.00, $CHCl_3$), (lit[7]; $[\alpha]^{20}{}_{D}$ = -5.5 (*c* 0.01, $CHCl_3$), lit [8]; $[\alpha]^{20}{}_{D}$ = -6.6 (*c* 1.099, $CHCl_3$)).

Synthesis of diol 2d from carbonate 1d

Conditions (b) in Scheme 3

The synthesis was reported previously in ref.[2]. Though the mp and ^{1}H and ^{13}C NMR were measured previously [2], their measurements were re-performed in this study for more detail characterization.

2d[2]: mp 46–47 °C (lit [2]; 48–49 °C); ^{1}H NMR ($CDCl_3$) δ 4.72 (m, 1H), 4.71 (m, 1H), 3.66 (t, *J* = 3.5Hz, 1H), 2.35 (dddd, *J* = 11.9, 11.9, 3.7 and 3.7 Hz, 1H), 1.93–1.88 (m, 1H), 1.87–1.80 (m, 1H), 1.72 (s, 3H), 1.74–1.68 (m, 1H), 1.58–1.51 (m, 2H), 1.30 (dddd, *J* = 13.3, 13.3, 11.9 and 3.7 Hz, 1H), 1.25 (s, 3H). The IR, HRMS data and the optical rotation value were reported previously [2].

Synthesis of diol 2b from *cis*-LO

Conditions (e) in Scheme 3

A mixture of pure *cis*-LO (465.4 mg, 3.057 mmol) and water (2.66 g, 148 mmol) in a 15 mL test tube was stirred at 90 ° C for 12 h. After cooling to ambient temperature, a white precipitate appeared and was collected by filtration (203.9 mg). The filtrate was extracted with diethyl ether (10 mL) twice. The organic layer was isolated and dried over anhydrous $MgSO_4$. After evaporation, the residue was purified using SiO_2 column chromatography (*n*-hexane/ethyl acetate, 2:1, v/v) to obtain a white solid (161.5 mg). This solid was combined with the filtered solid to obtain **2b** (365.4 mg, 2.146 mmol) as a white solid in a 70% yield overall.

Synthesis of diol 2c from *trans*-LO

Conditions (d) in Scheme 3

A mixture of pure *trans*-LO (468.2 mg, 3.076 mmol), water (1.34 g, 74.4 mmol) and 1,4-dioxane (1.34 mL) was sealed in a 10 mL pressure-resistant glass tube. This mixture was stirred at 120 °C for 72 h. After cooling, the pressure was released. Brine (10 mL) was added to the mixture, and it was extracted twice with ethyl acetate (15 mL). The organic layer was isolated and dried over anhydrous $MgSO_4$. The filtrate was evaporated and the residue was purified using SiO_2 column chromatography (*n*-hexane/ethyl acetate, 1:1, v/v). The diol **2c** (97.4 mg, 0.572 mmol) was isolated as a white solid in a 19% yield along with **2b** (305.0 mg, 58%).

3. Characteristic data

Some data have been already shown in the aforementioned experimental section of this Supporting Information and cited from ref.[2].

Table S1: Characteristic data of LM5CCs, **1a–d**

	compounds			
	**1a**	**1b**	**1c**	**1d**
Melting point (^{o}C)	35–37	35–36	34–36	40–41
IR absorption band (KBr) $\nu_{C=O}$ (cm^{-1})	1794[c]	1805	1806	1807[c]
specific rotation $[\alpha]^{25}_{D}$ (*c* 1.00, $CHCl_3$)	+53.7[c]	-25.4	+3.3	-15.8[c]
R_f on TLC[a]	0.35	0.35	0.35	0.35
Kovats retention index[b]	1618	1699	1665	1620

[a]developing with *n*-hexane/ethyl acetate = 5/1 (*v*/*v*). [b]determined by GC using hydrocarbon standards as reference samples. [c]cited from ref.[2]

Table S2: Characteristic data of LMdiols, **2a–d**

	compounds			
	**2a**	**2b**	**2c**	**2d**
Melting point (^{o}C)	75	72–73	73–74	46–47
IR absorption band (KBr) ν_{OH} (cm^{-1})	3348	3330	3363	3322[c]
specific rotation $[\alpha]^{25}_{D}$ (*c* 1.00, $CHCl_3$)	+4.8	+25.8	-5.0	+28.3[c]
R_f on TLC[a]	0.40	0.40	0.20	0.35
Kovats retention index[b]	1348	1363	1373	1359

[a]developing with *n*-hexane/ethyl acetate = 1/1 (*v*/*v*). [b]determined by GC using hydrocarbon standards as reference samples. [c]cited from ref.[2]

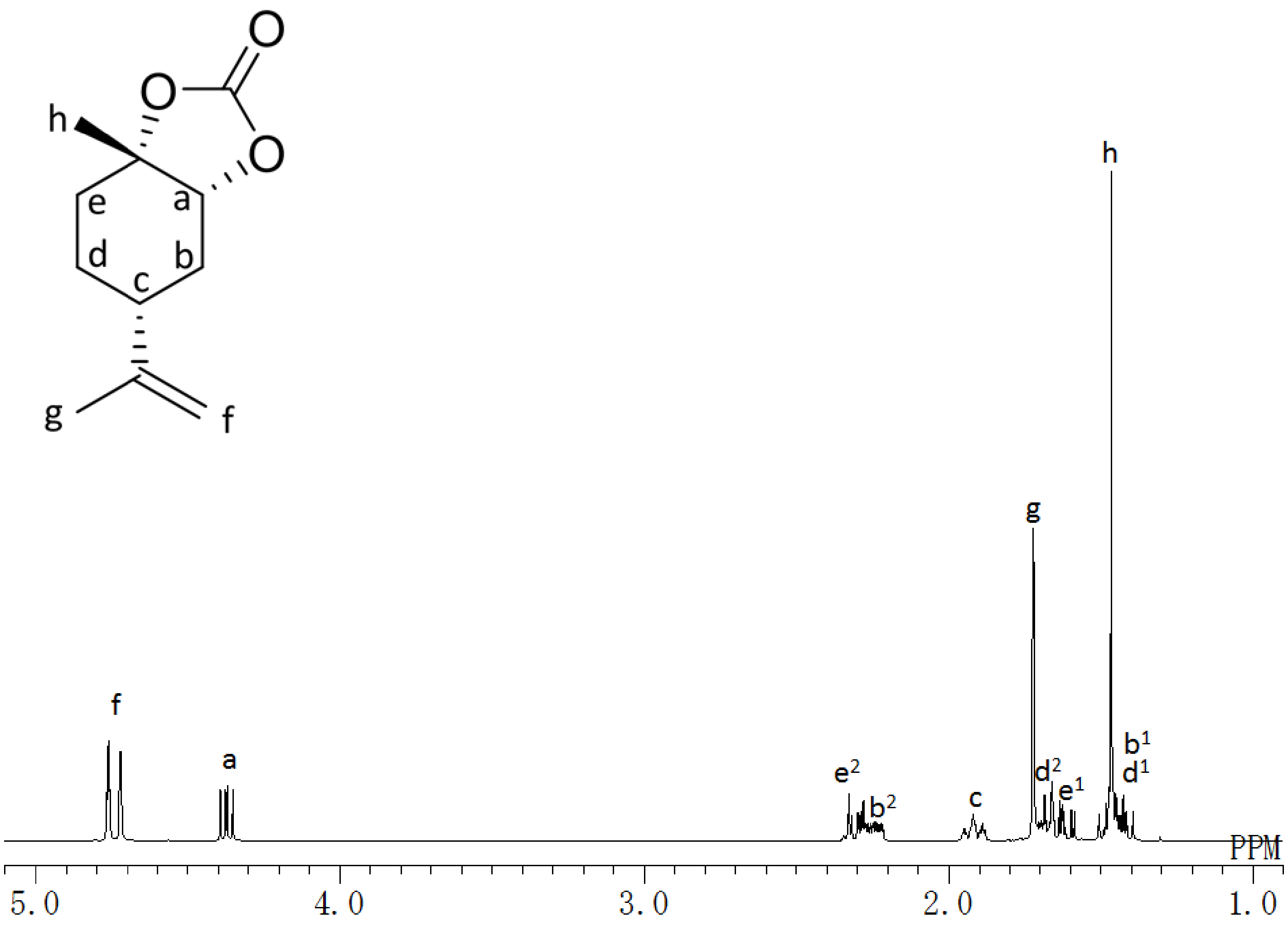

^{1}H NMR spectrum of **1a** in $CDCl_3$.

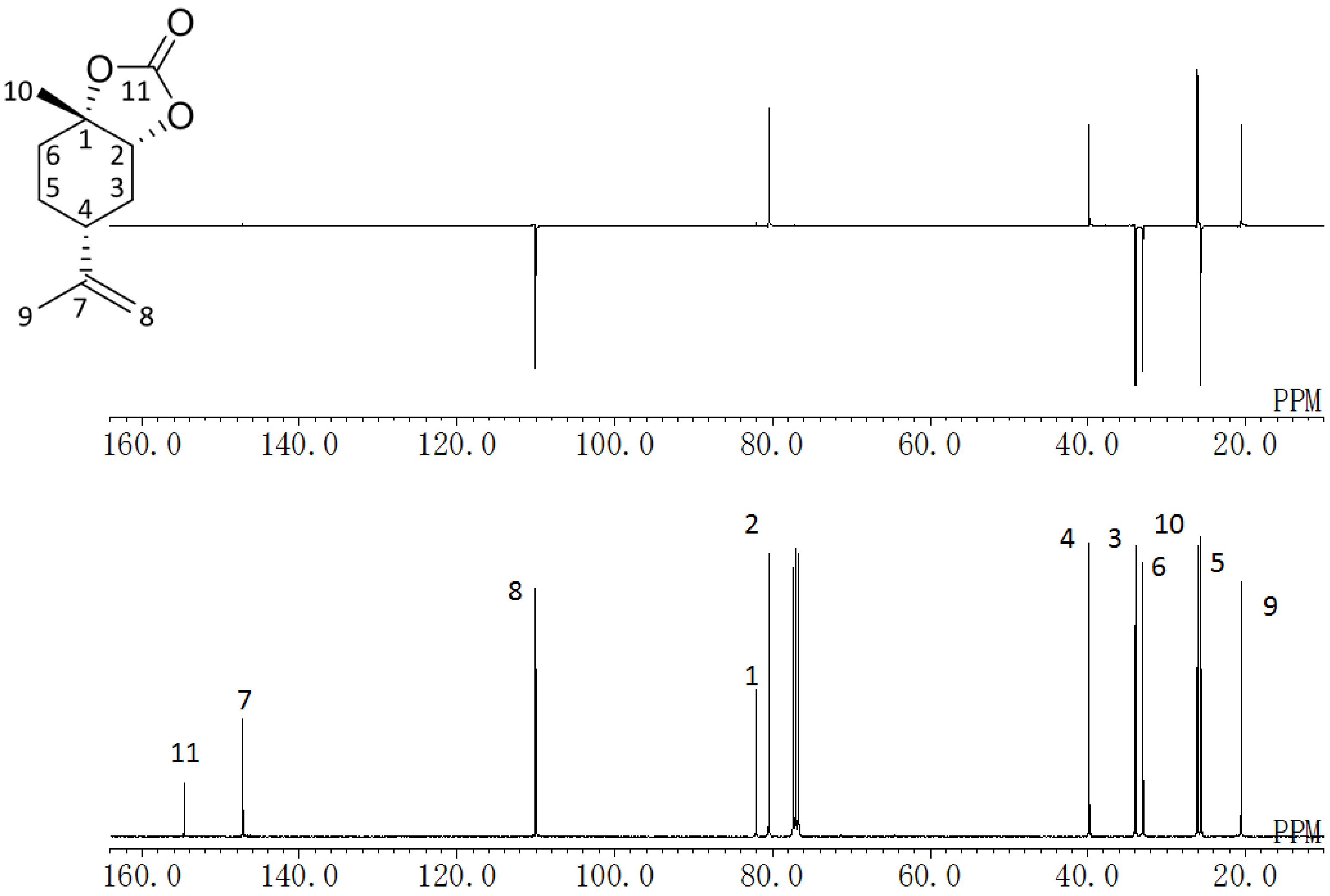

(top) DEPT135 and (bottom) ^{13}C NMR spectra of **1a** in $CDCl_3$.

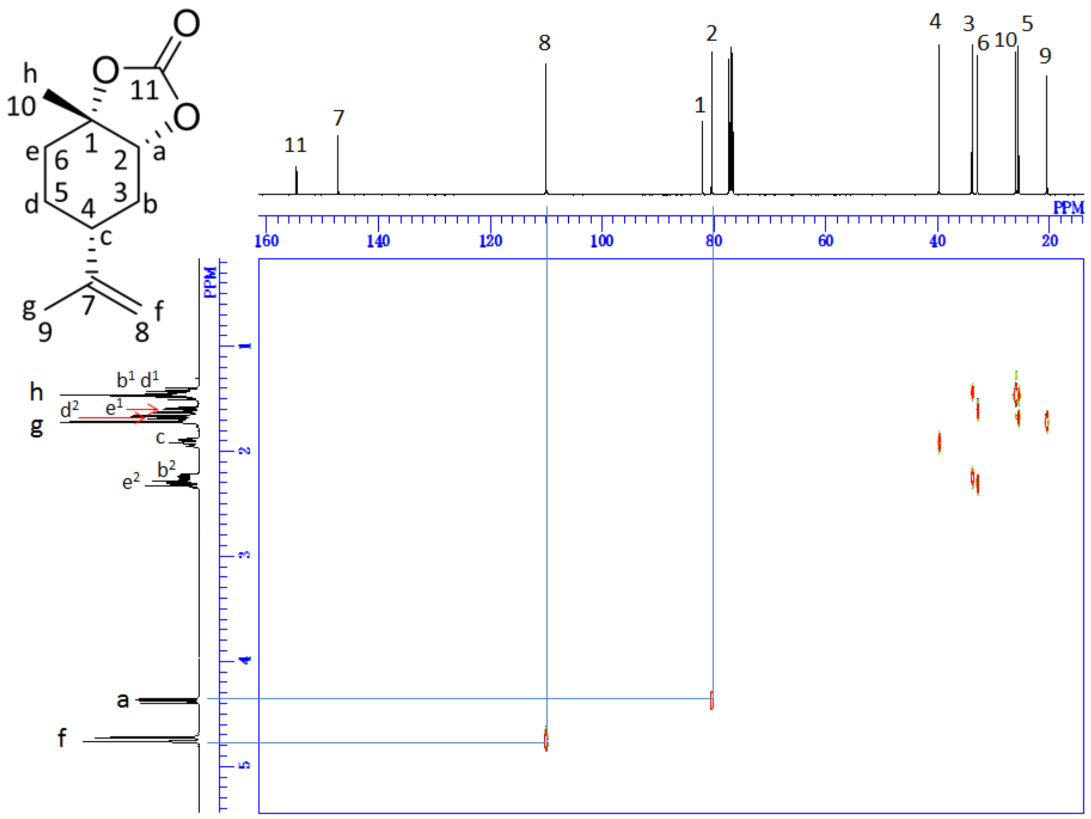

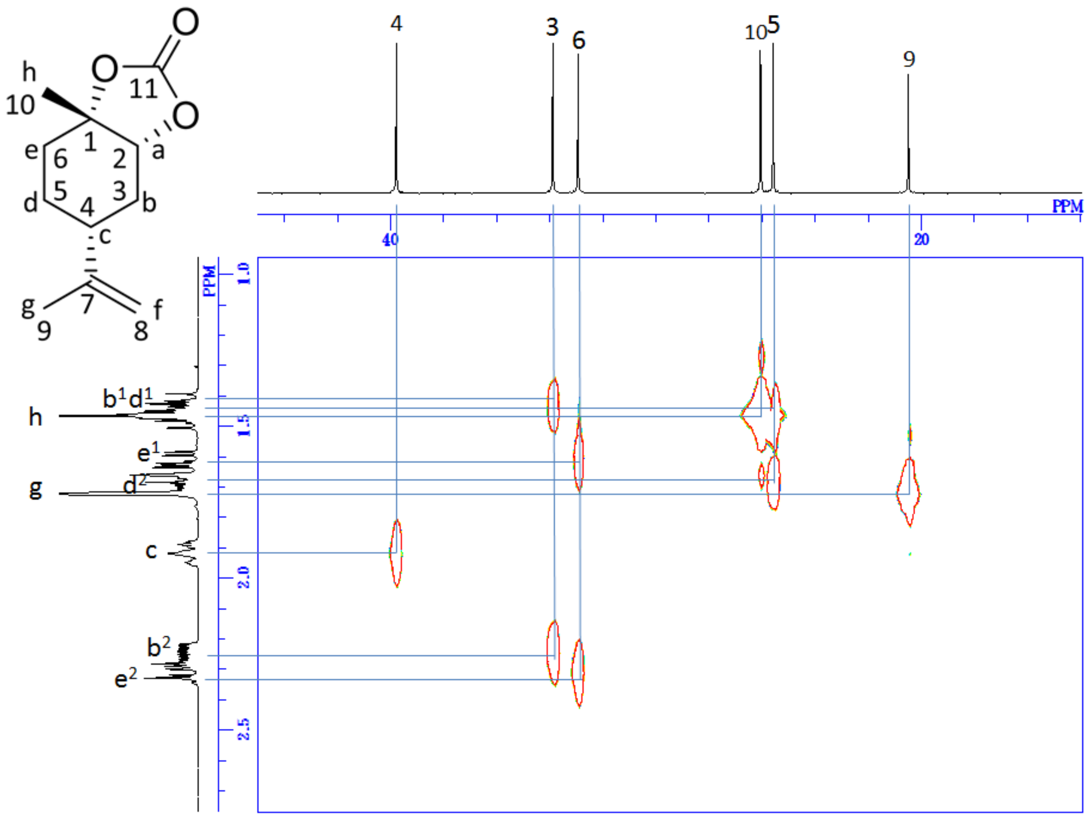

HETCOR spectra of **1a** in $CDCl_3$; (top) full range and (bottom) selected range.

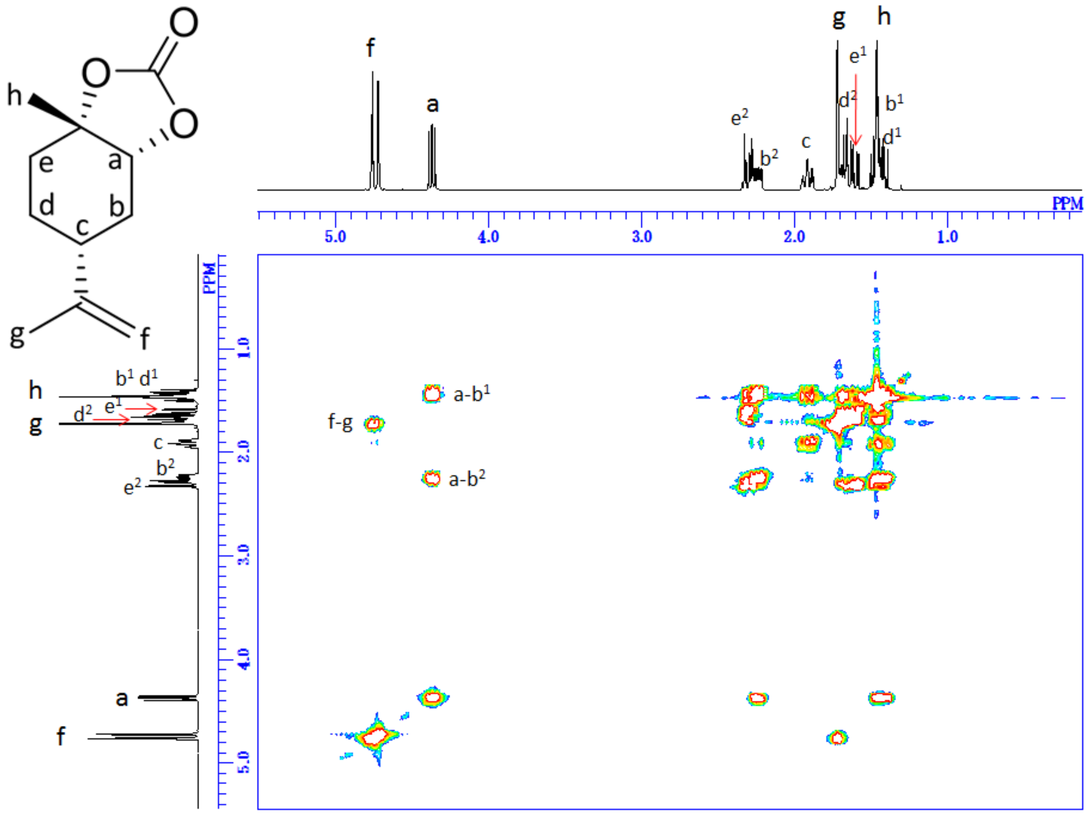

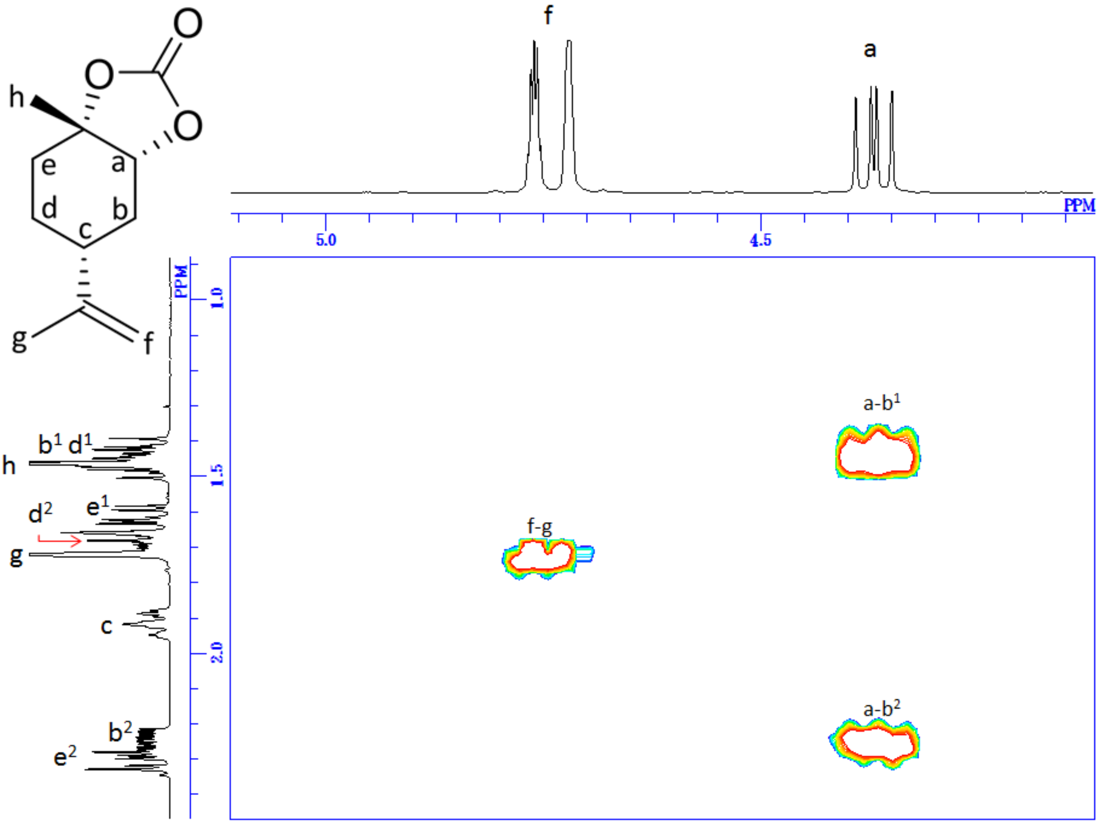

^{1}H-^{1}H COSY spectra of **1a** in $CDCl_3$; (top) full range and (bottom) selected range.

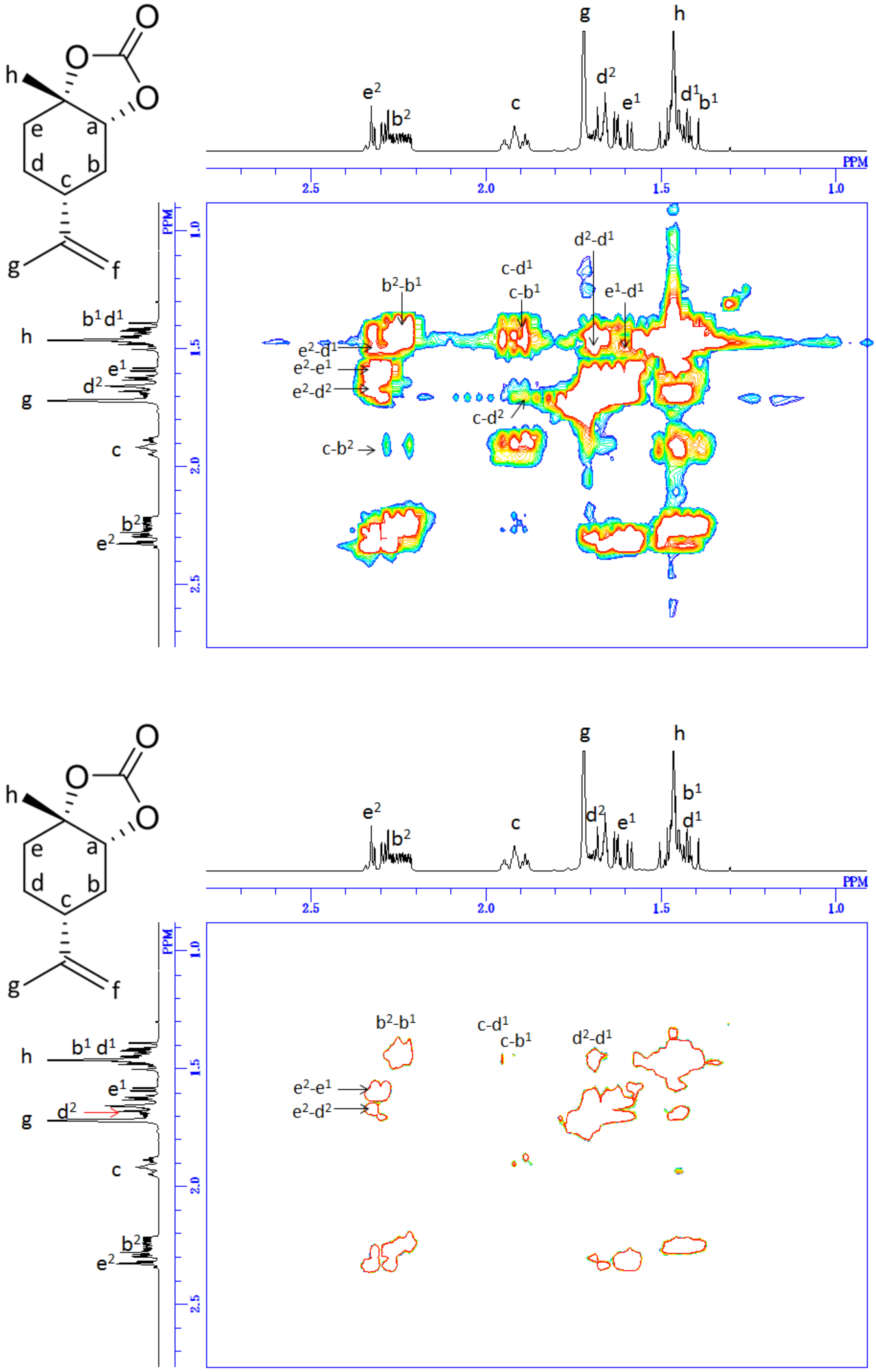

^{1}H-^{1}H COSY spectra of **1a** in $CDCl_3$; selected range with (top) high sensitivity and (bottom) low sensitivity.

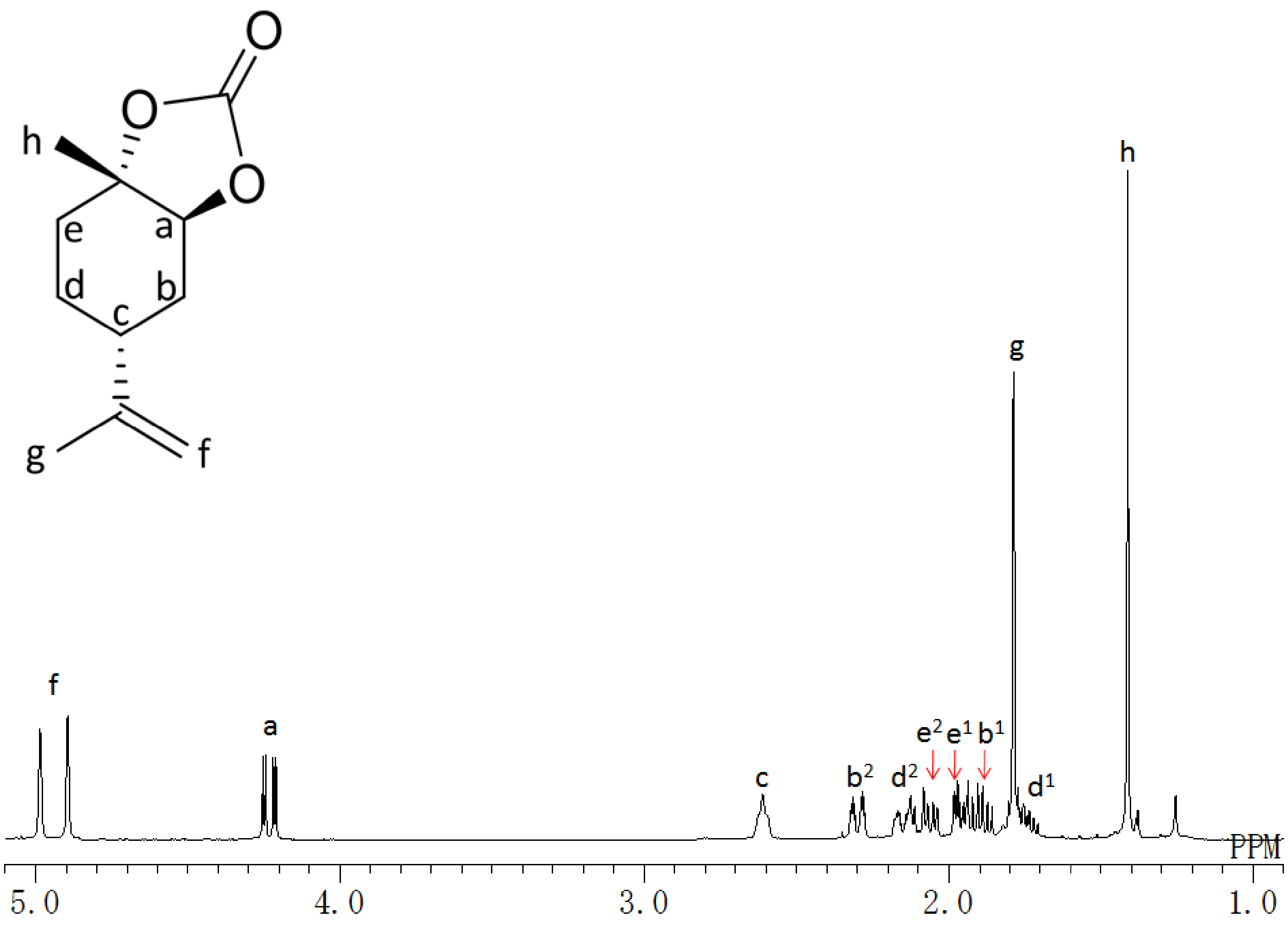

^{1}H NMR spectrum of **1b** in $CDCl_3$.

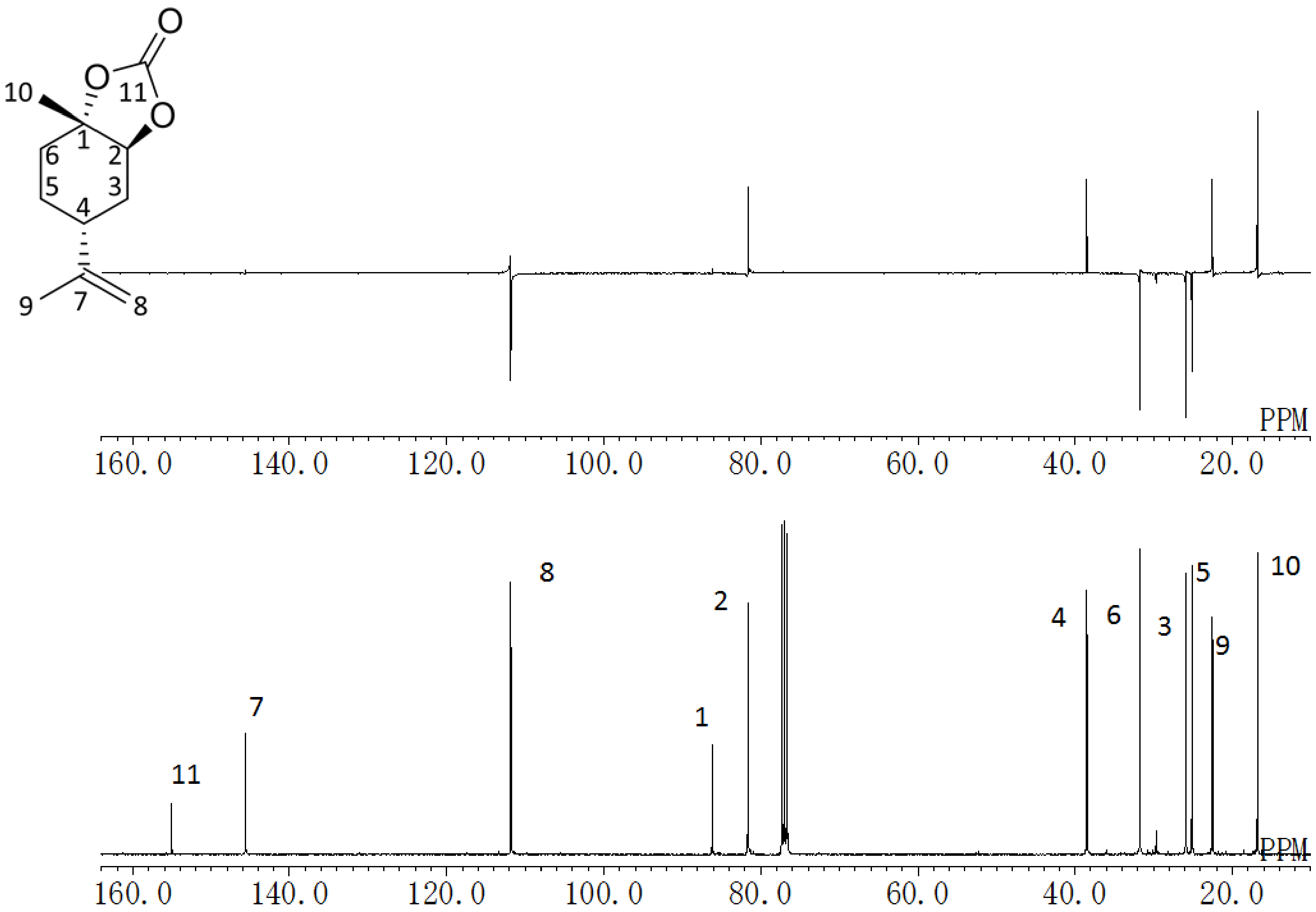

(top) DEPT135 and (bottom) ^{13}C NMR spectra of **1b** in $CDCl_3$.

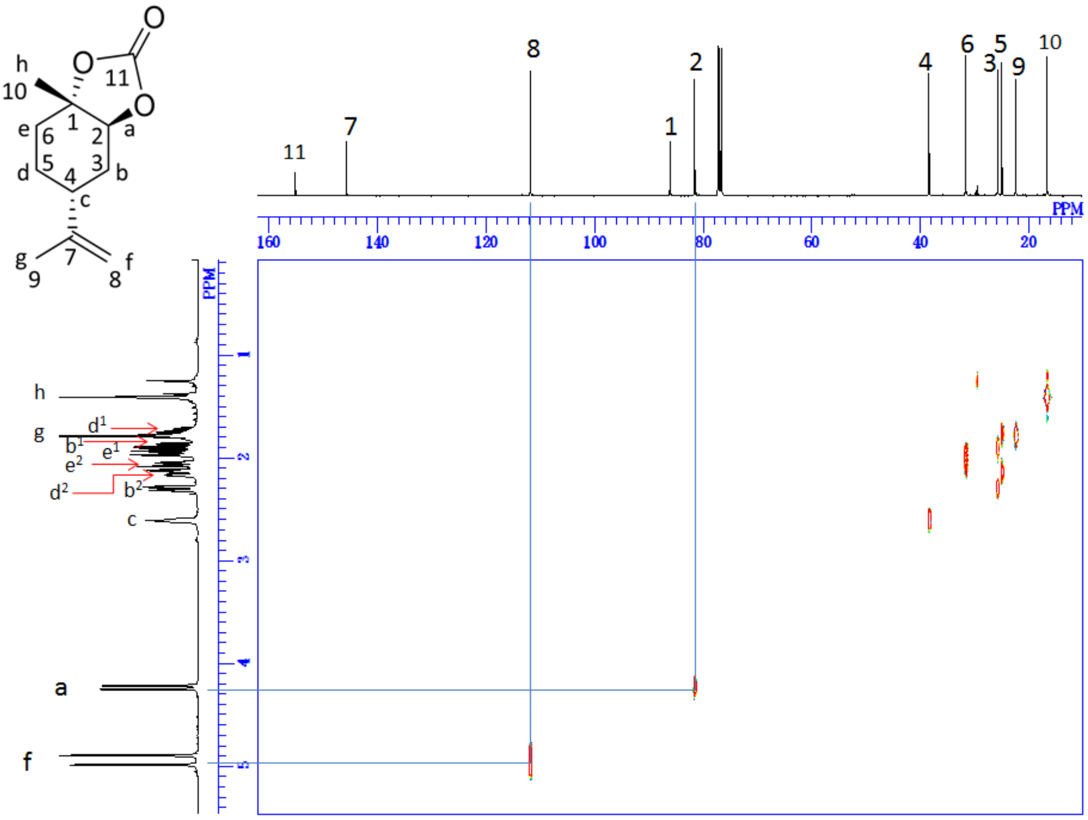

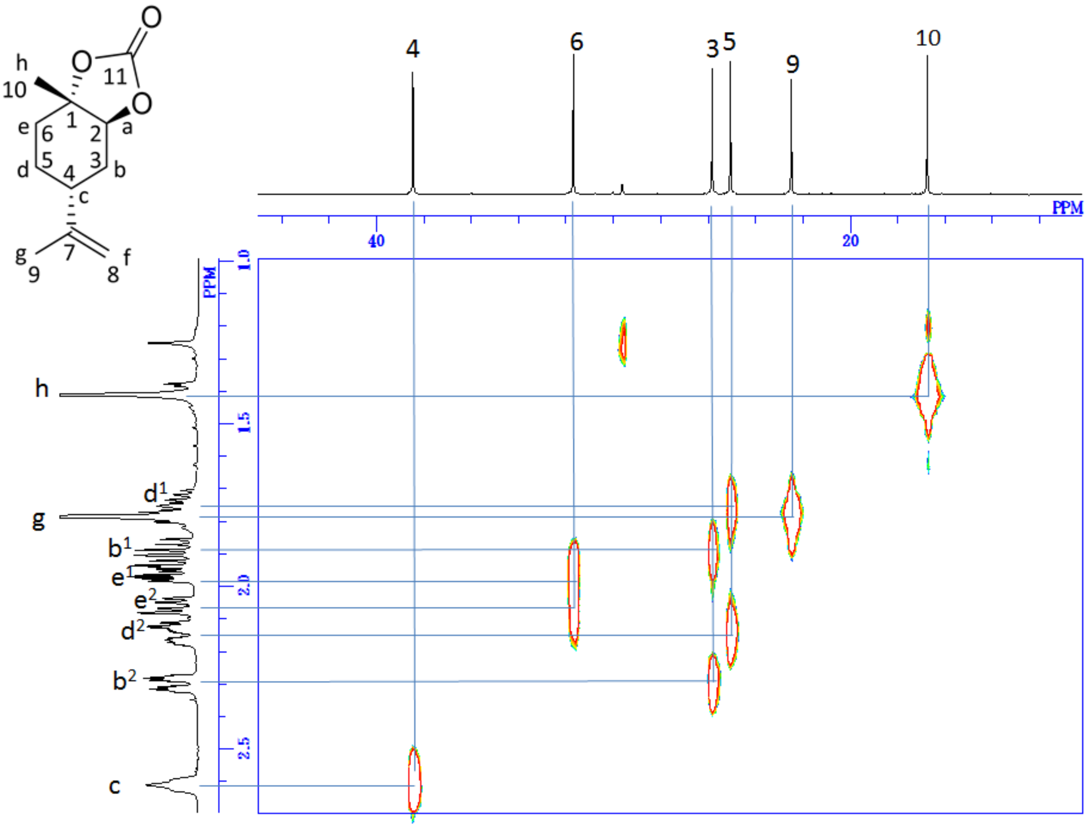

HETCOR spectra of **1b** in $CDCl_3$; (top) full range and (bottom) selected range.

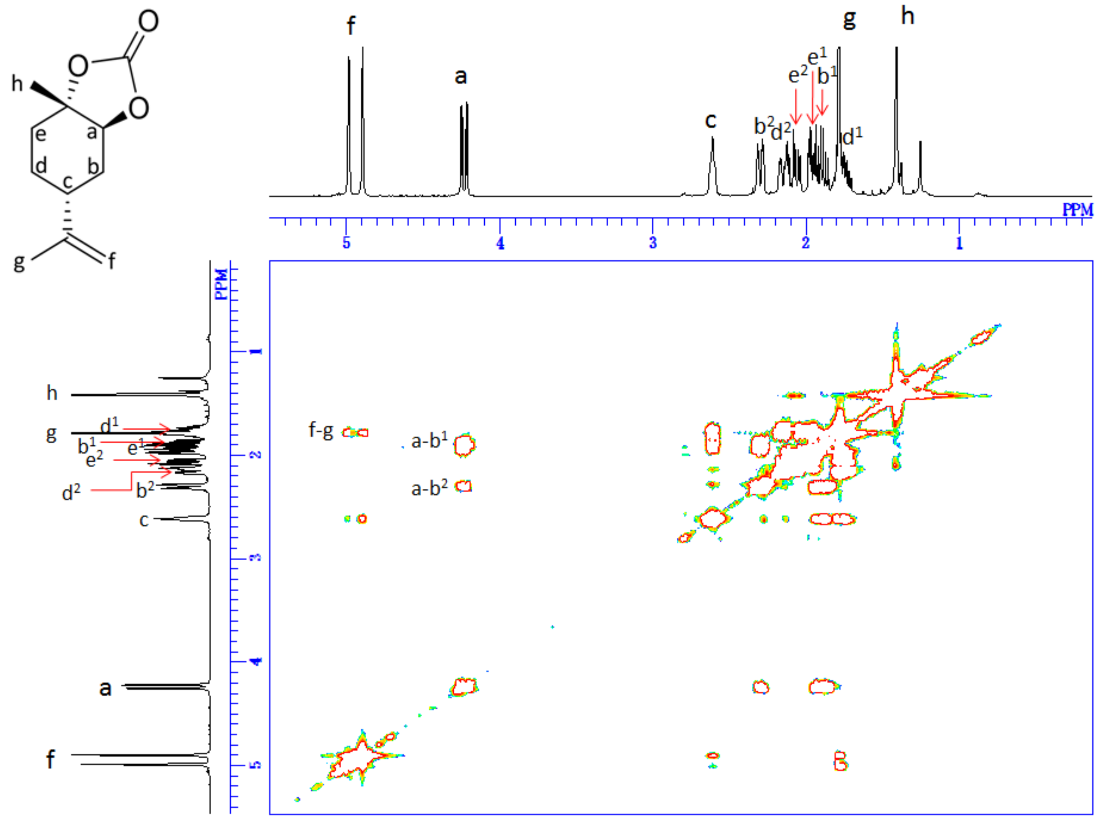

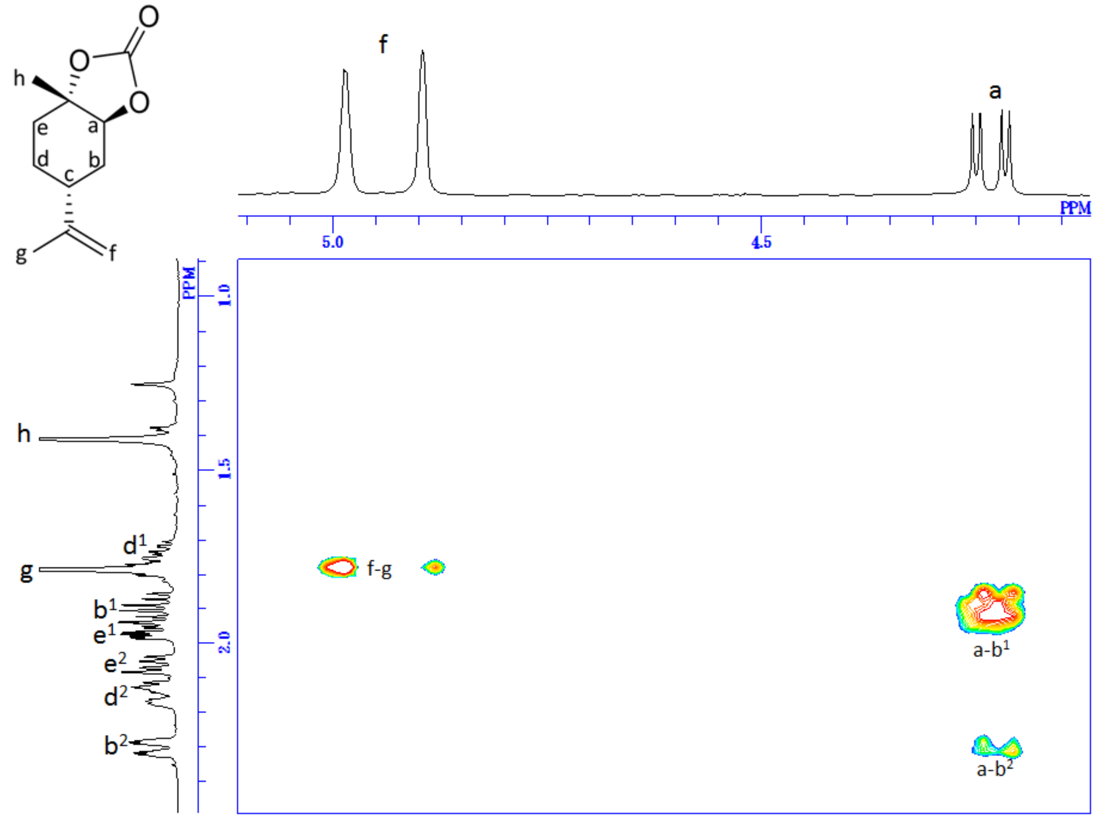

^{1}H-^{1}H COSY spectra of **1b** in $CDCl_3$; (top) full range and (bottom) selected range.

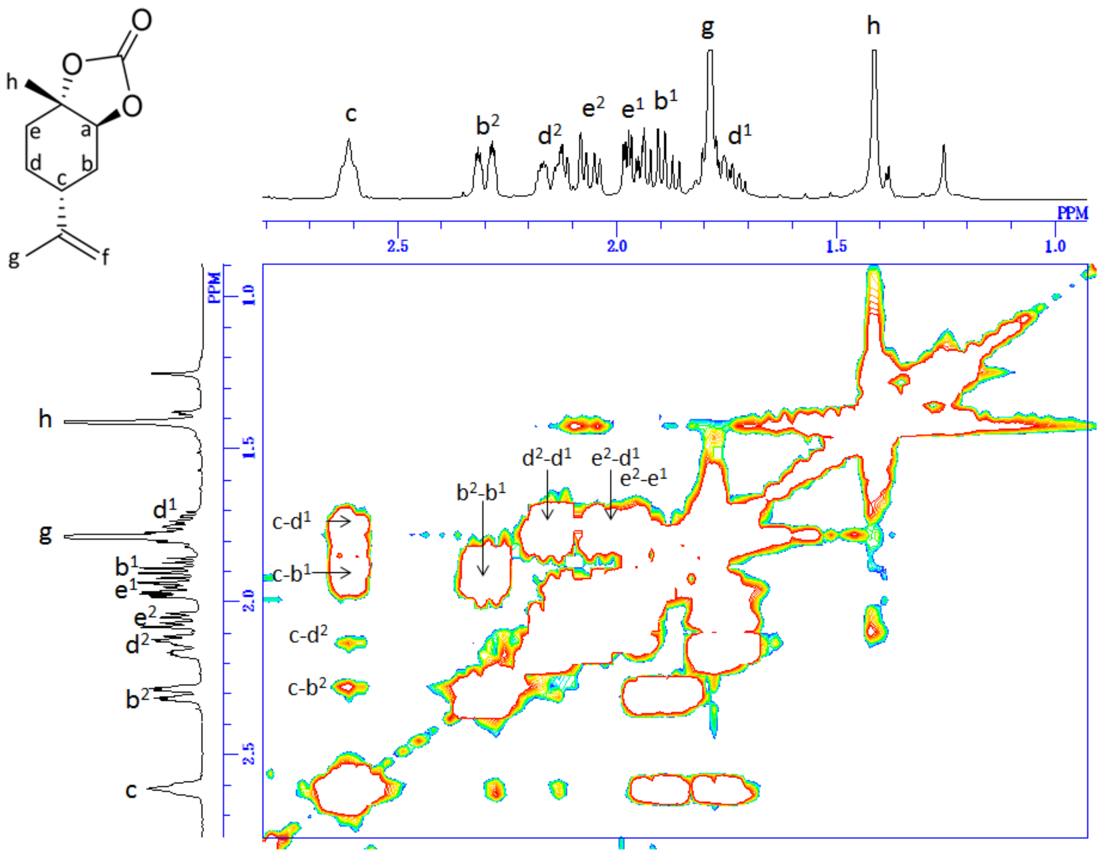

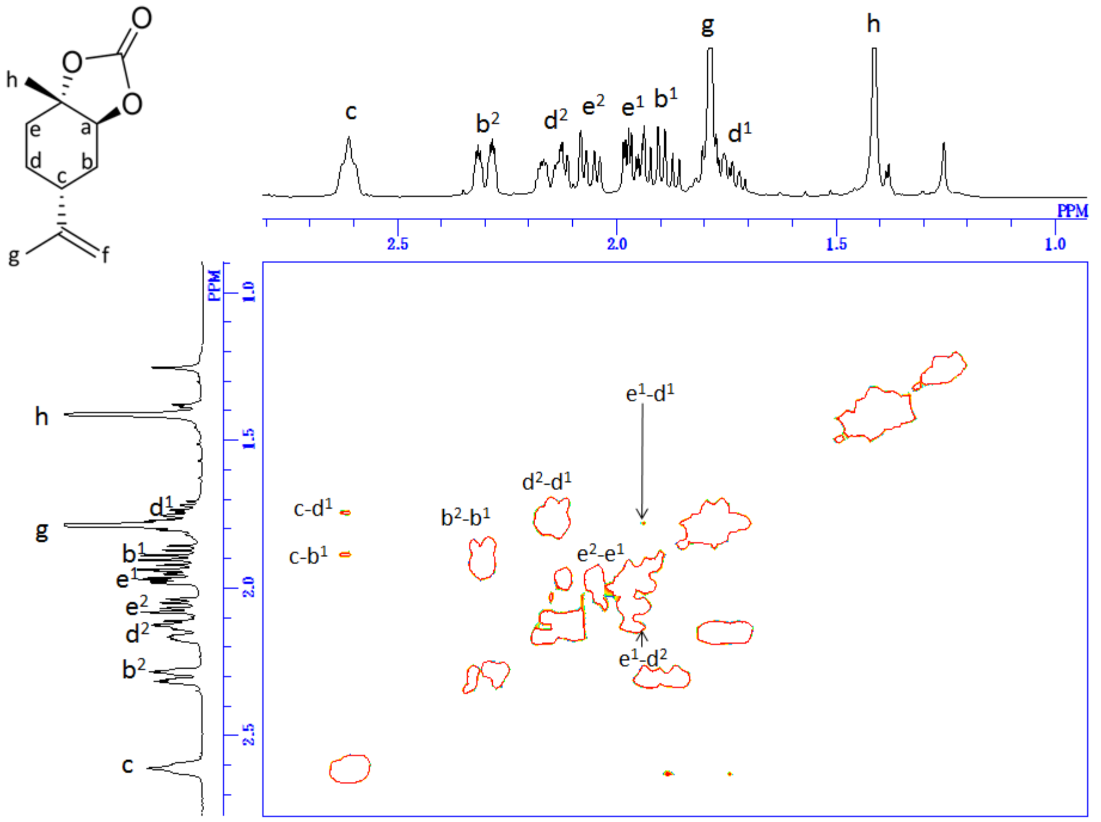

^{1}H-^{1}H COSY spectra of **1b** in $CDCl_3$; selected range with (top) high sensitivity and (bottom) low sensitivity.

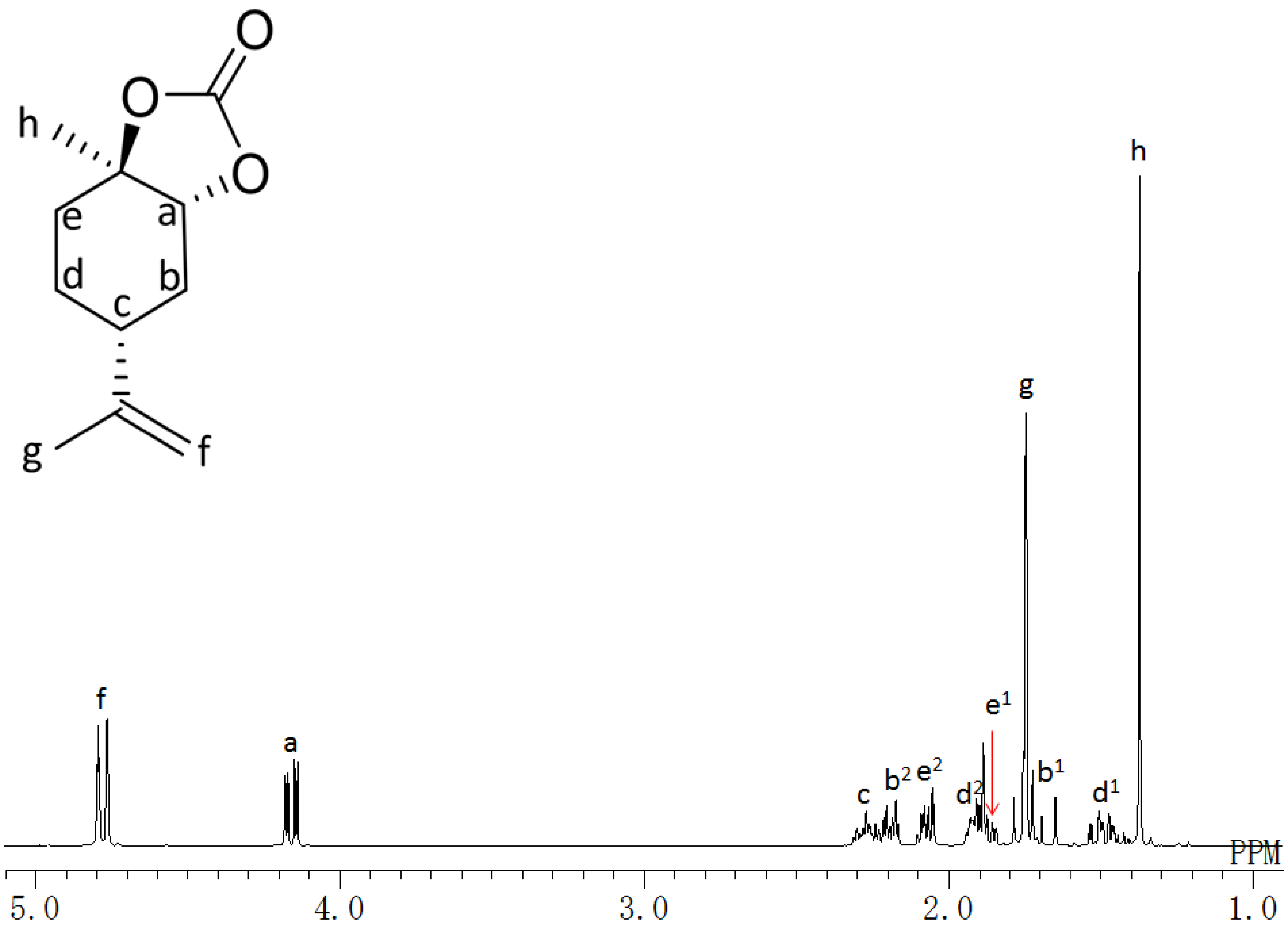

^{1}H NMR spectrum of **1c** in $CDCl_3$.

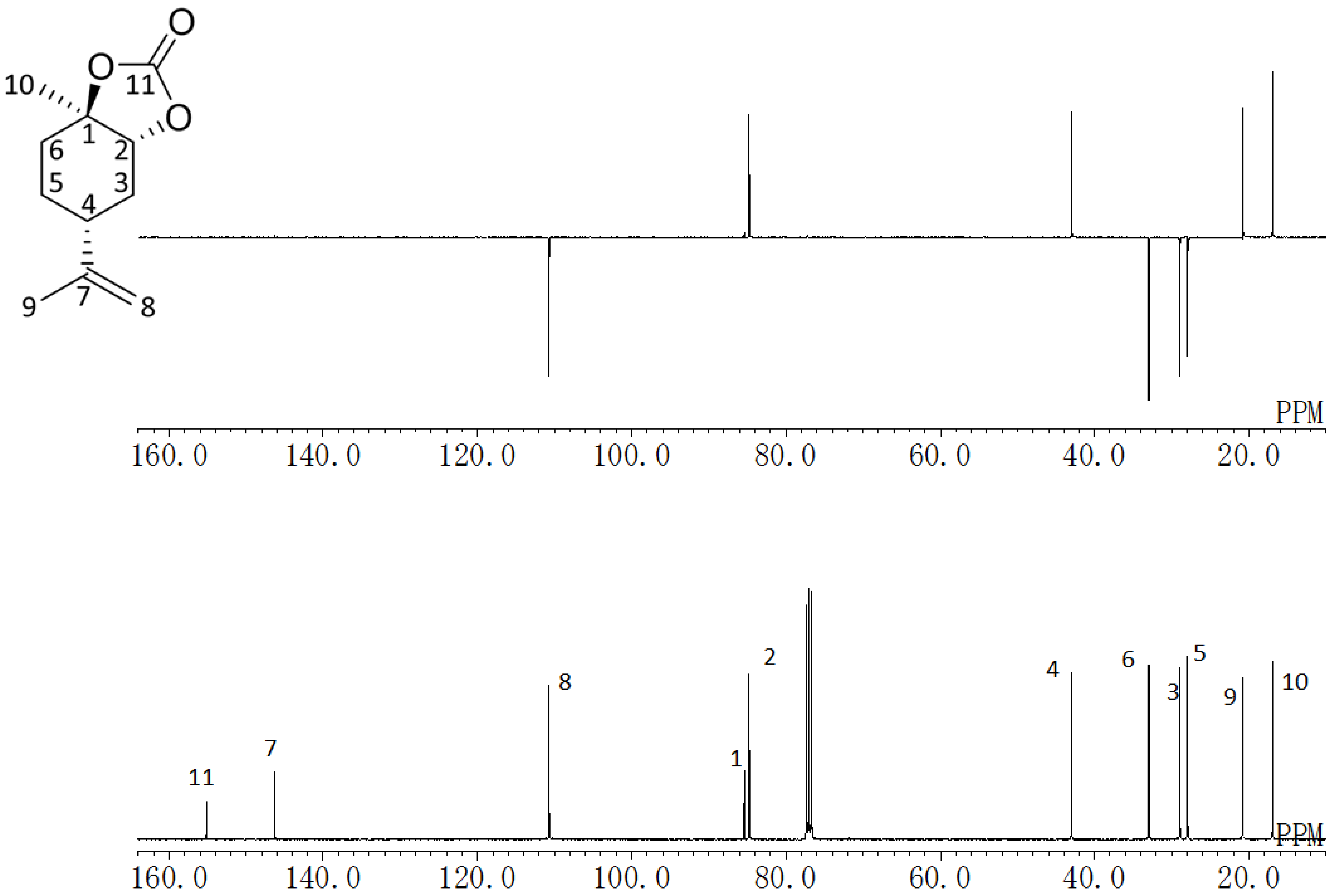

(top) DEPT135 and (bottom) ^{13}C NMR spectra of **1c** in $CDCl_3$.

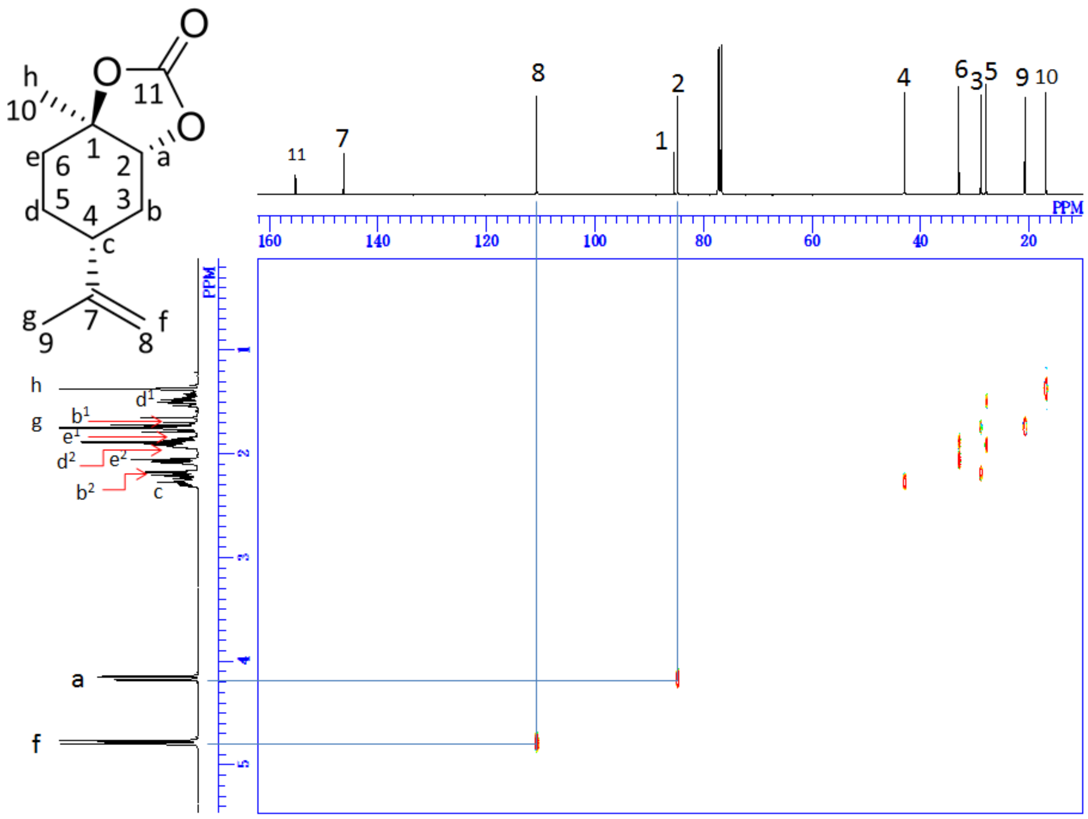

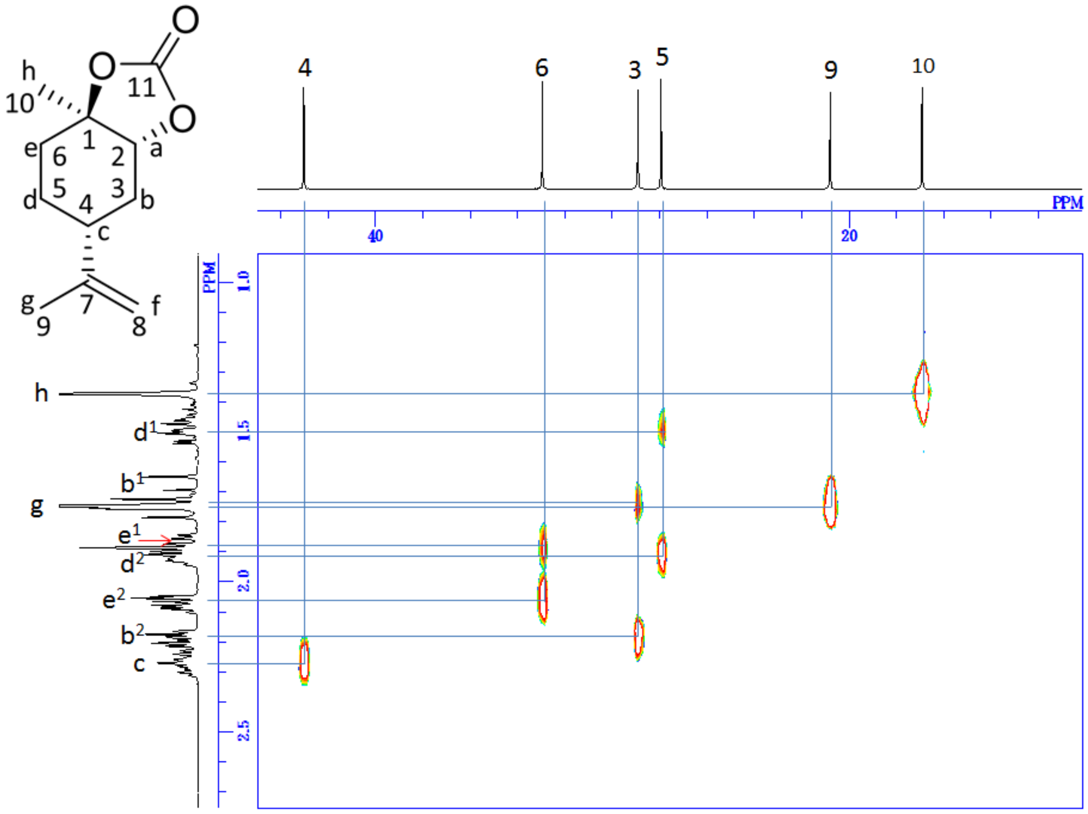

HETCOR spectra of **1c** in $CDCl_3$; (top) full range and (bottom) selected range.

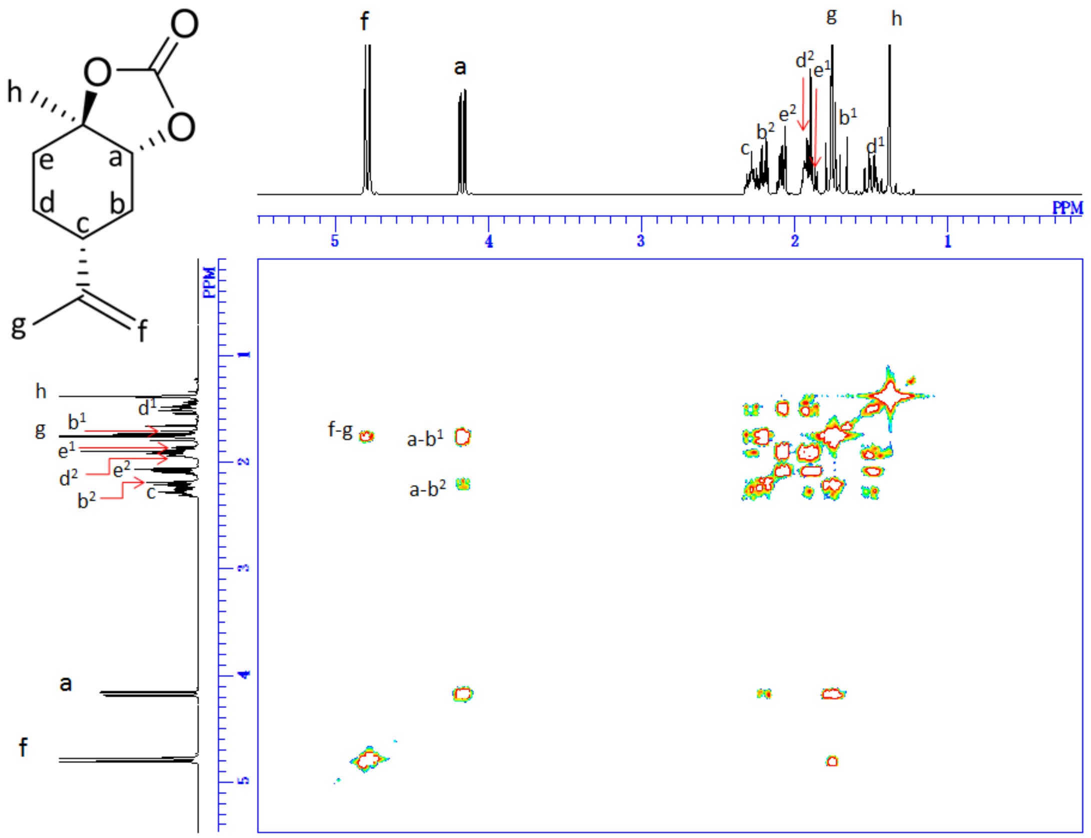

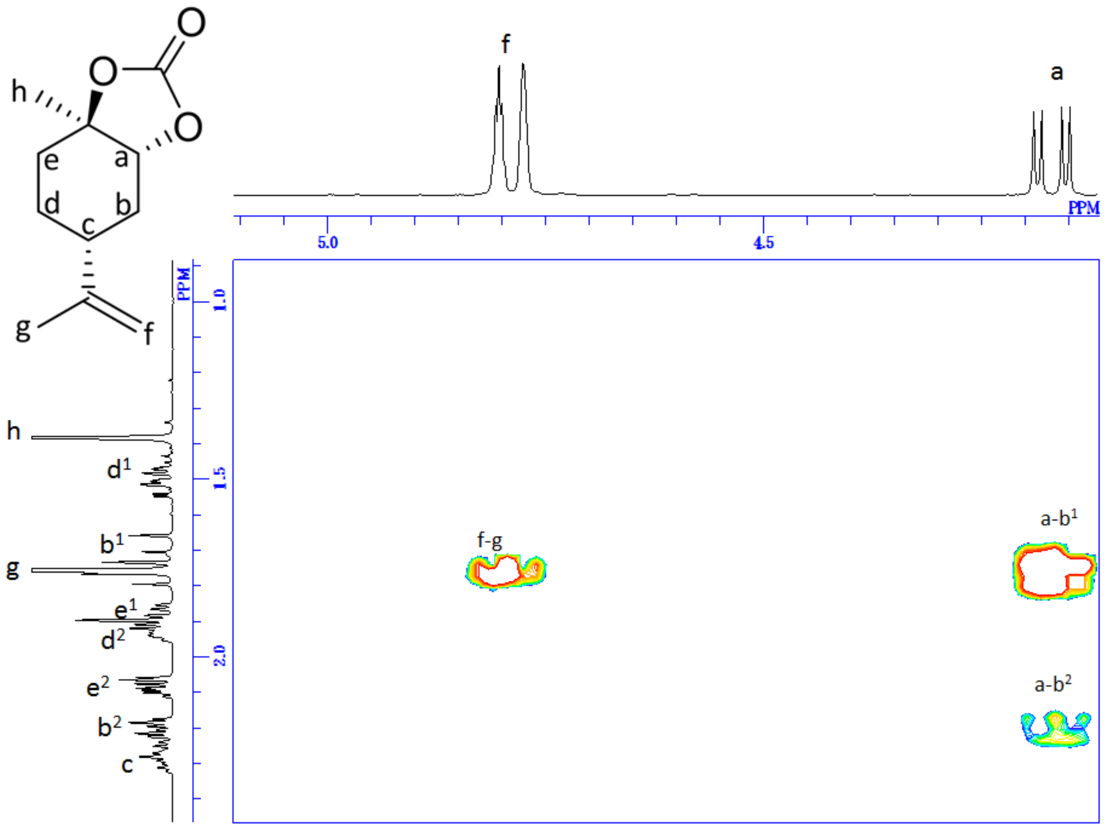

^{1}H-^{1}H COSY spectra of **1c** in $CDCl_3$; (top) full range and (bottom) selected range.

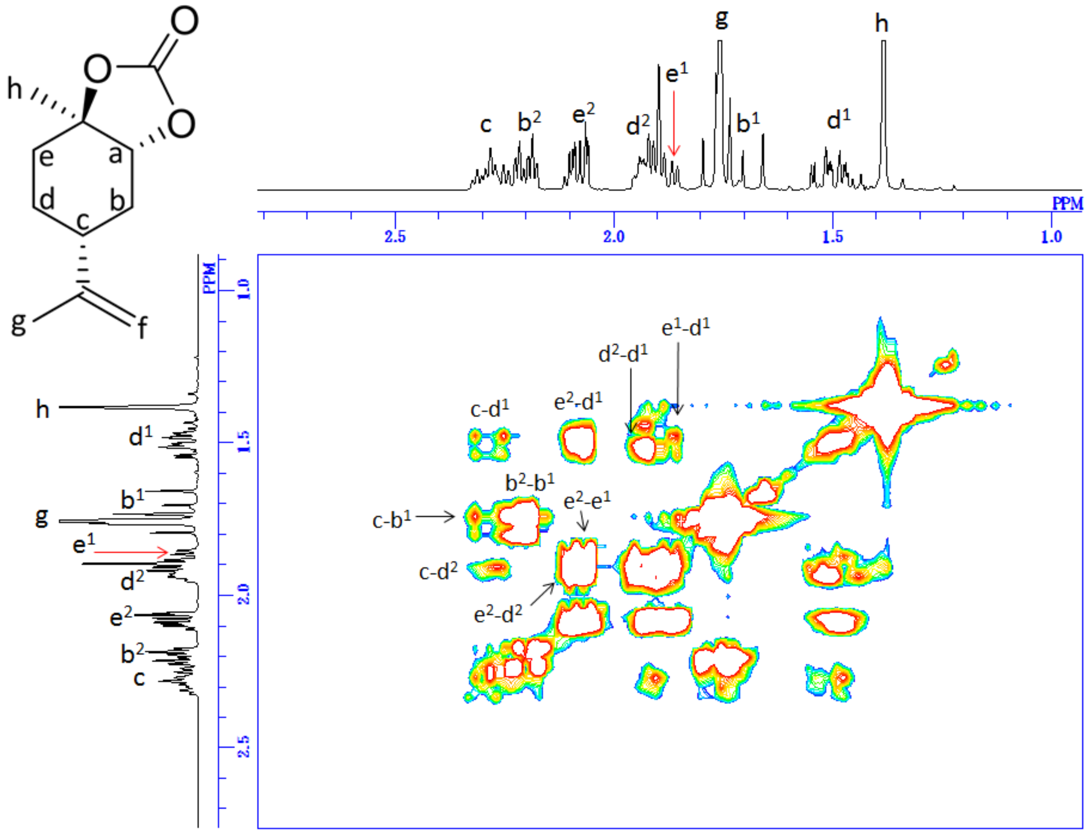

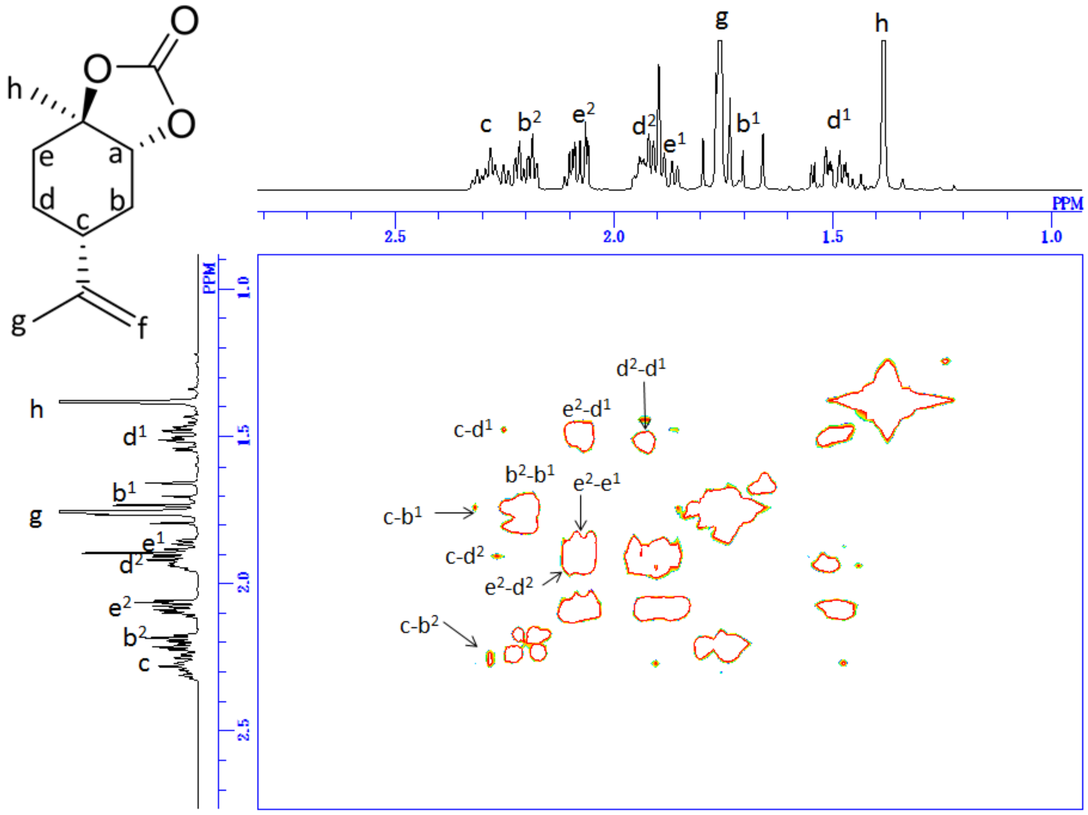

^{1}H-^{1}H COSY spectra of **1c** in $CDCl_3$; selected range with (top) high sensitivity and (bottom) low sensitivity.

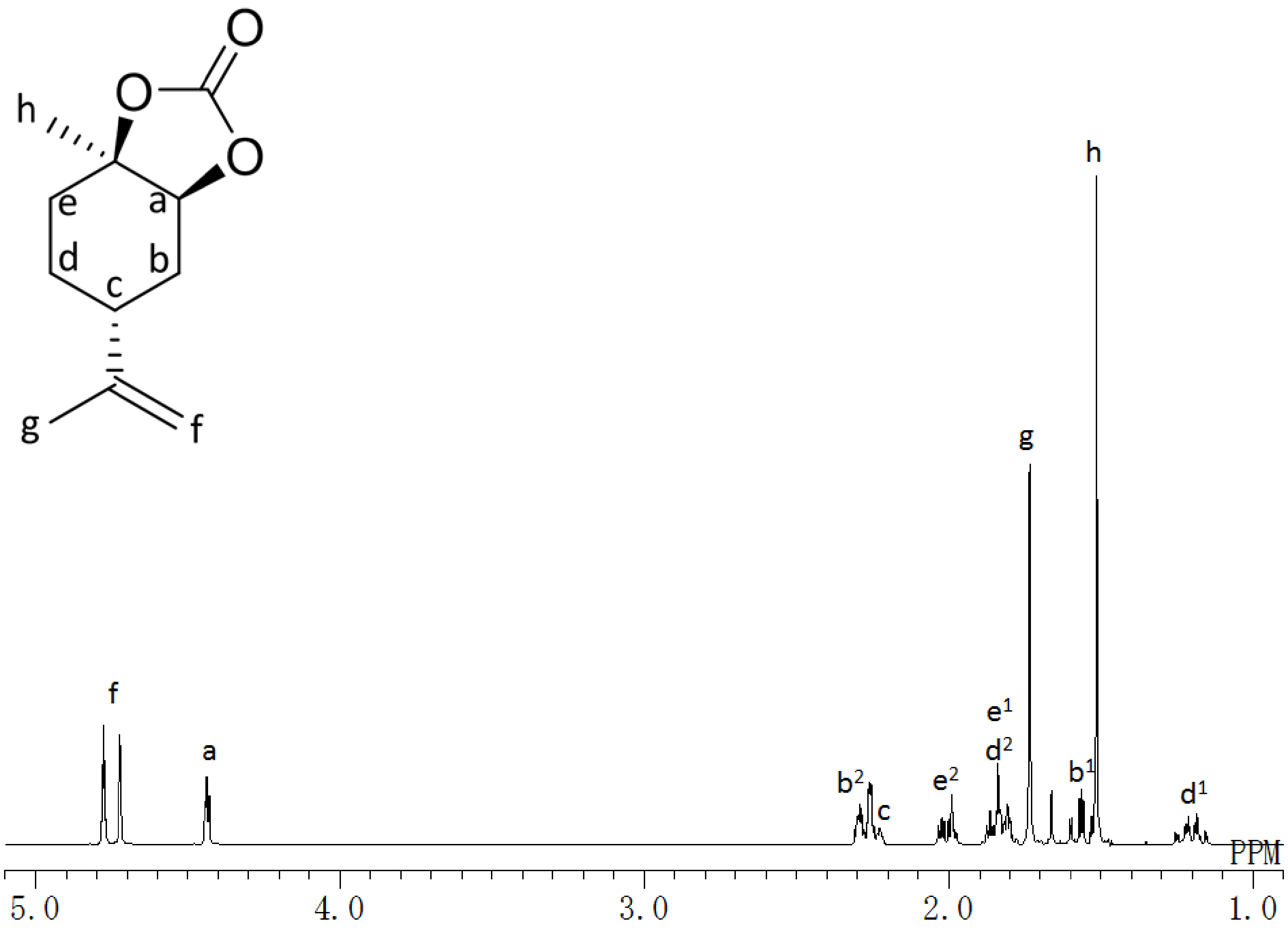

^{1}H NMR spectrum of **1d** in $CDCl_3$.

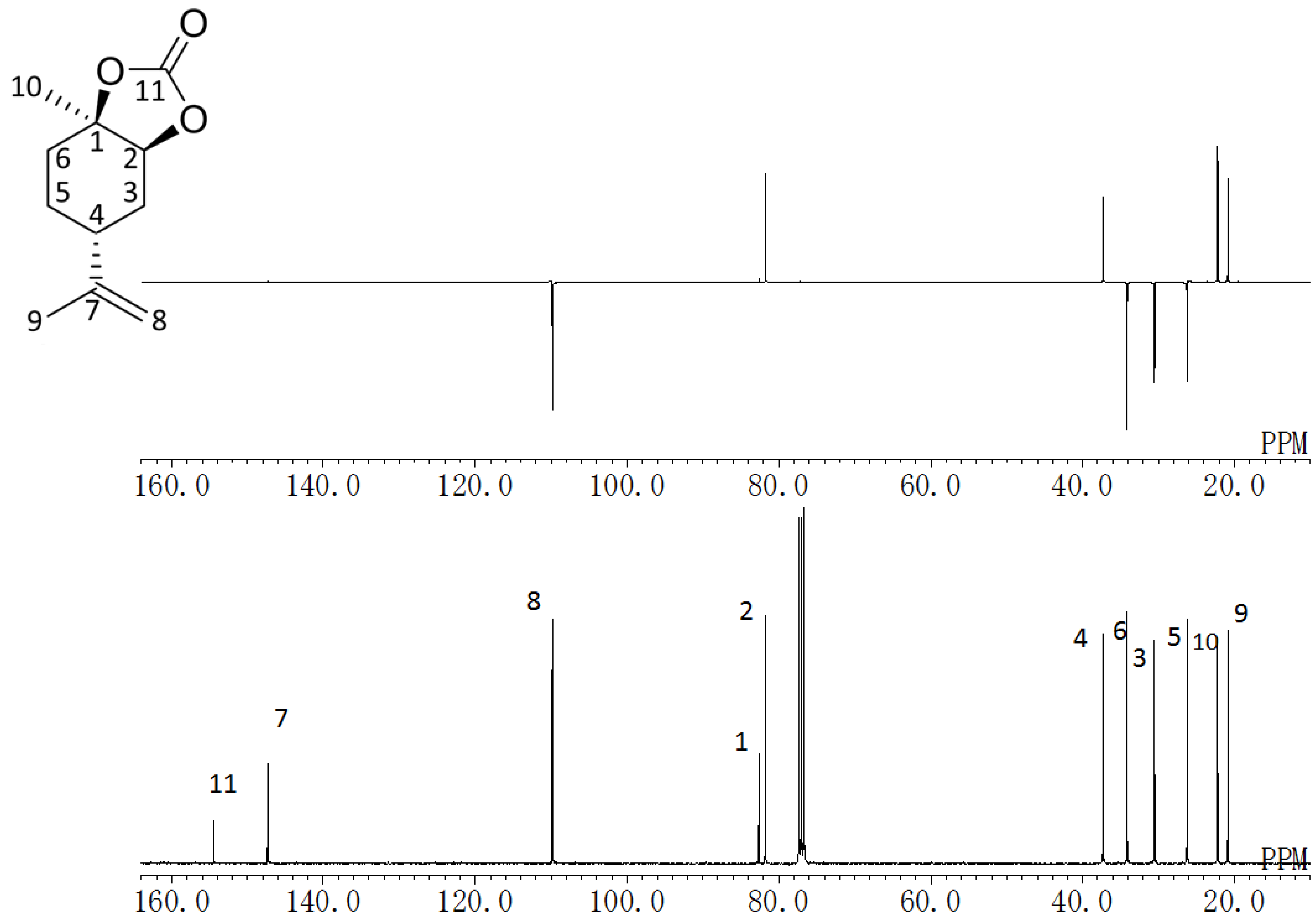

(top) DEPT135 and (bottom) ^{13}C NMR spectra of **1d** in $CDCl_3$.

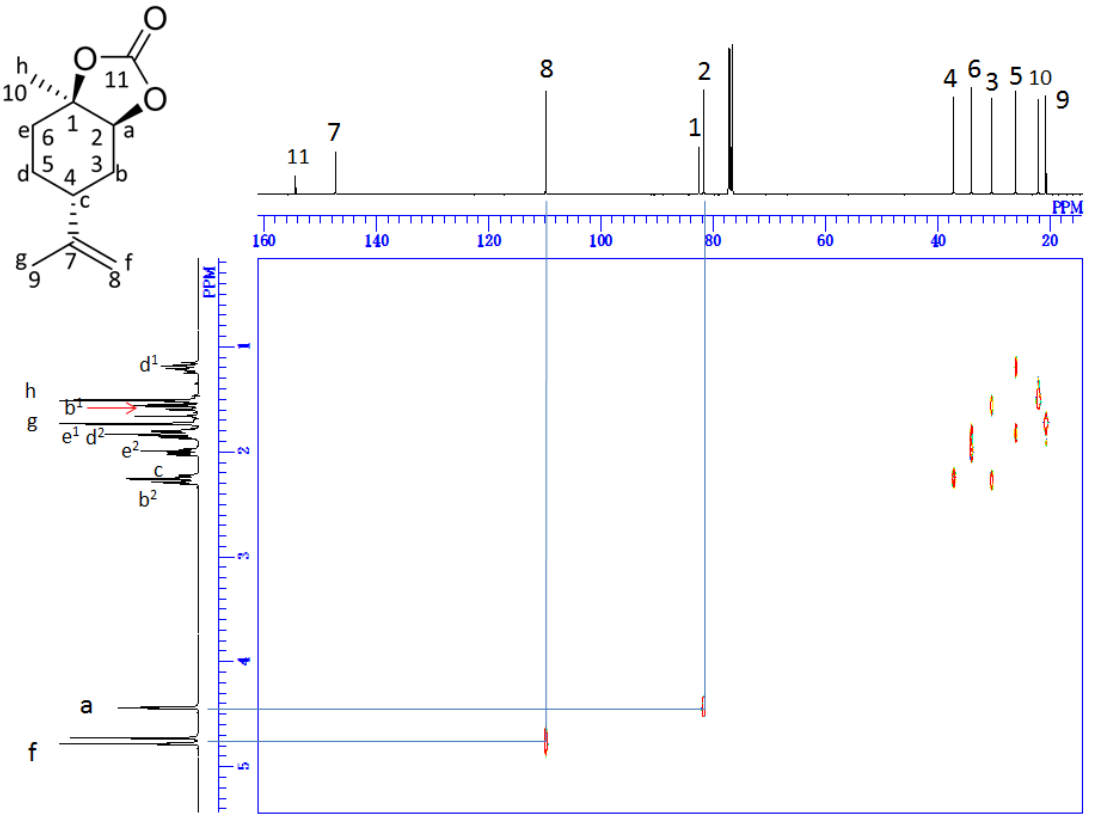

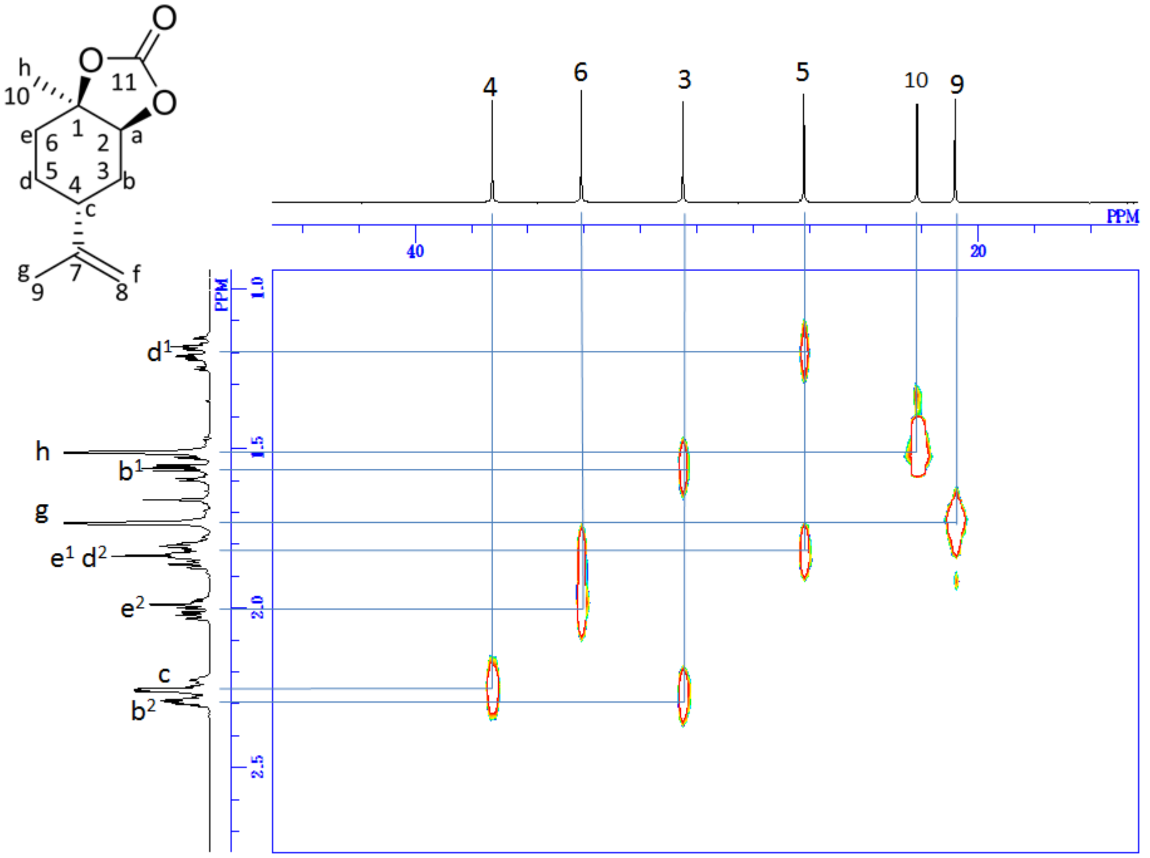

HETCOR spectra of **1d** in $CDCl_3$; (top) full range and (bottom) selected range.

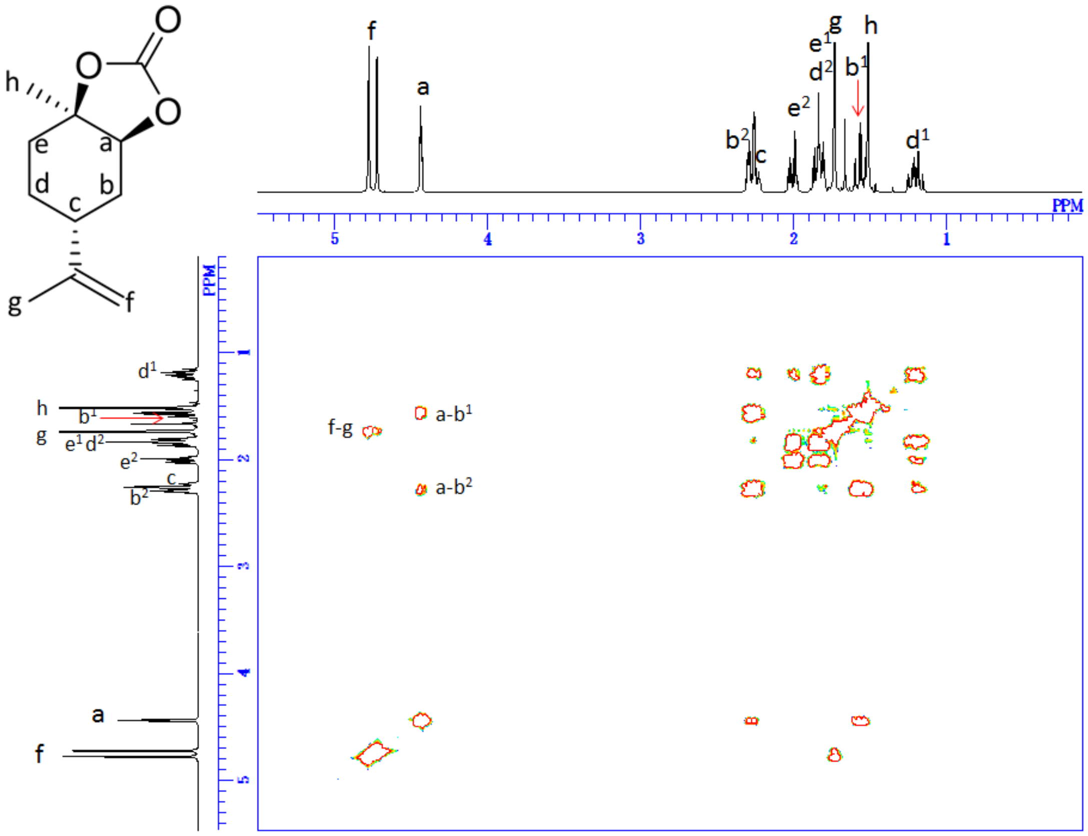

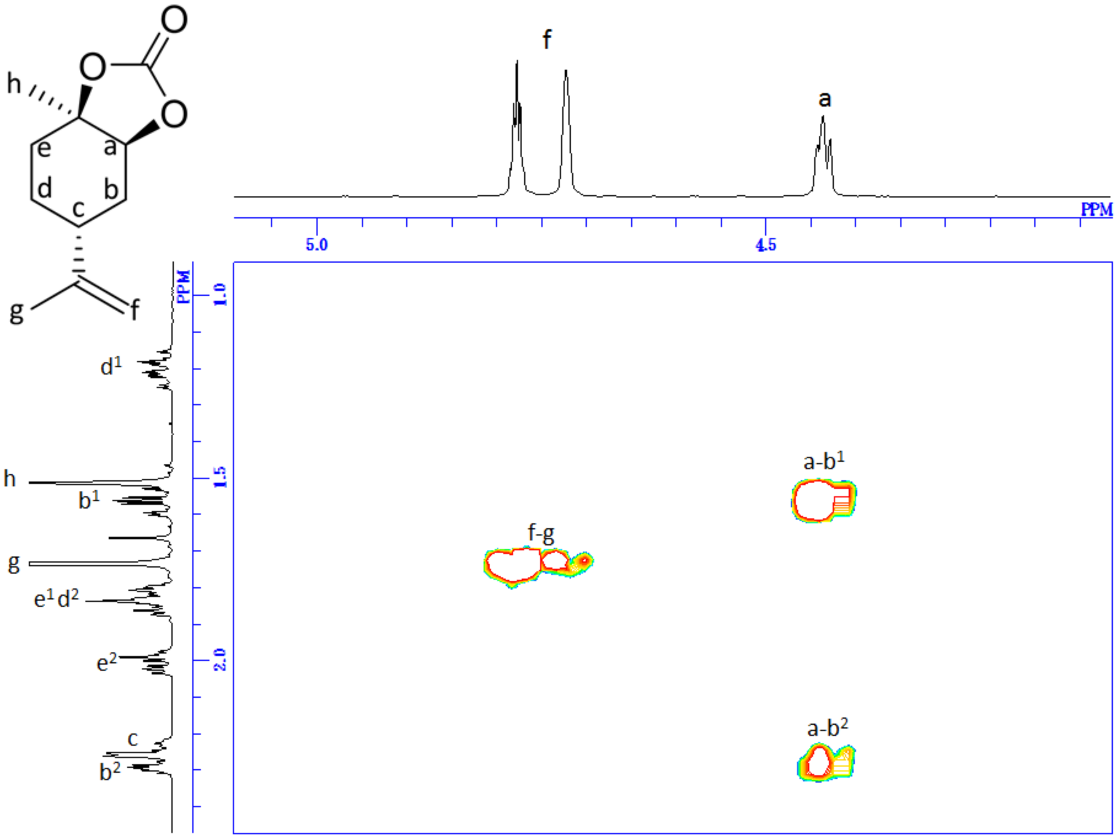

^1H-^1H COSY spectra of **1d** in $CDCl_3$; (top) full range and (bottom) selected range.

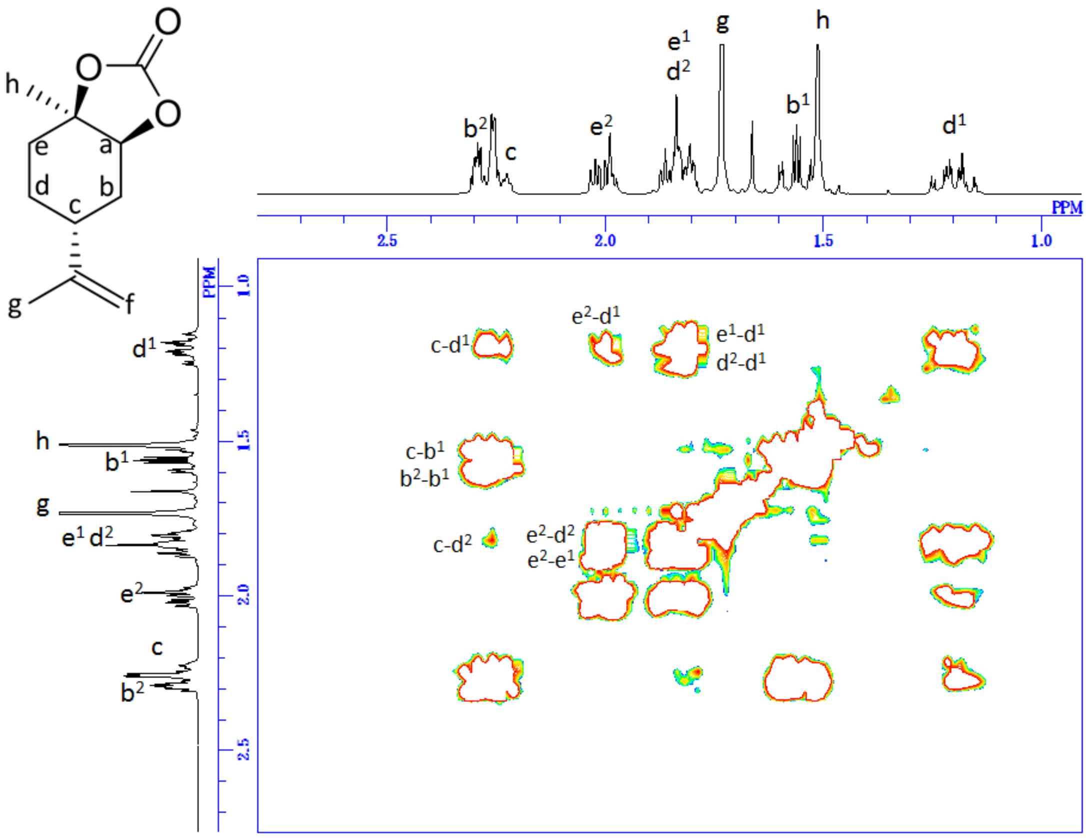

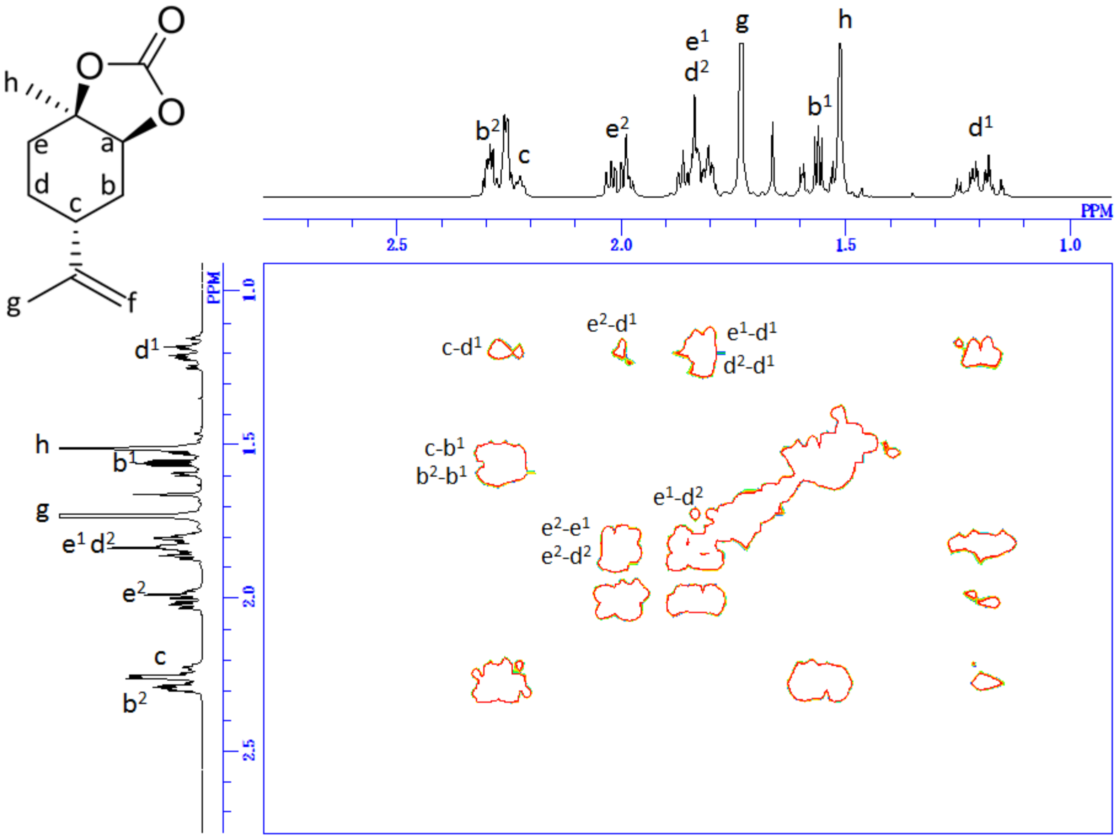

^{1}H-^{1}H COSY spectra of **1d** in $CDCl_3$; selected range with (top) high sensitivity and (bottom) low sensitivity.

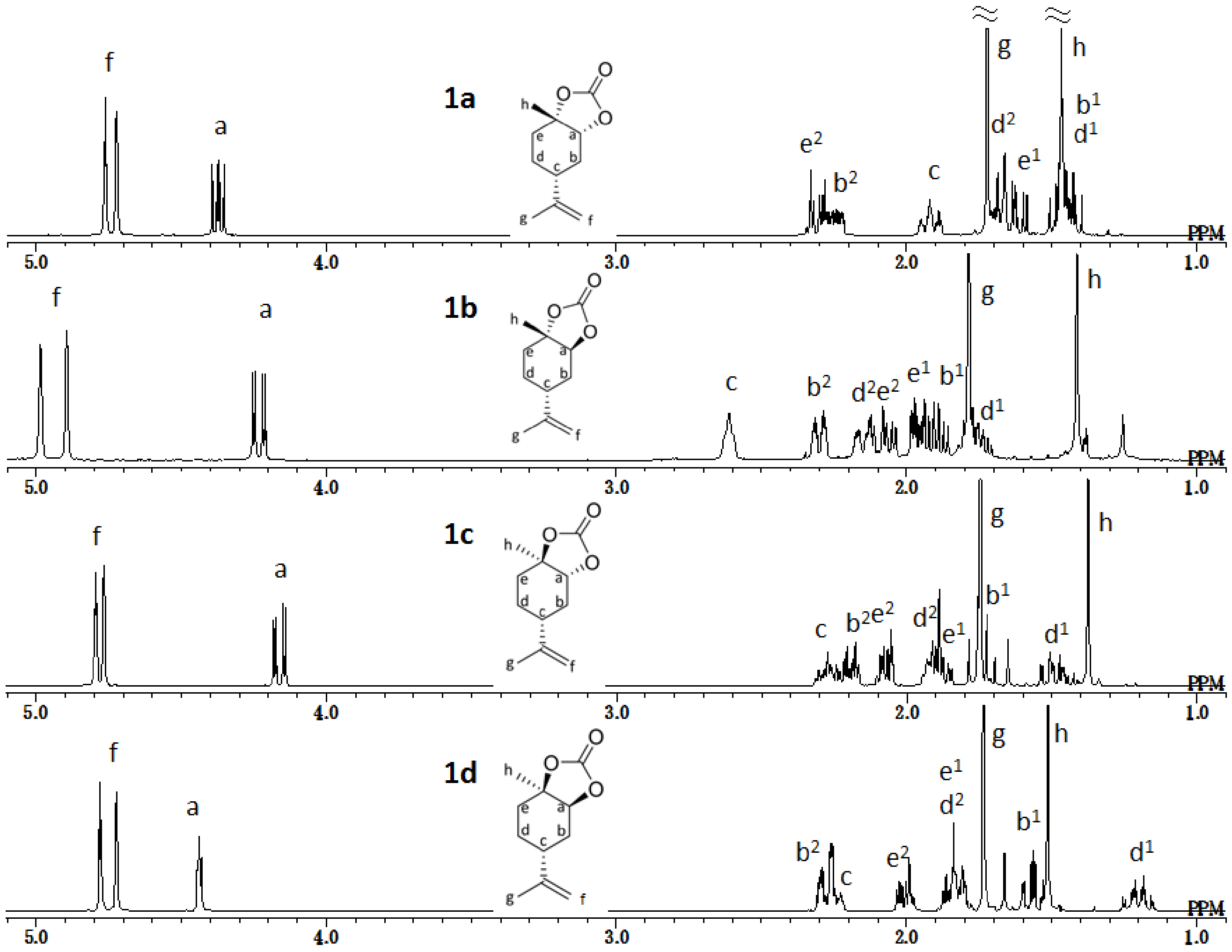

^{1}H NMR spectra of four LM5CCs (**1a**–**1d**) in full scales in $CDCl_3$.

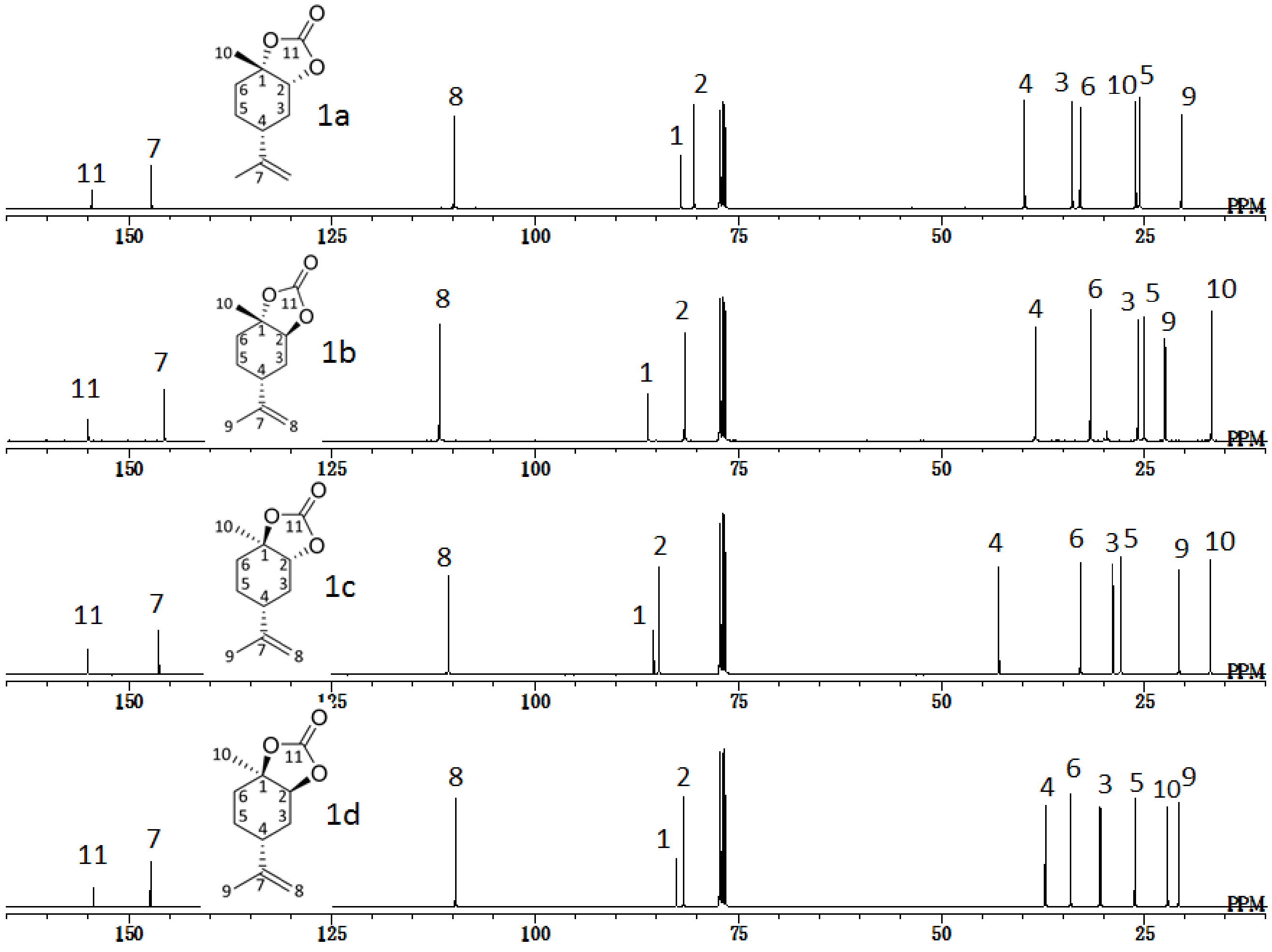

^{13}C NMR spectra of four LM5CCs (**1a**–**1d**) in full scales in $CDCl_3$.

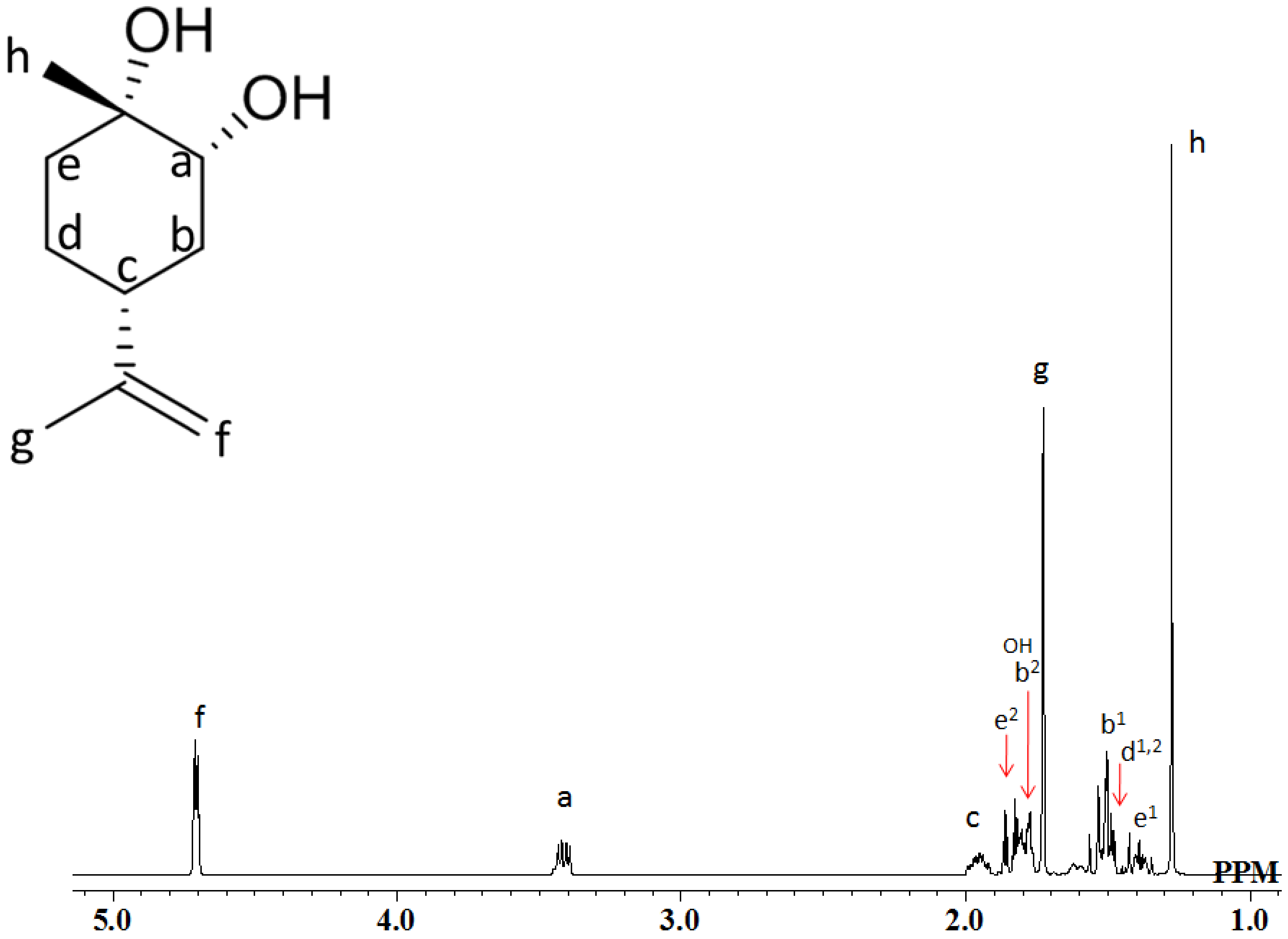

^{1}H NMR spectrum of **2a** in $CDCl_3$.

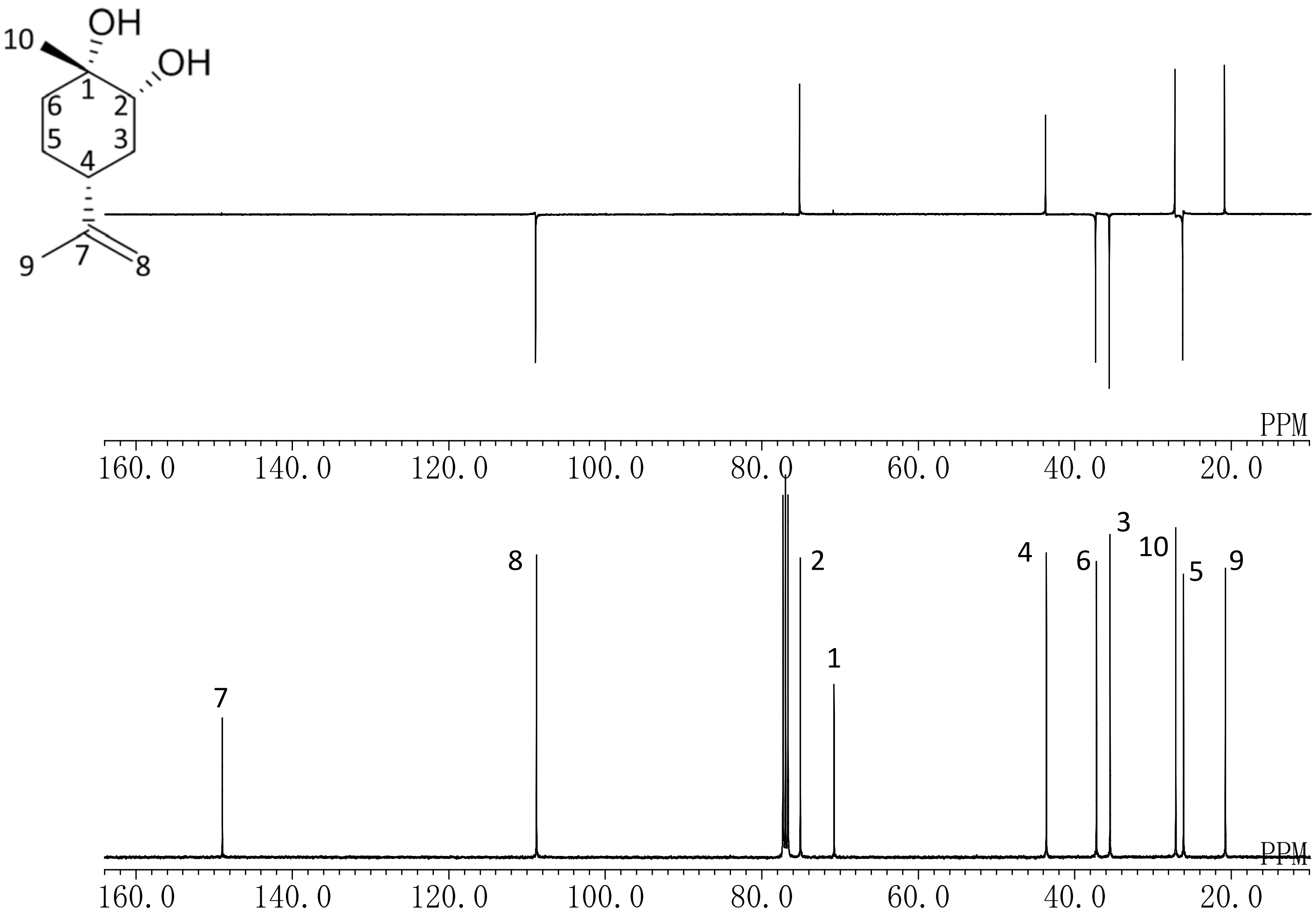

(top) DEPT135 and (bottom) ^{13}C NMR spectra of **2a** in $CDCl_3$

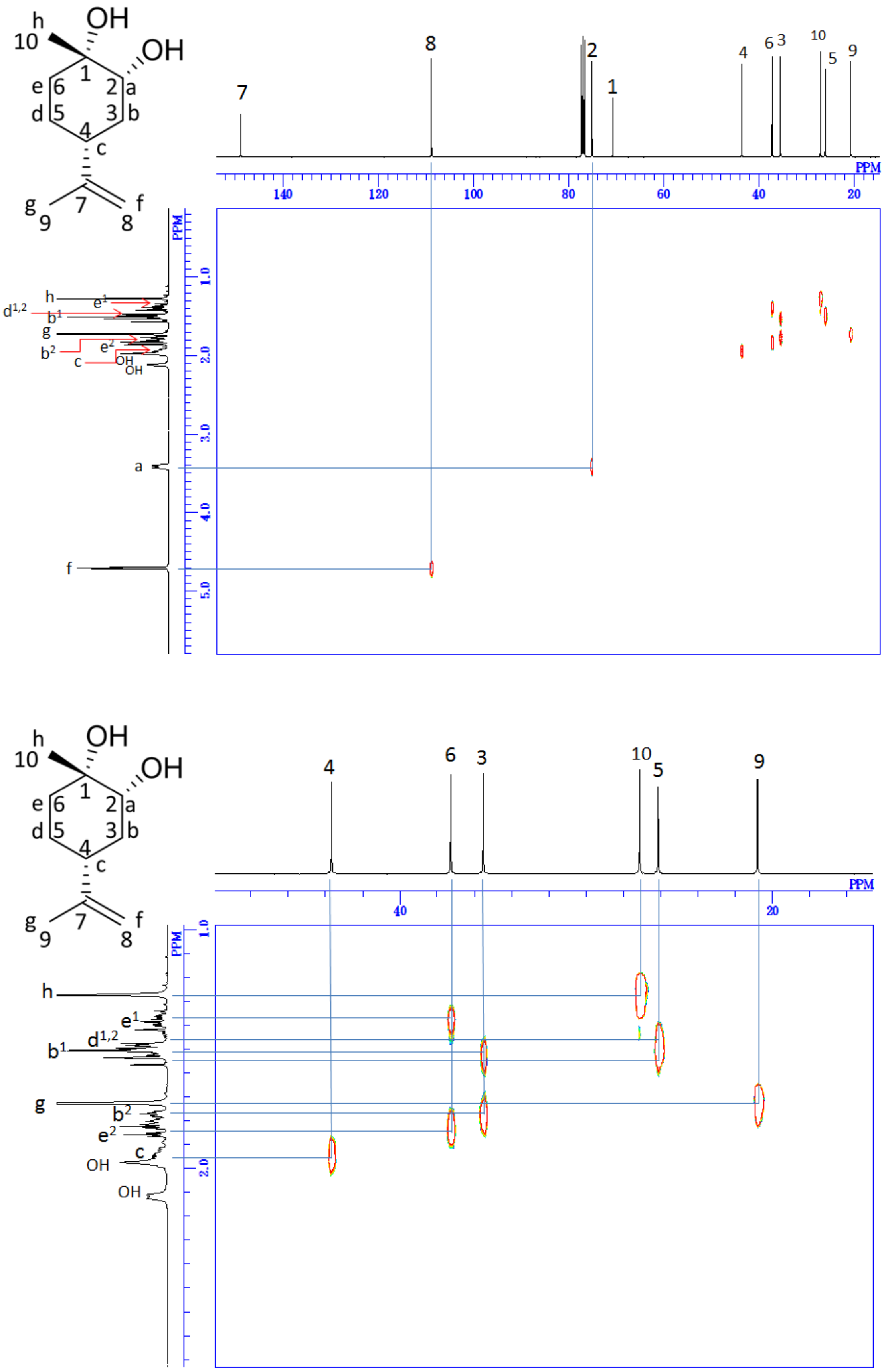

HETCOR spectra of **2a** in $CDCl_3$; (top) full range and (bottom) selected range.

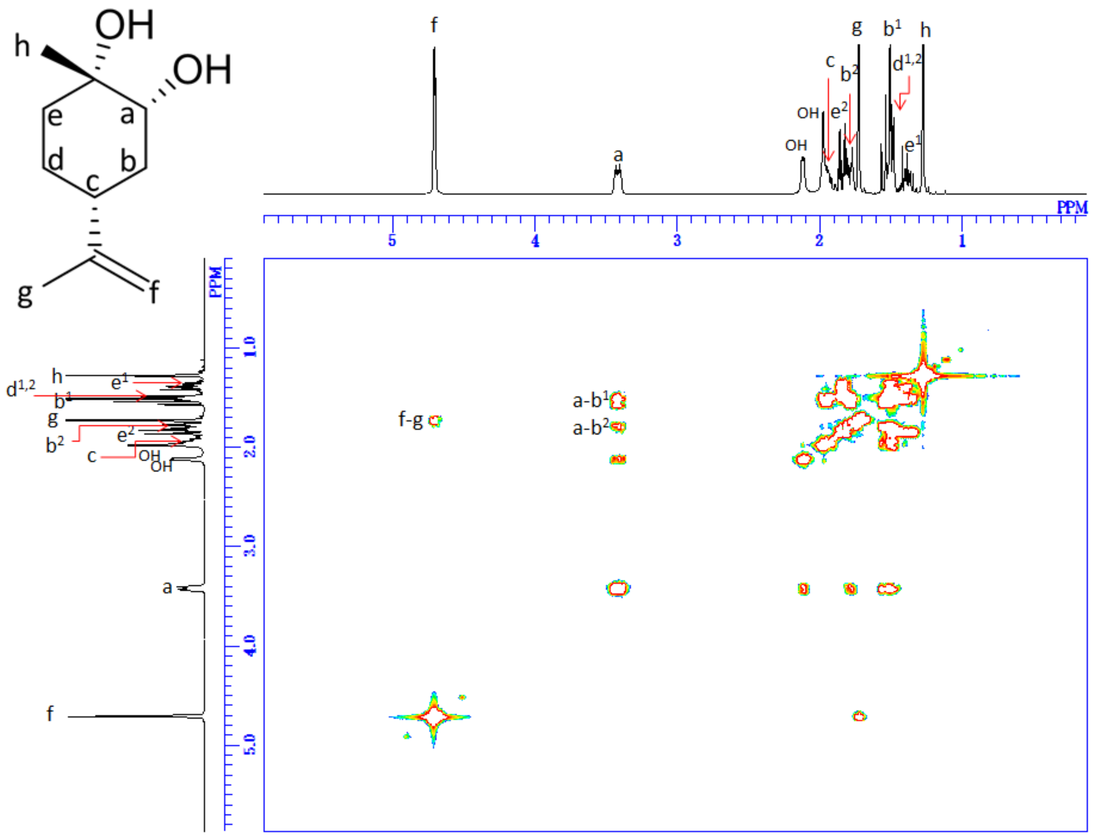

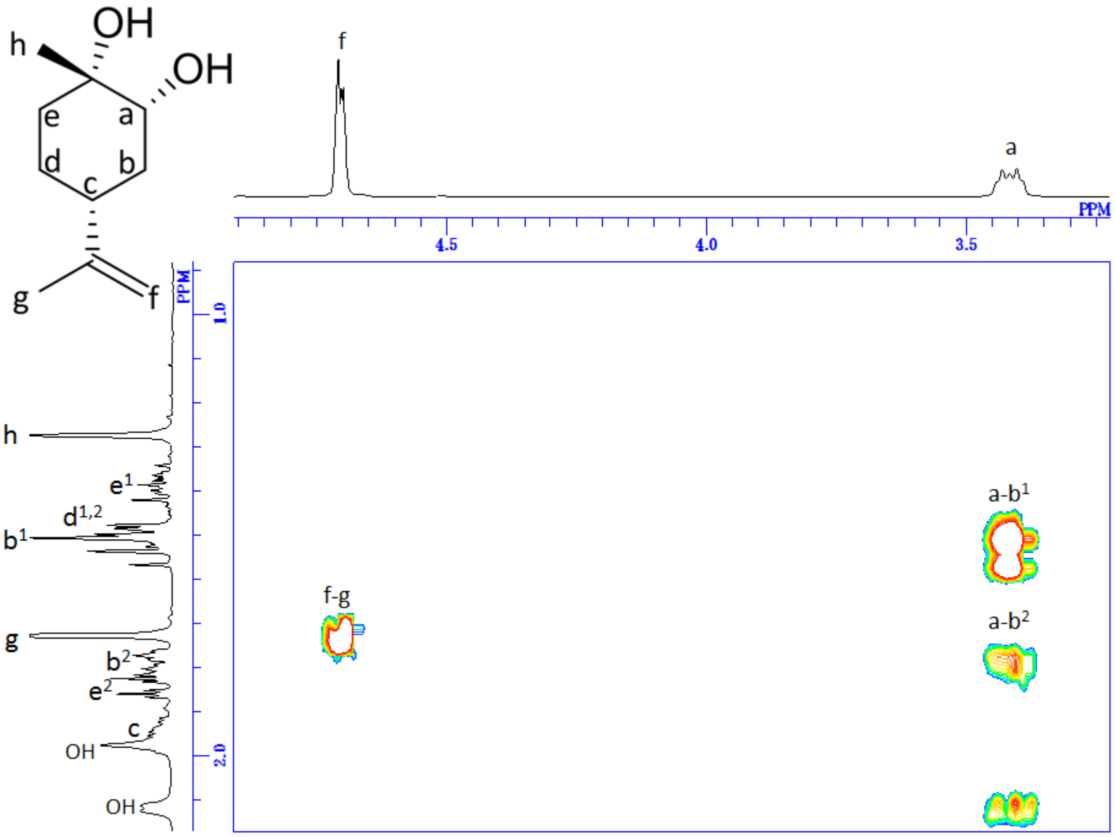

^{1}H-^{1}H COSY spectra of **2a** in $CDCl_3$; (top) full range and (bottom) selected range.

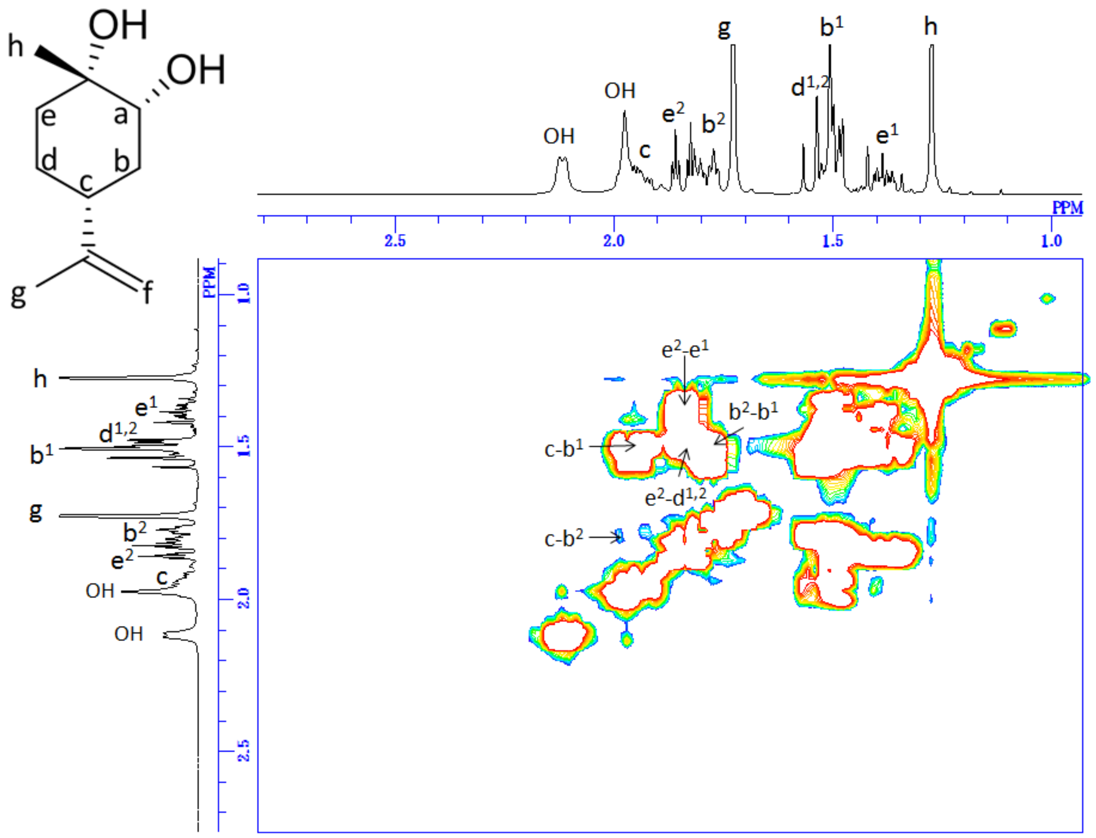

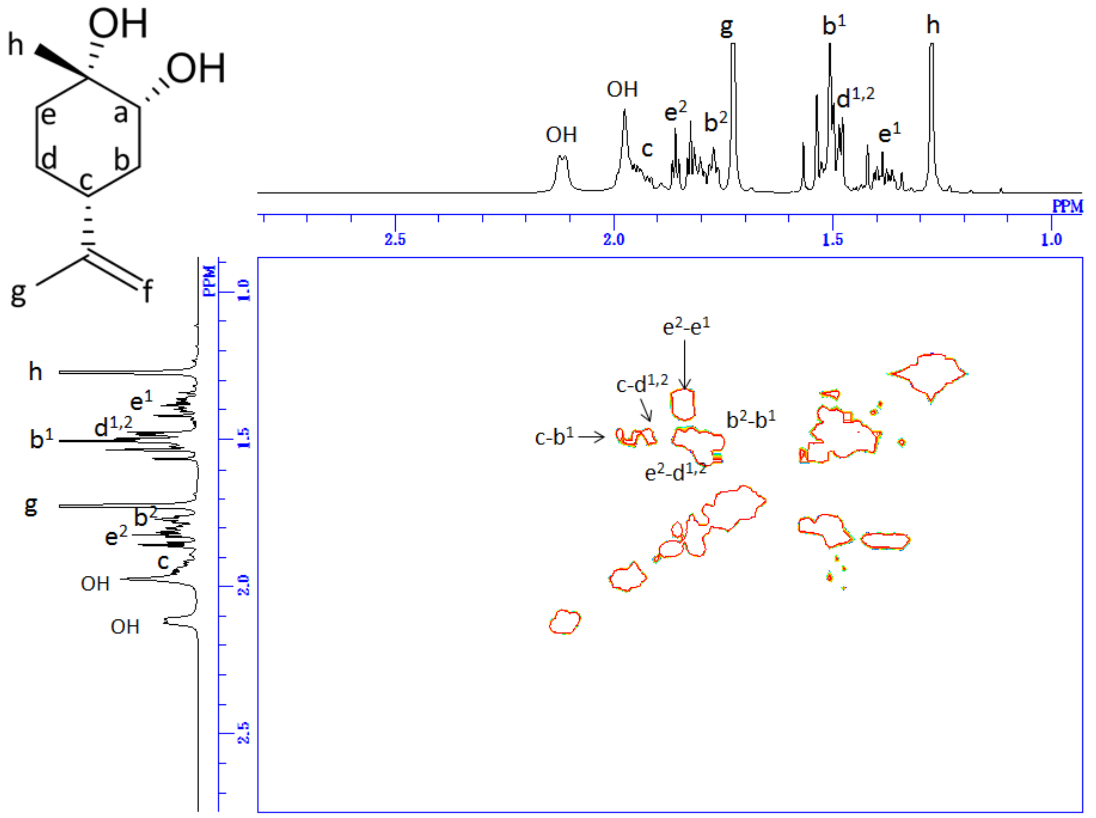

^{1}H-^{1}H COSY spectra of **2a** in $CDCl_3$; selected range with (top) high sensitivity and (bottom) low sensitivity.

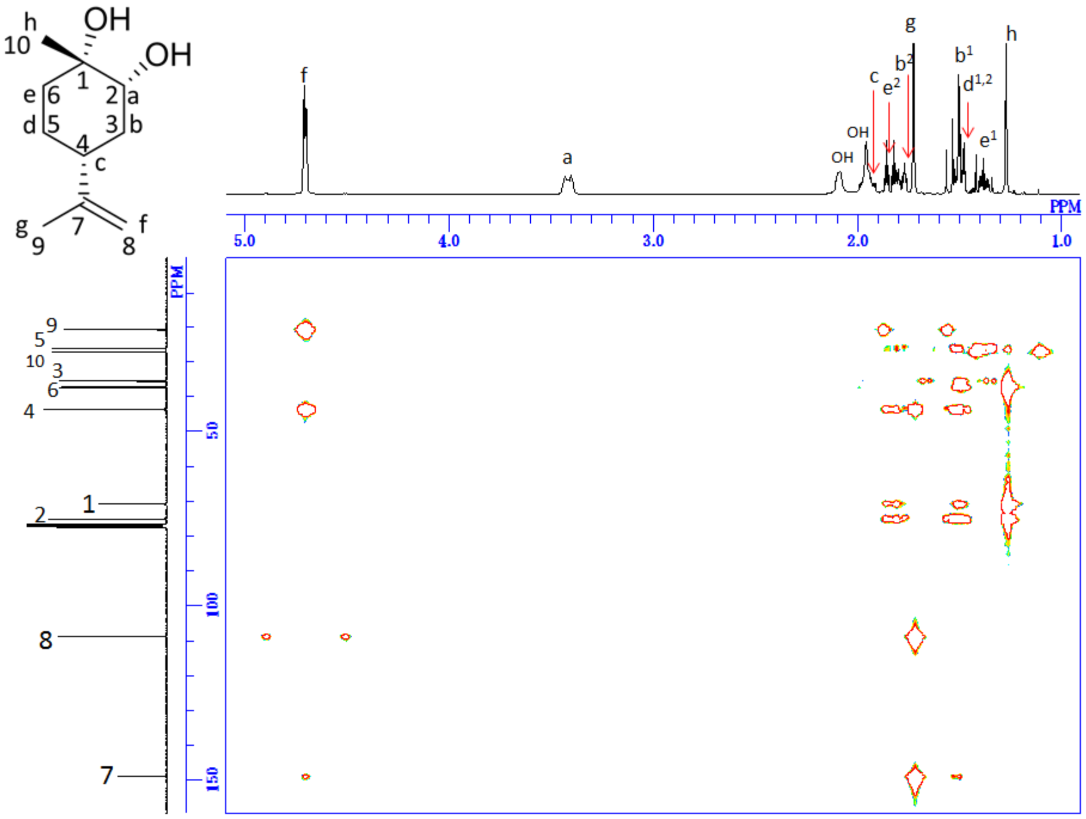

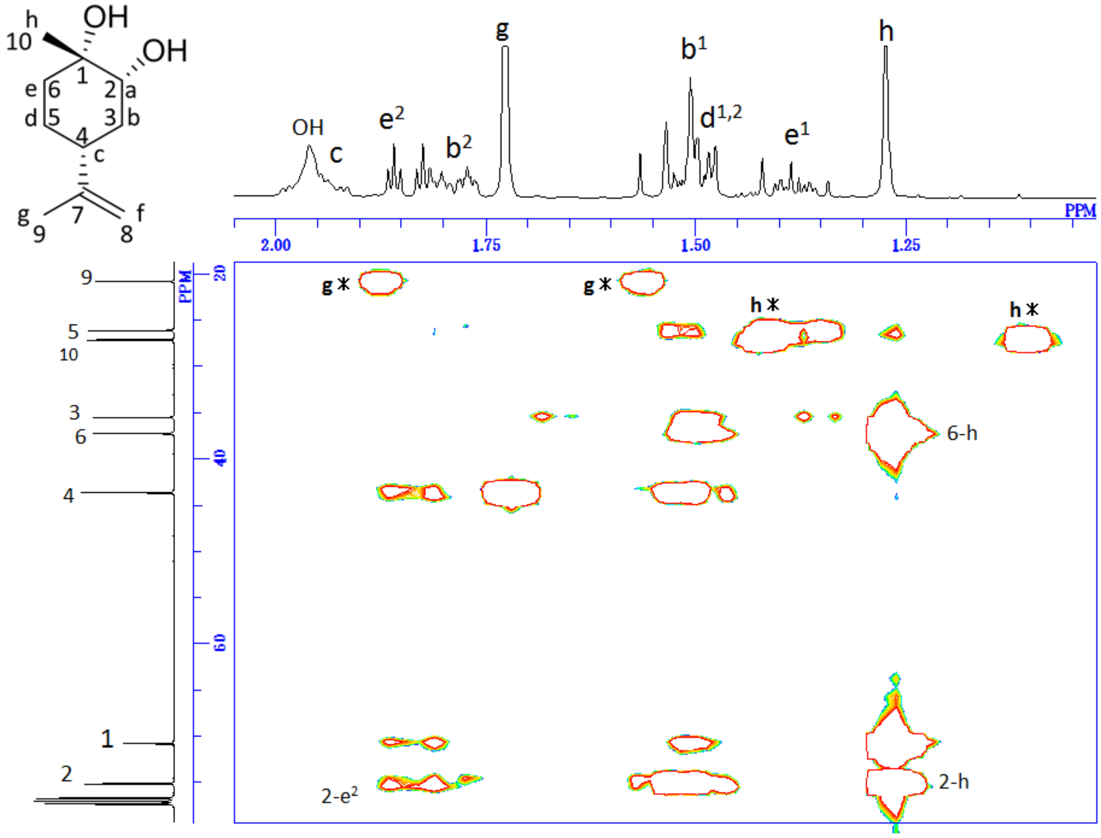

HMBC spectra of **2a** in $CDCl_3$; (top) full range and (bottom) selected range.
The marks (g* and h*) represent for side-band peaks.

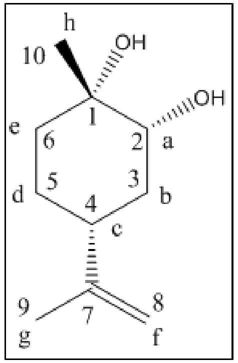

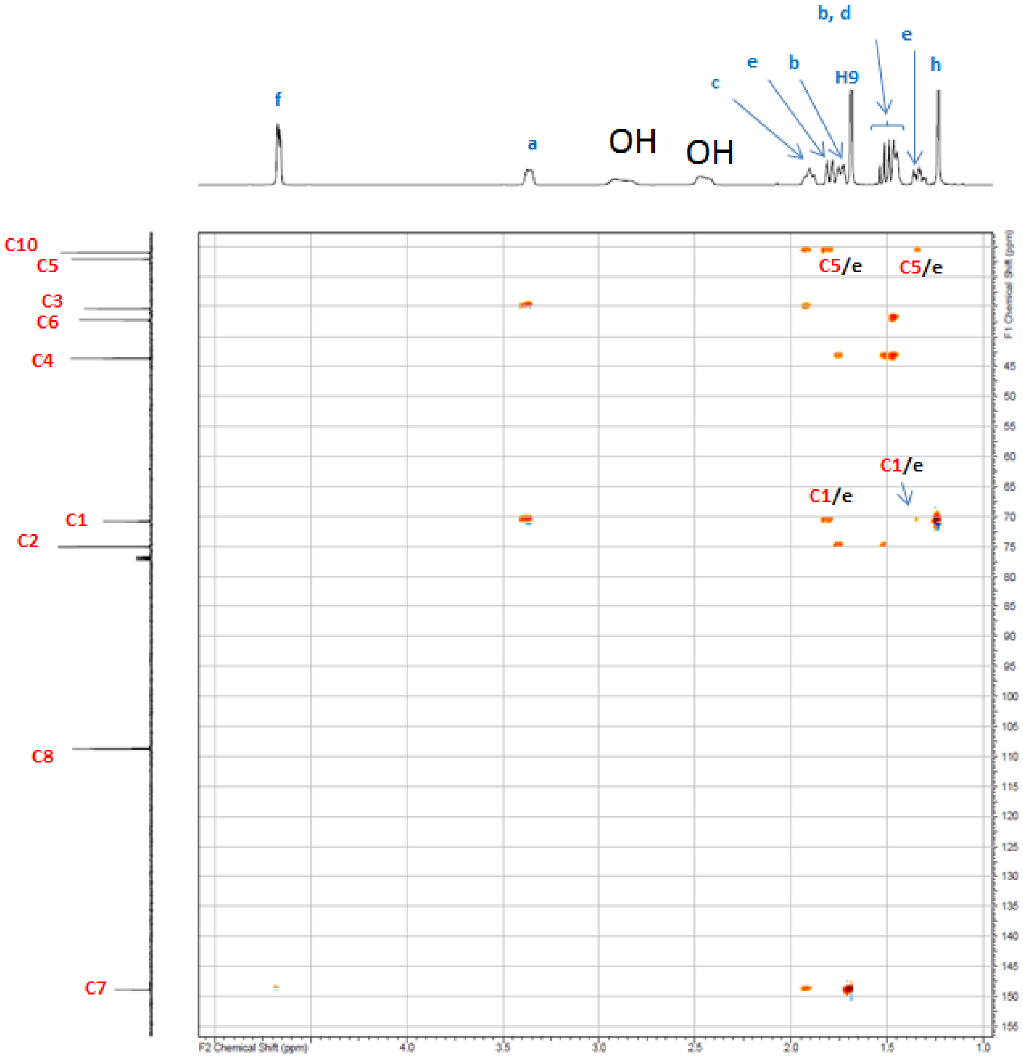

1,1-ADEQUATE spectrum of **2a** in $CDCl_3$.

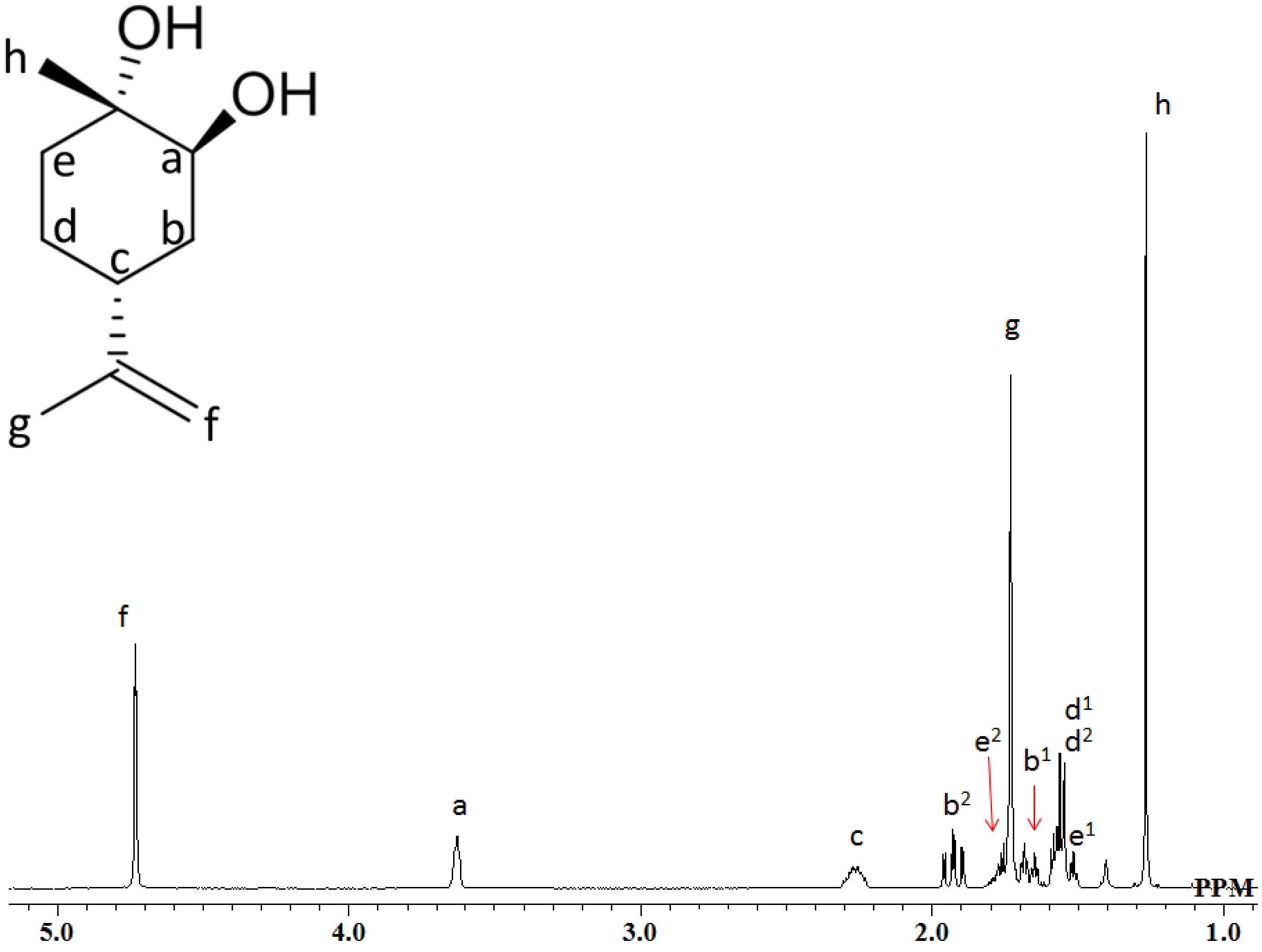

^{1}H NMR spectrum of **2b** in $CDCl_3$.

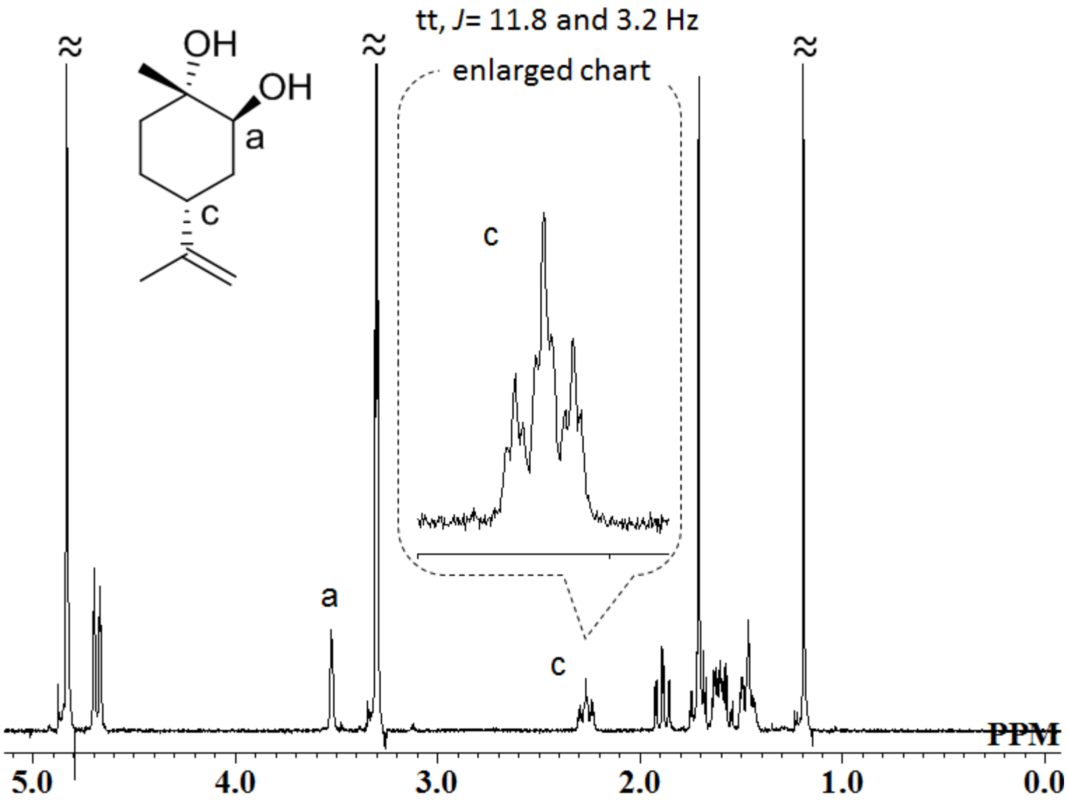

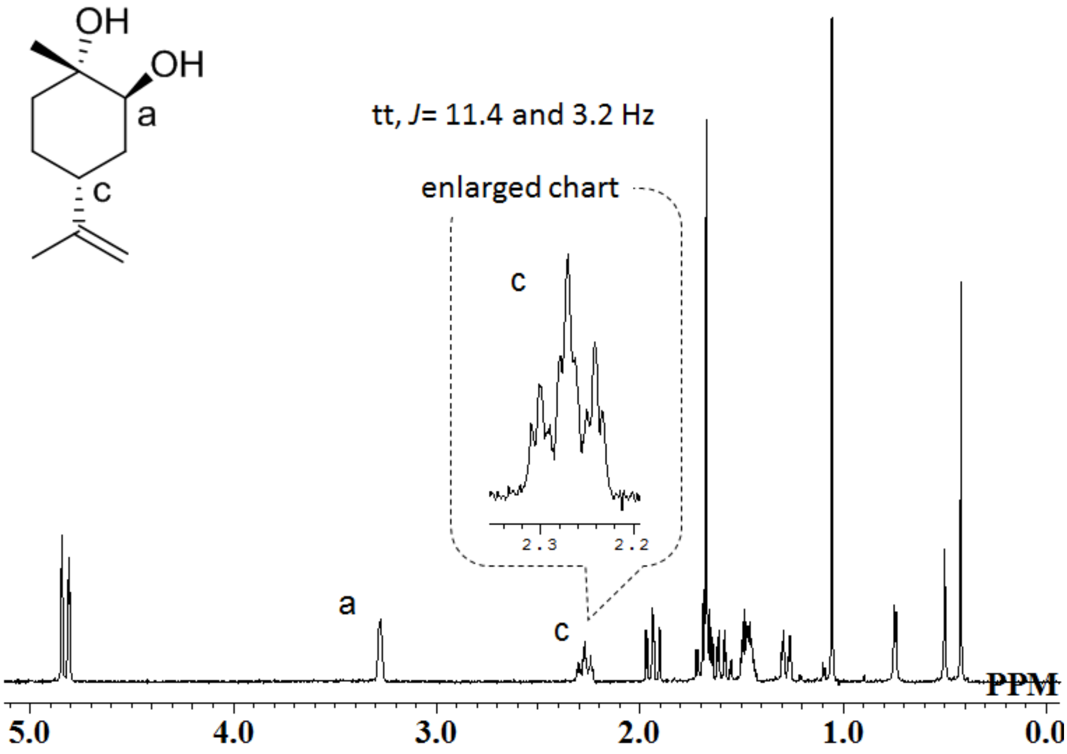

^{1}H NMR spectra of **2b** (top) in CD_3OD and (bottom) in benzene-d_6

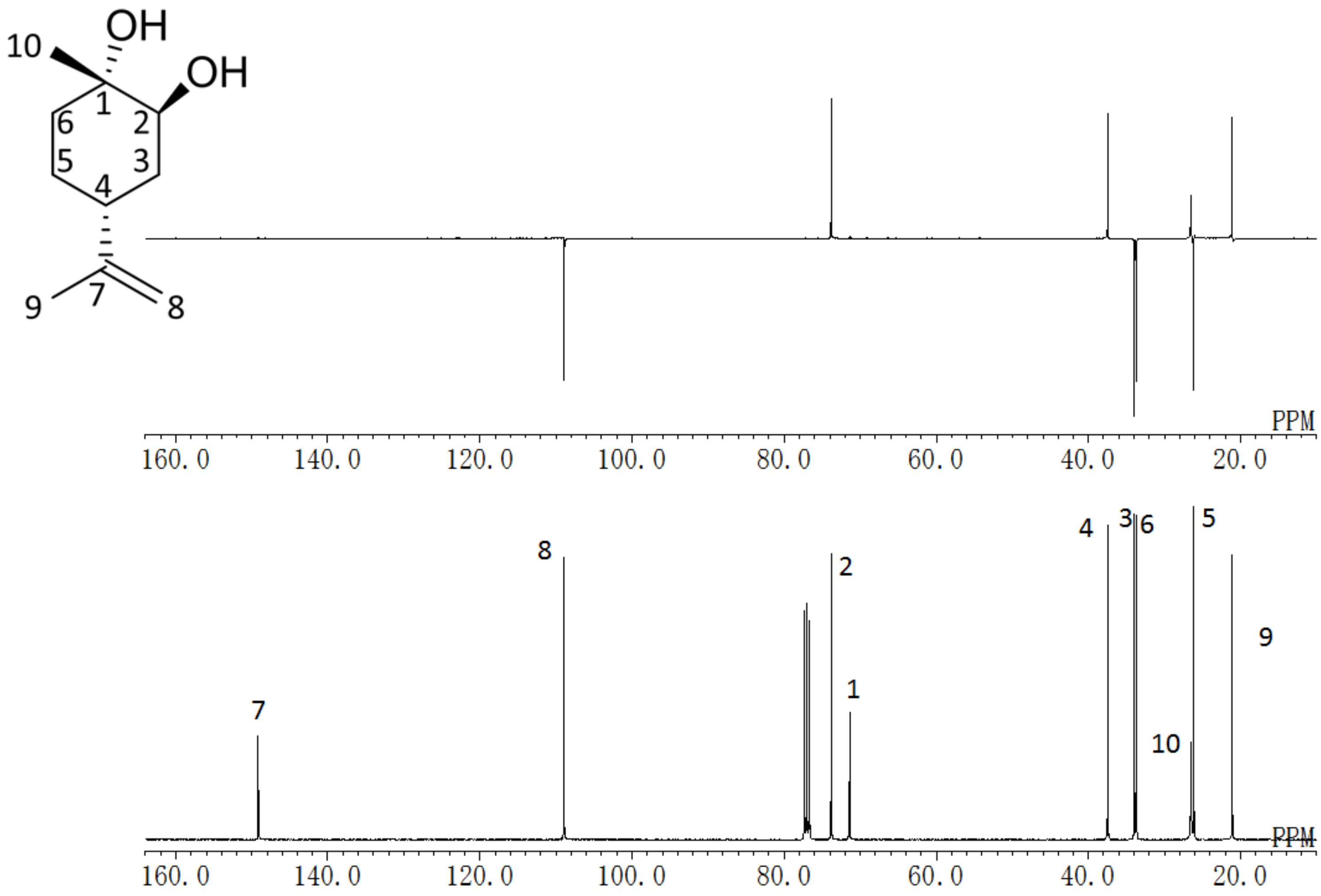

(top) DEPT135 and (bottom) ^{13}C NMR spectra of **2b** in $CDCl_3$.

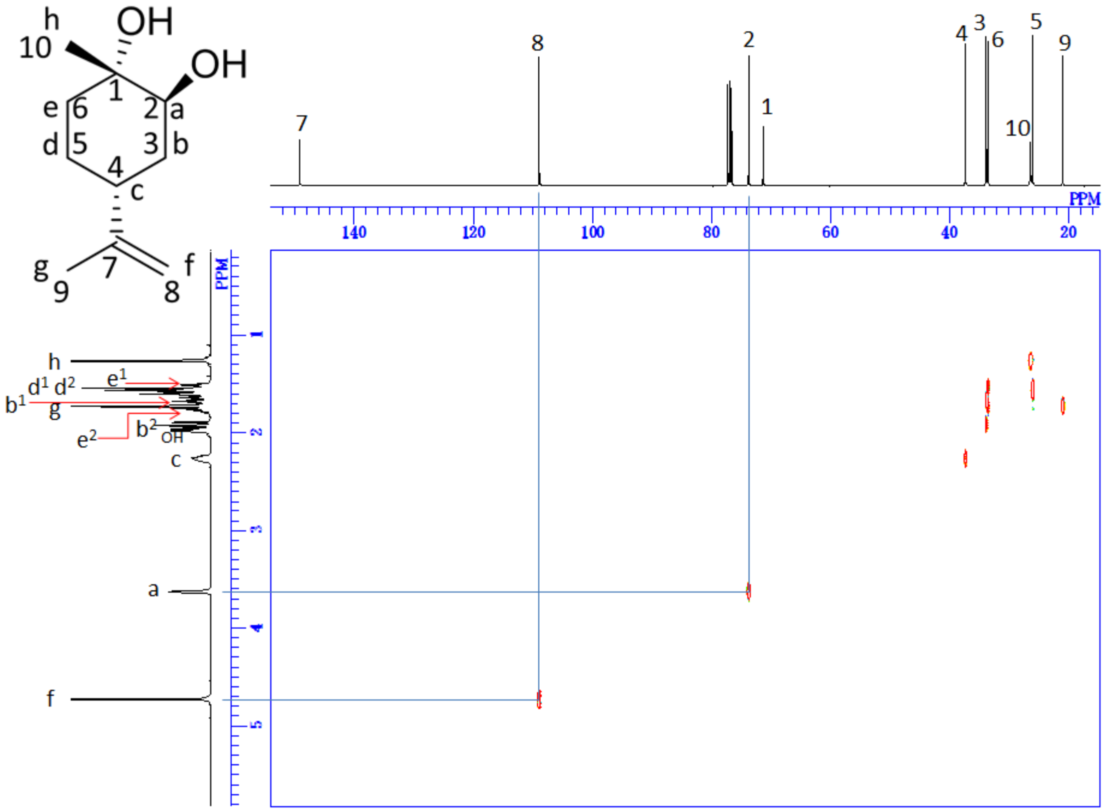

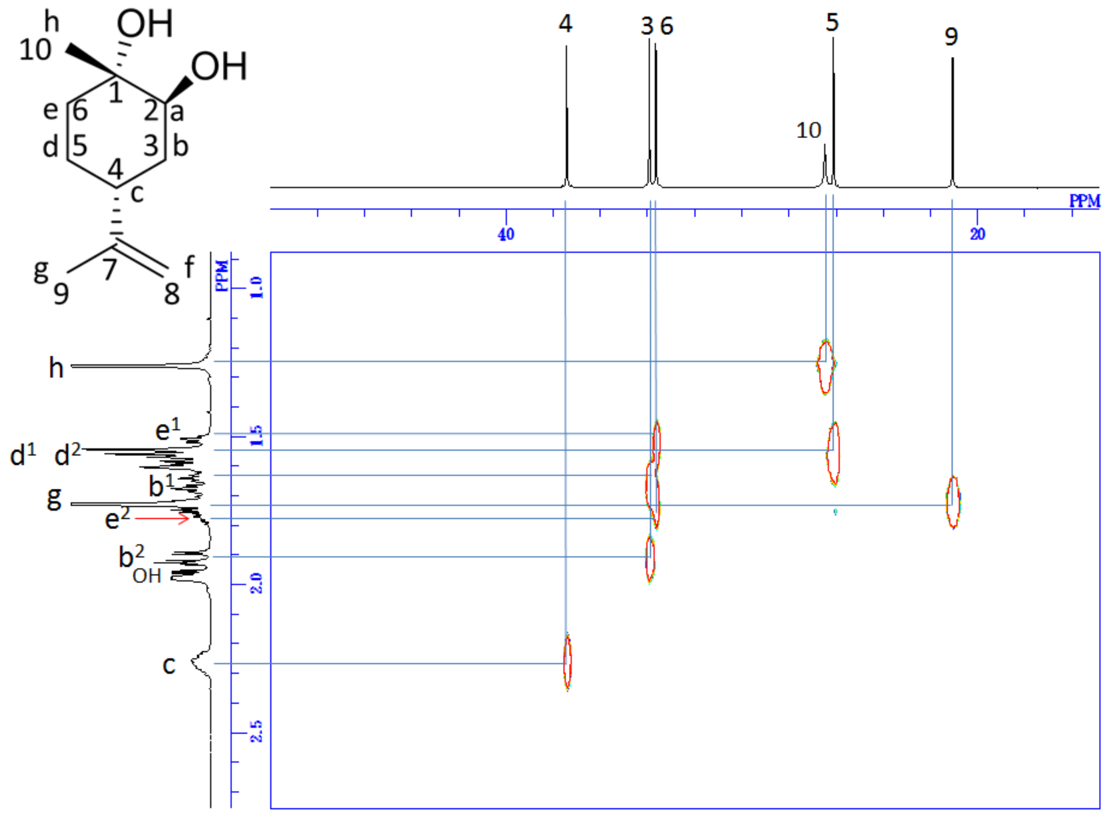

HETCOR spectra of **2b** in $CDCl_3$; (top) full range and (bottom) selected range.

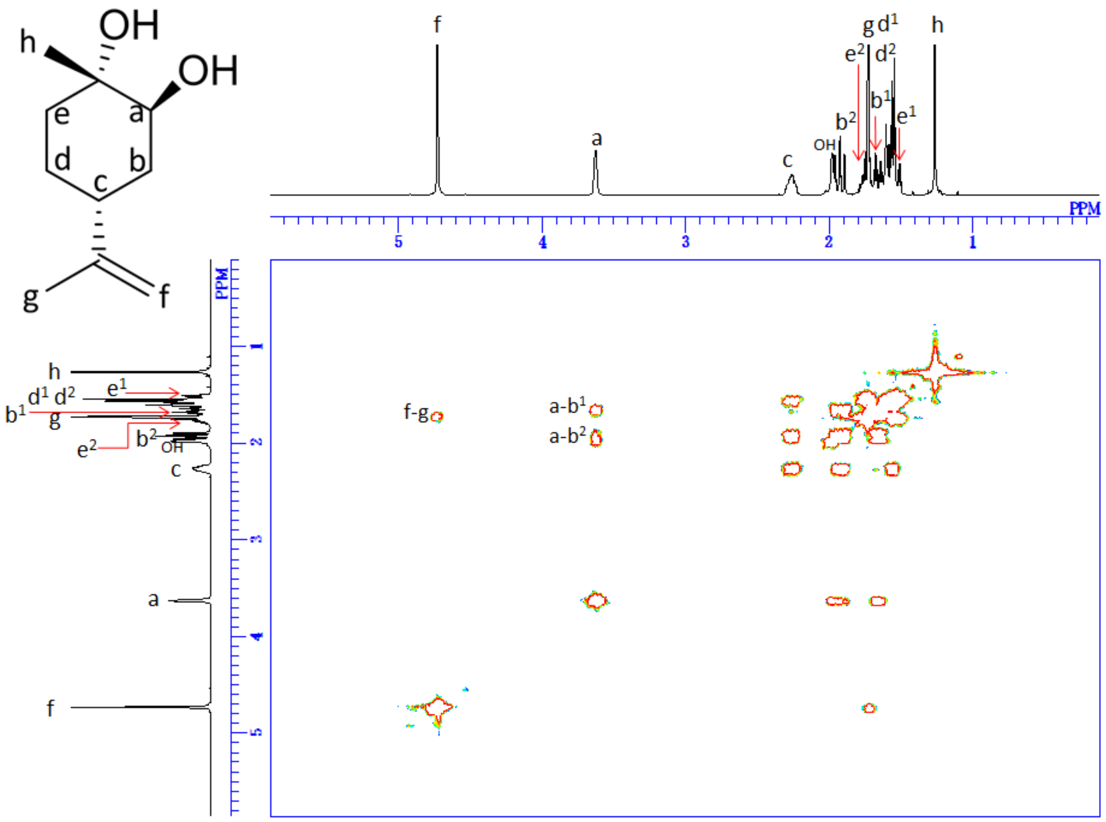

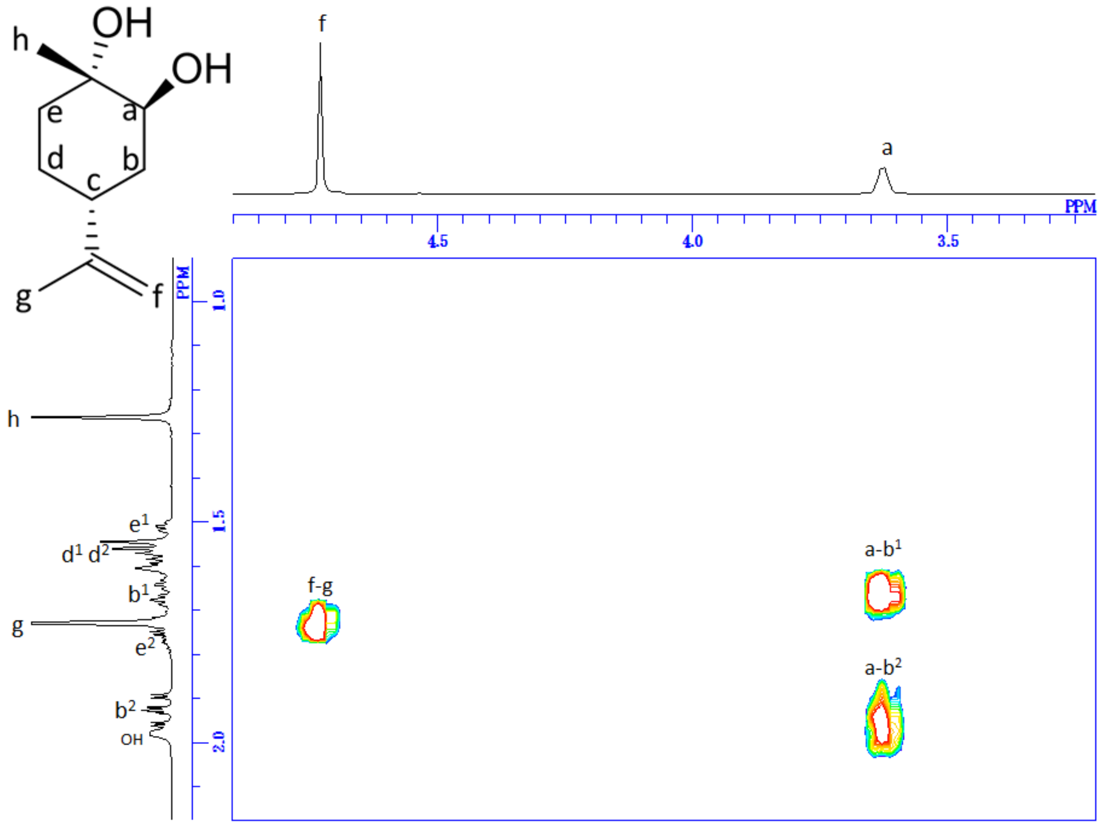

^{1}H-^{1}H COSY spectra of **2b** in $CDCl_3$; (top) full range and (bottom) selected range.

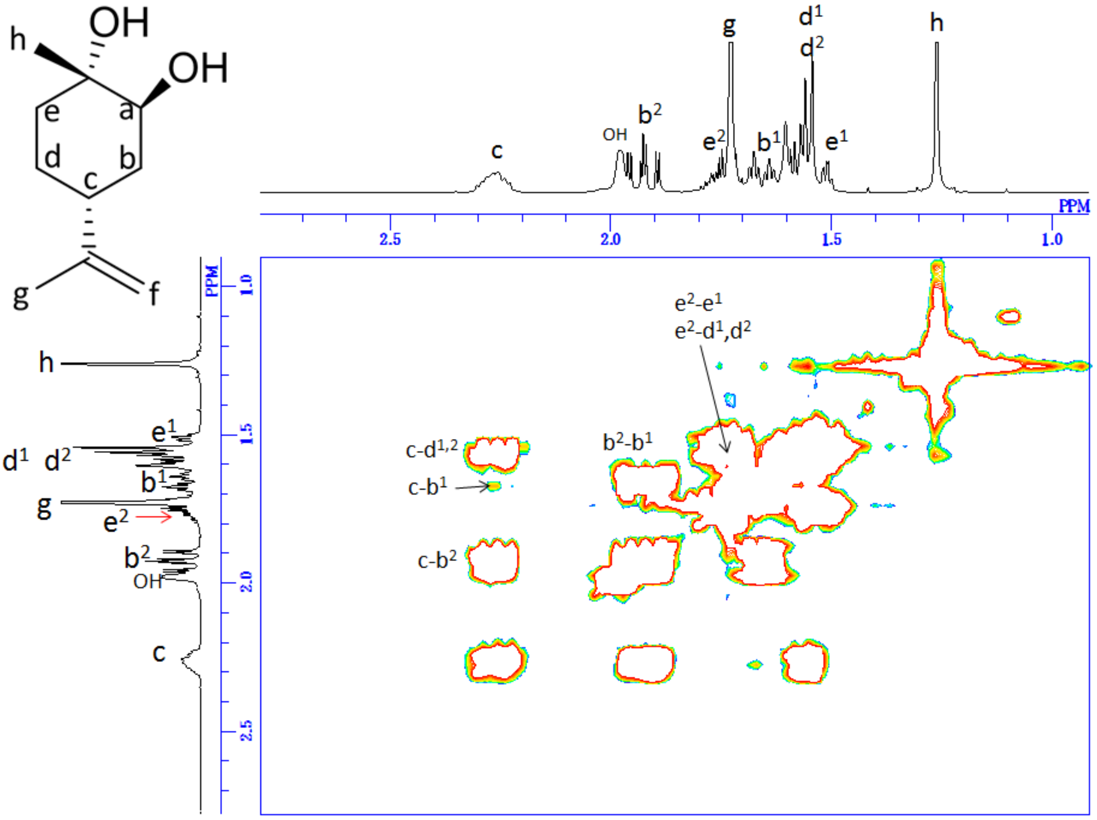

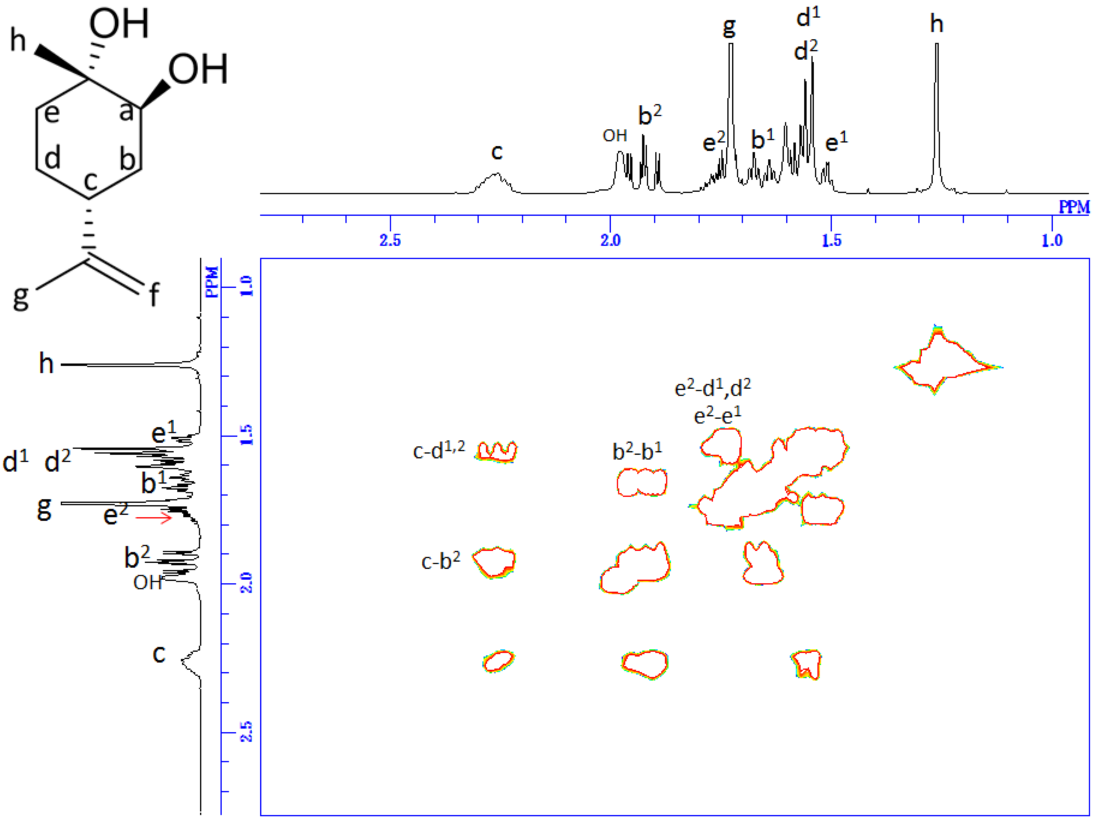

^{1}H-^{1}H COSY spectra of **2b** in $CDCl_3$; selected range with (top) high sensitivity and (bottom) low sensitivity.

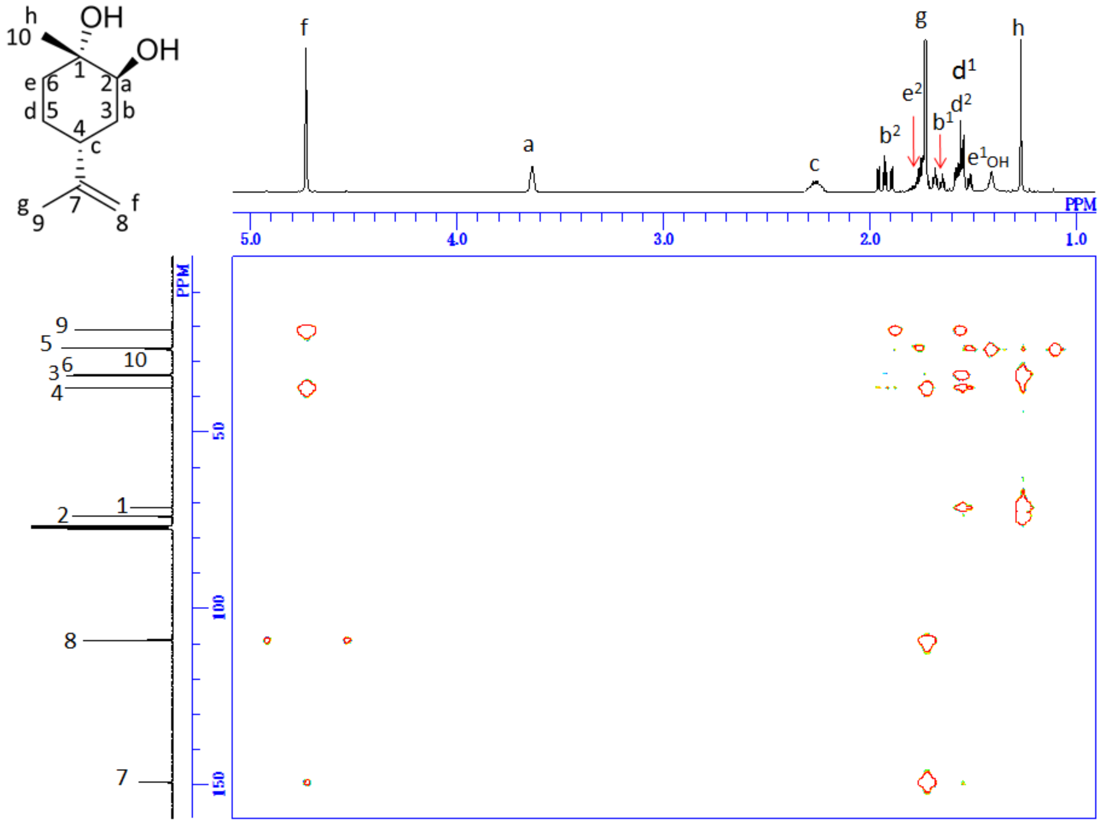

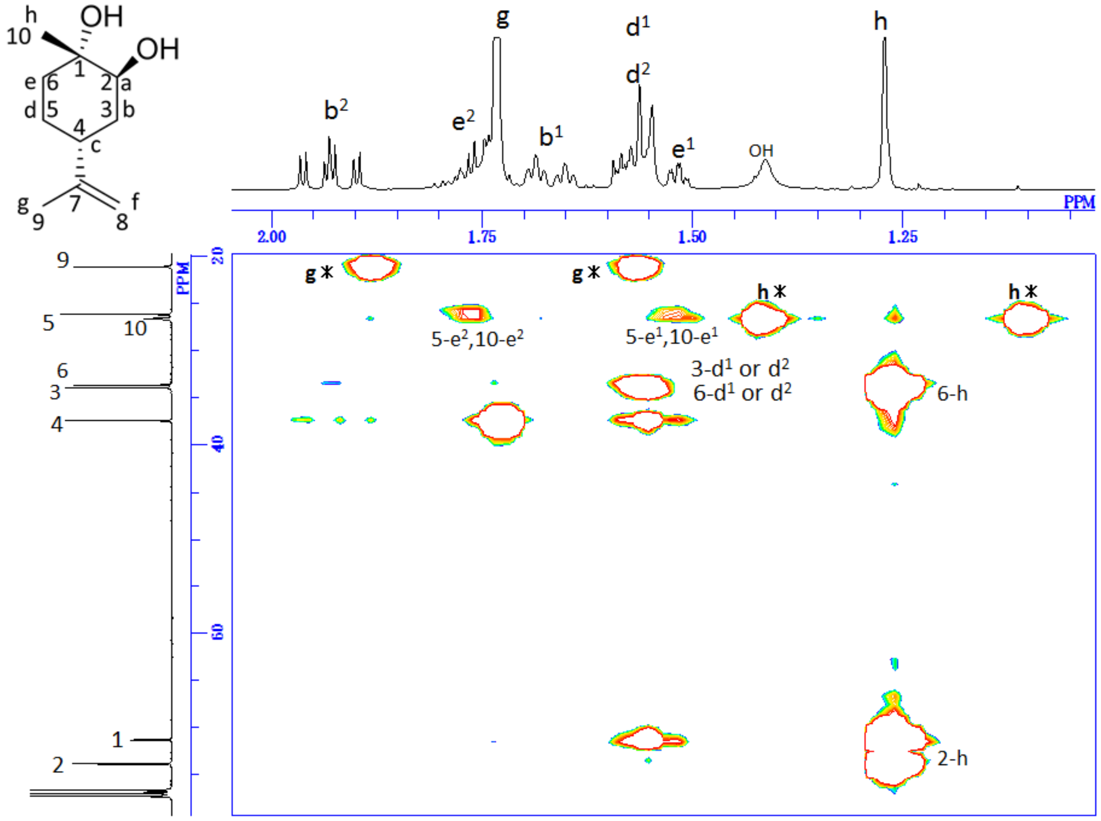

HMBC spectra of **2b** in $CDCl_3$; (top) full range and (bottom) selected range.
The marks (g* and h*) represent for side-band peaks.

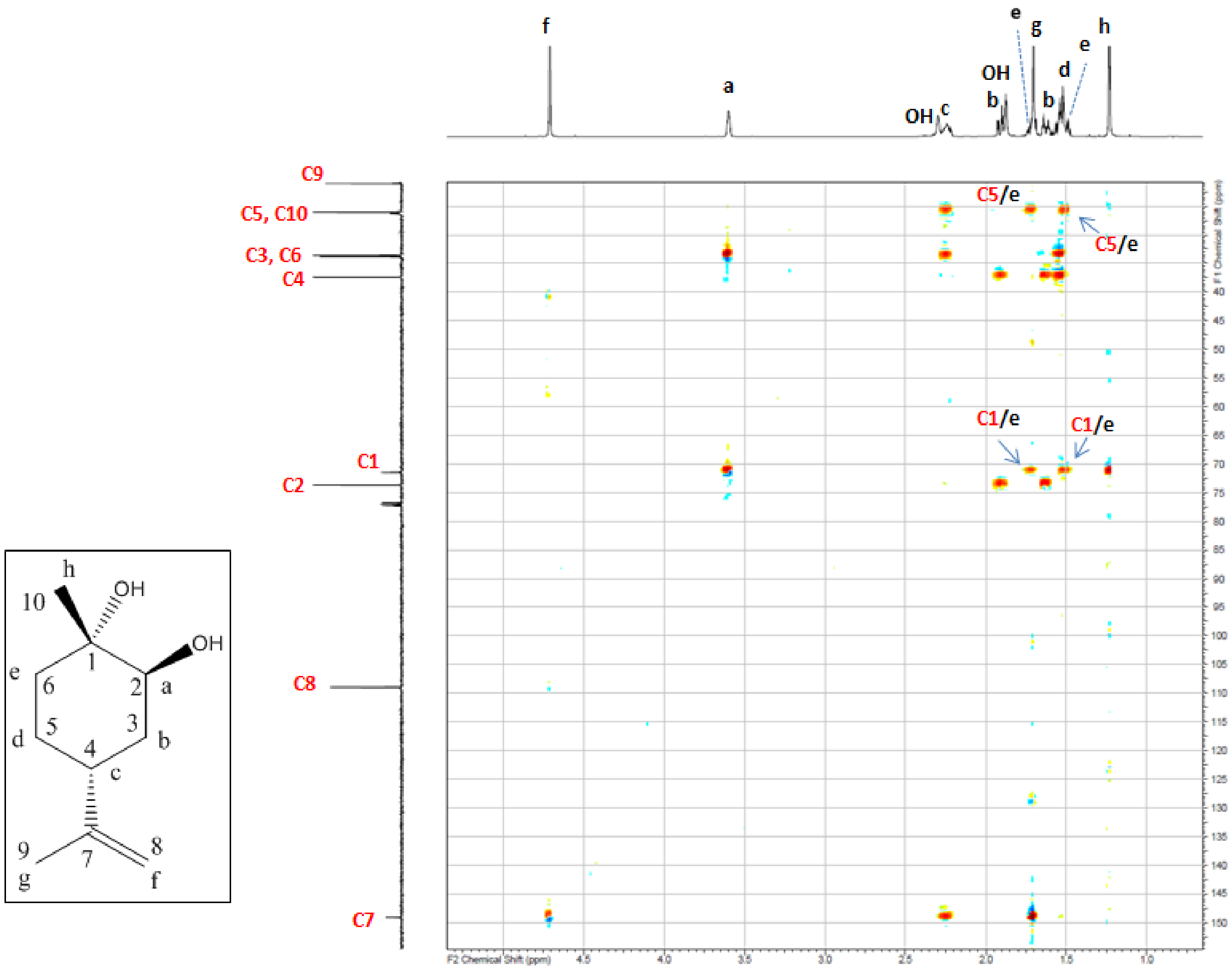

1,1-ADEQUATE spectrum of **2b** in $CDCl_3$.

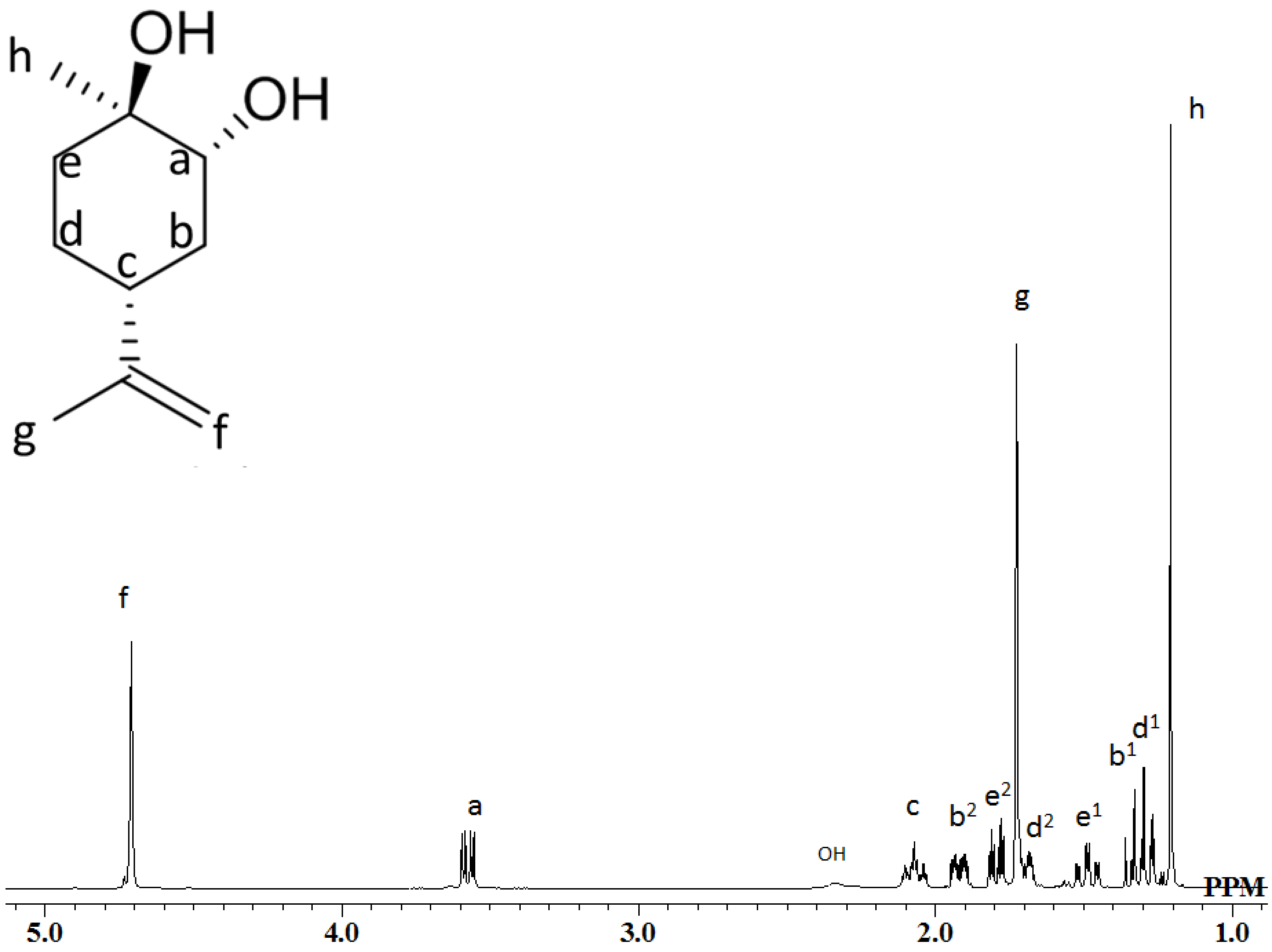

^{1}H NMR spectrum of **2c** in $CDCl_3$.

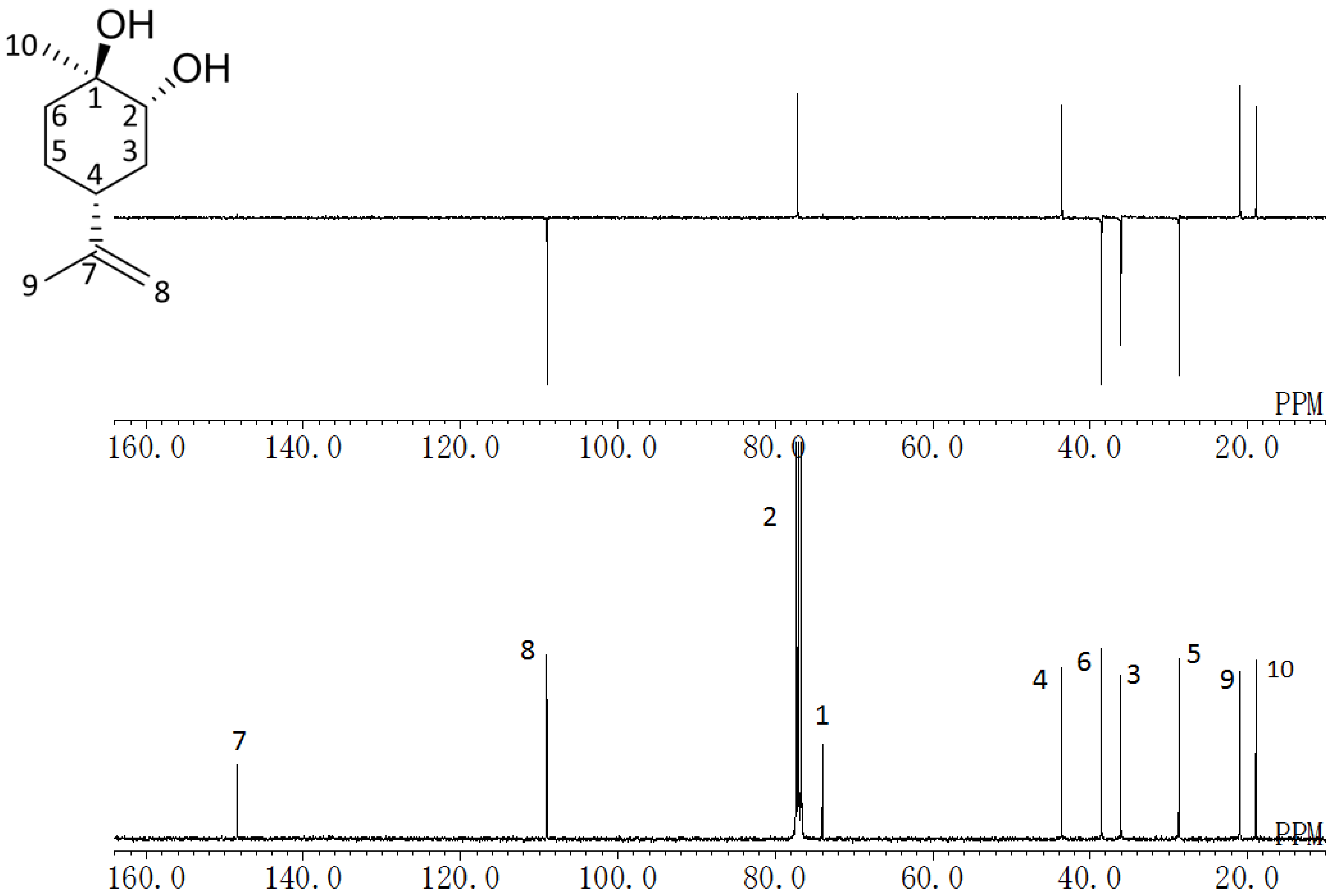

(top) DEPT135 and (bottom) ^{13}C NMR spectra of **2c** in $CDCl_3$.

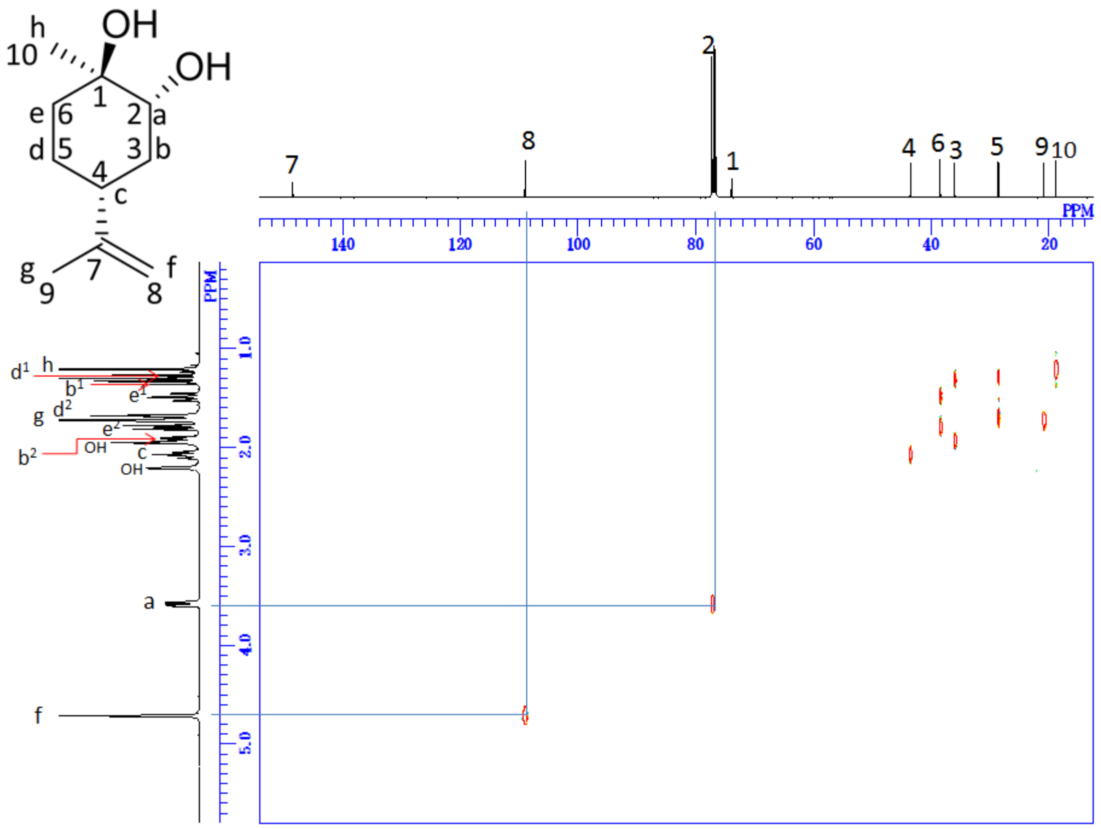

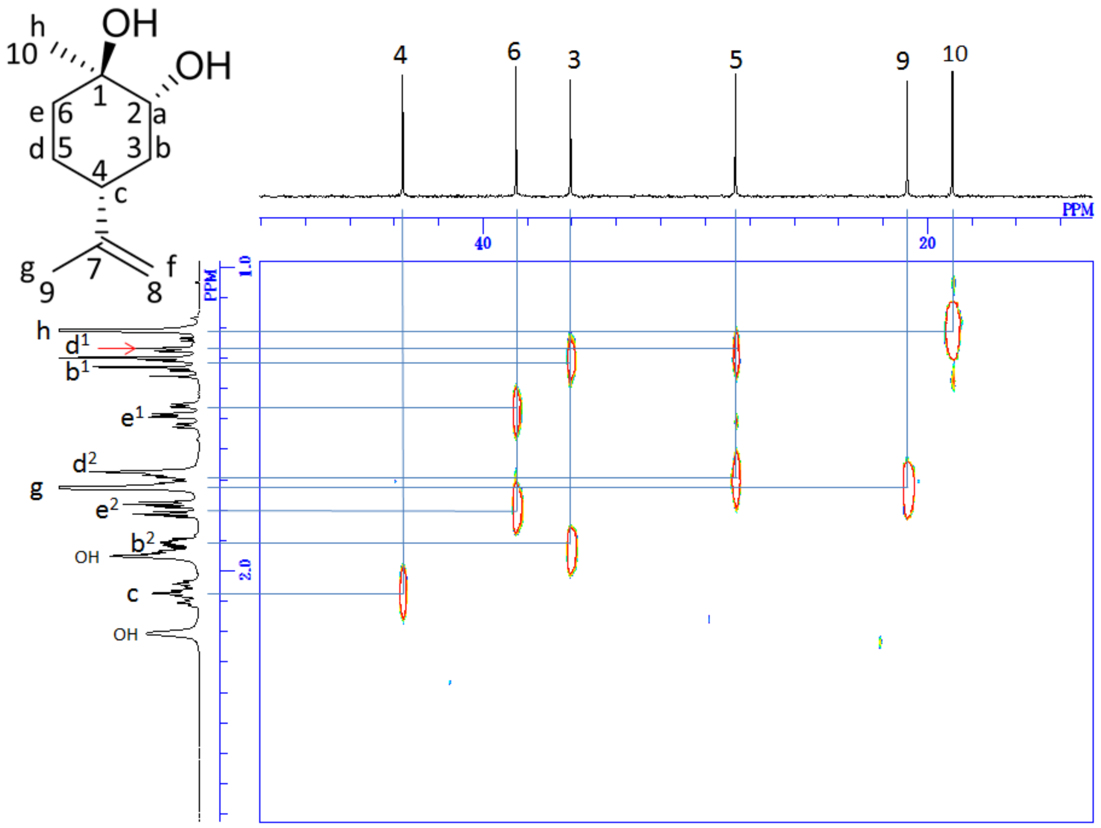

HETCOR spectra of **2c** in $CDCl_3$; (top) full range and (bottom) selected range.

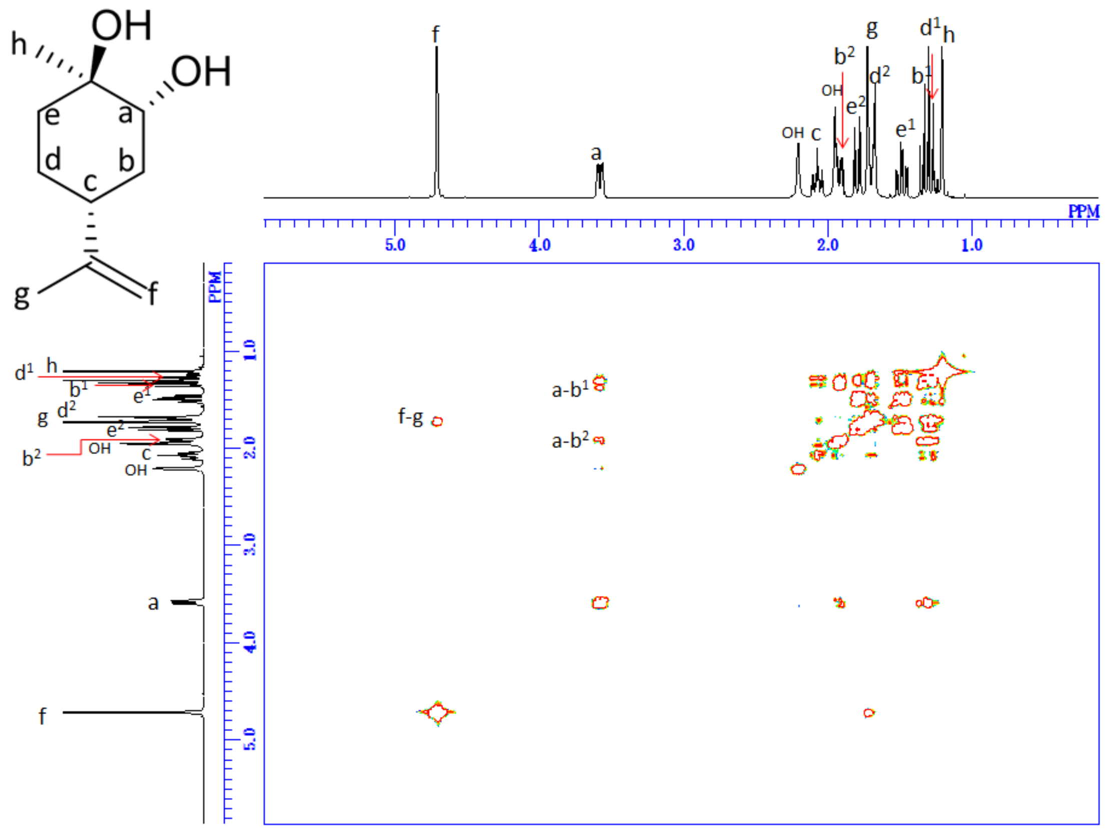

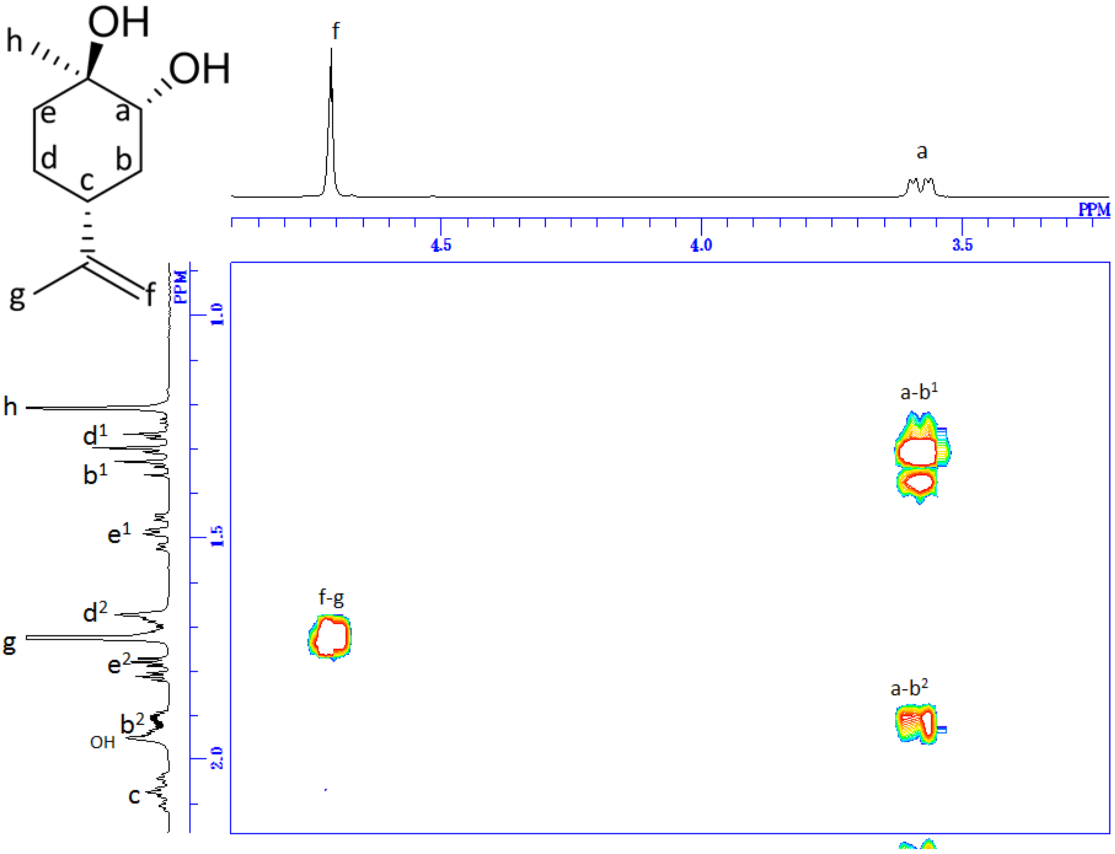

^{1}H-^{1}H COSY spectra of **2c** in $CDCl_3$; (top) full range and (bottom) selected range.

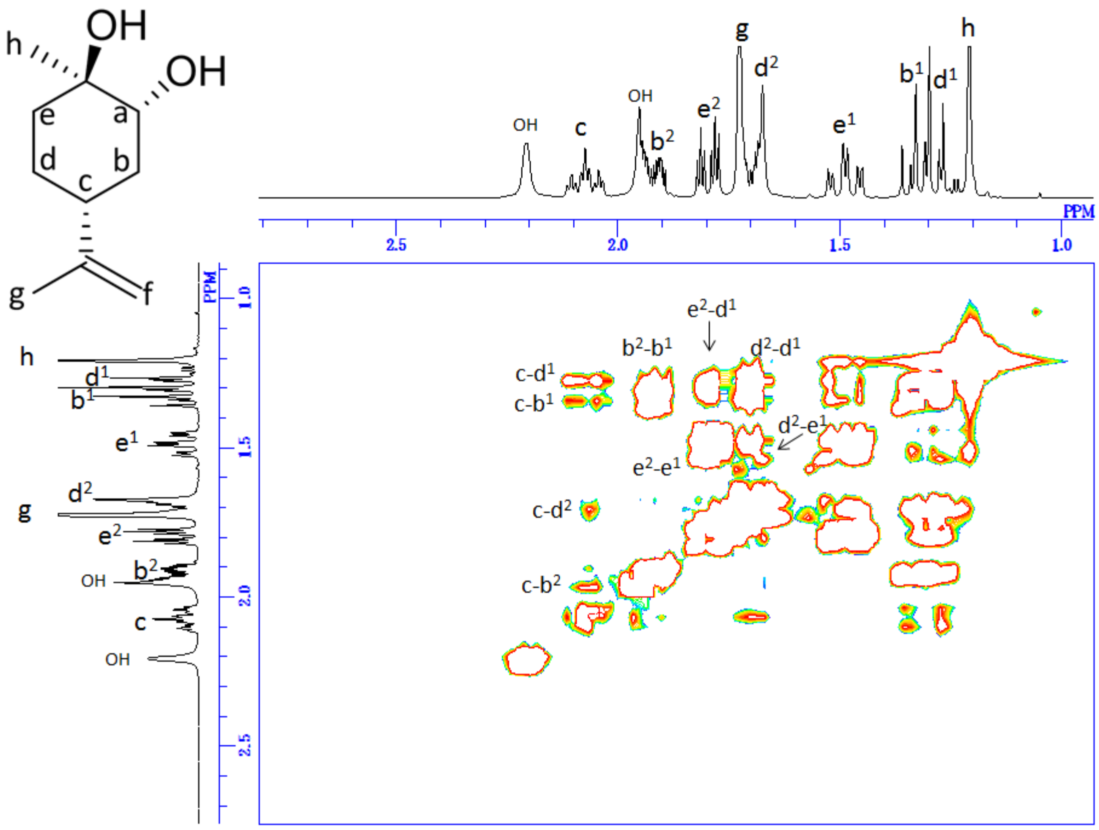

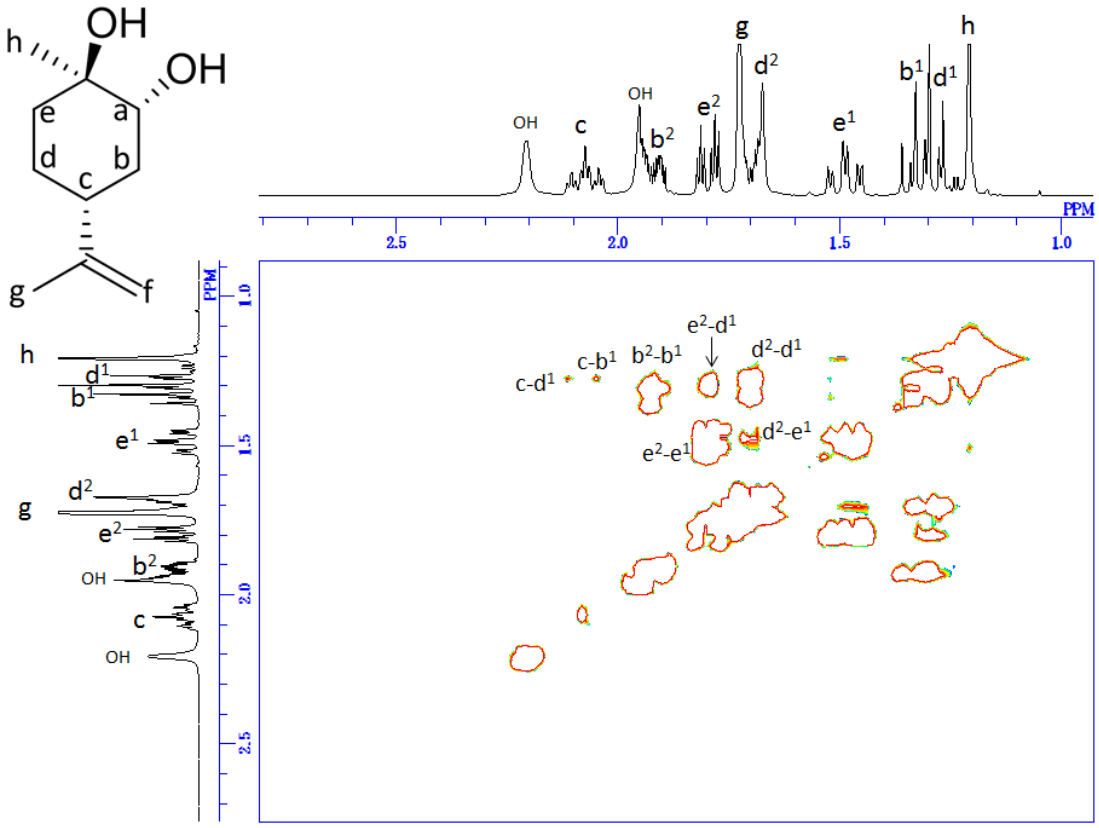

^{1}H-^{1}H COSY spectra of **2c** in $CDCl_3$; selected range with (top) high sensitivity and (bottom) low sensitivity.

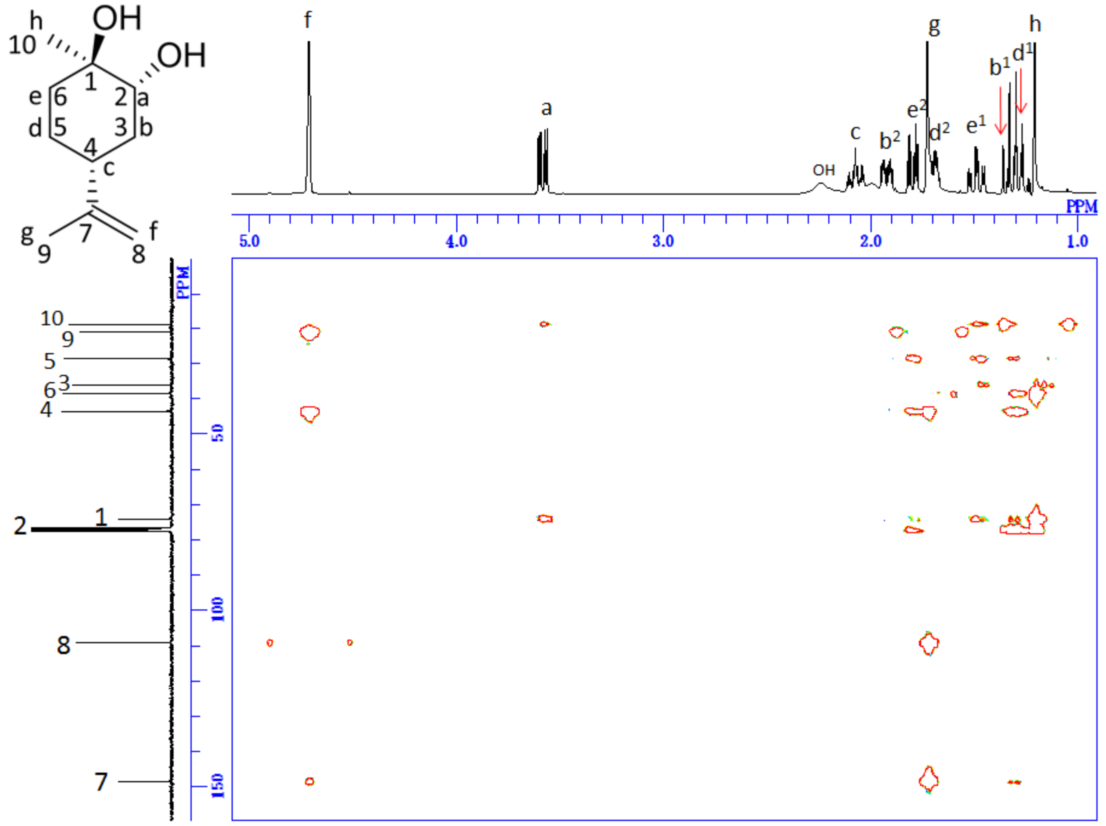

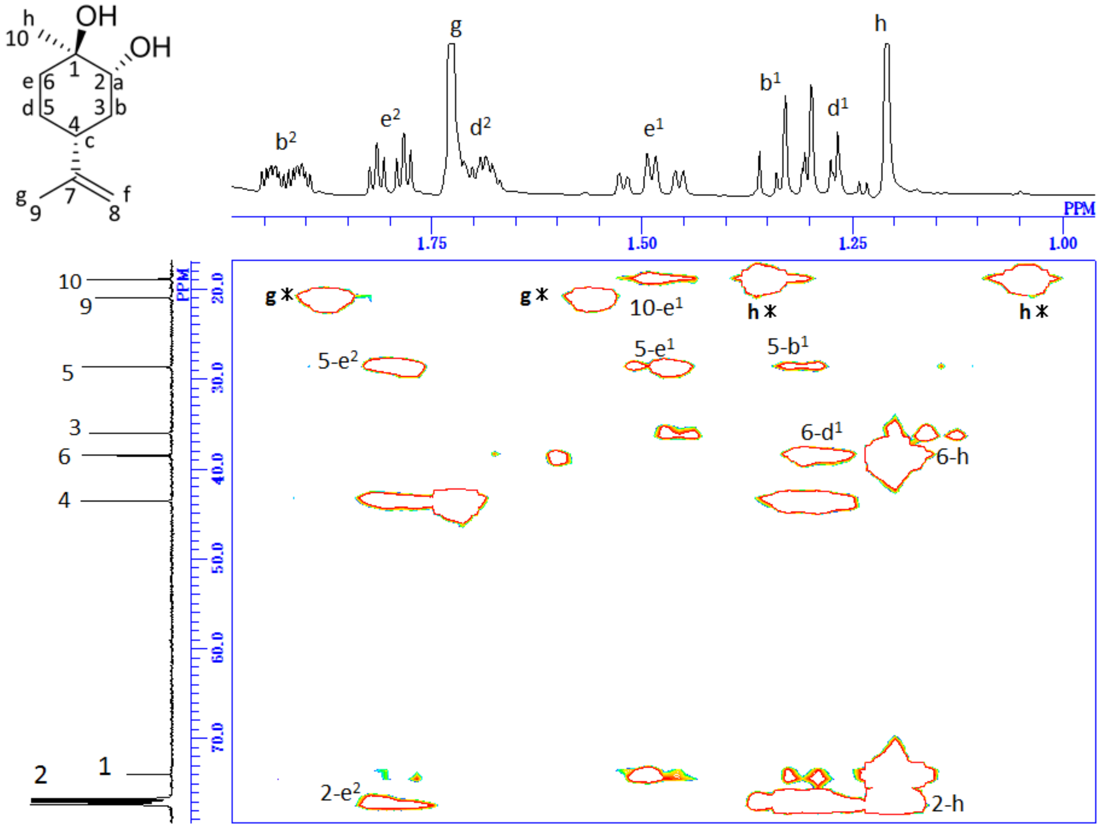

HMBC spectra of **2c** in $CDCl_3$; (top) full range and (bottom) selected range.
The marks (g* and h*) represent for side-band peaks.

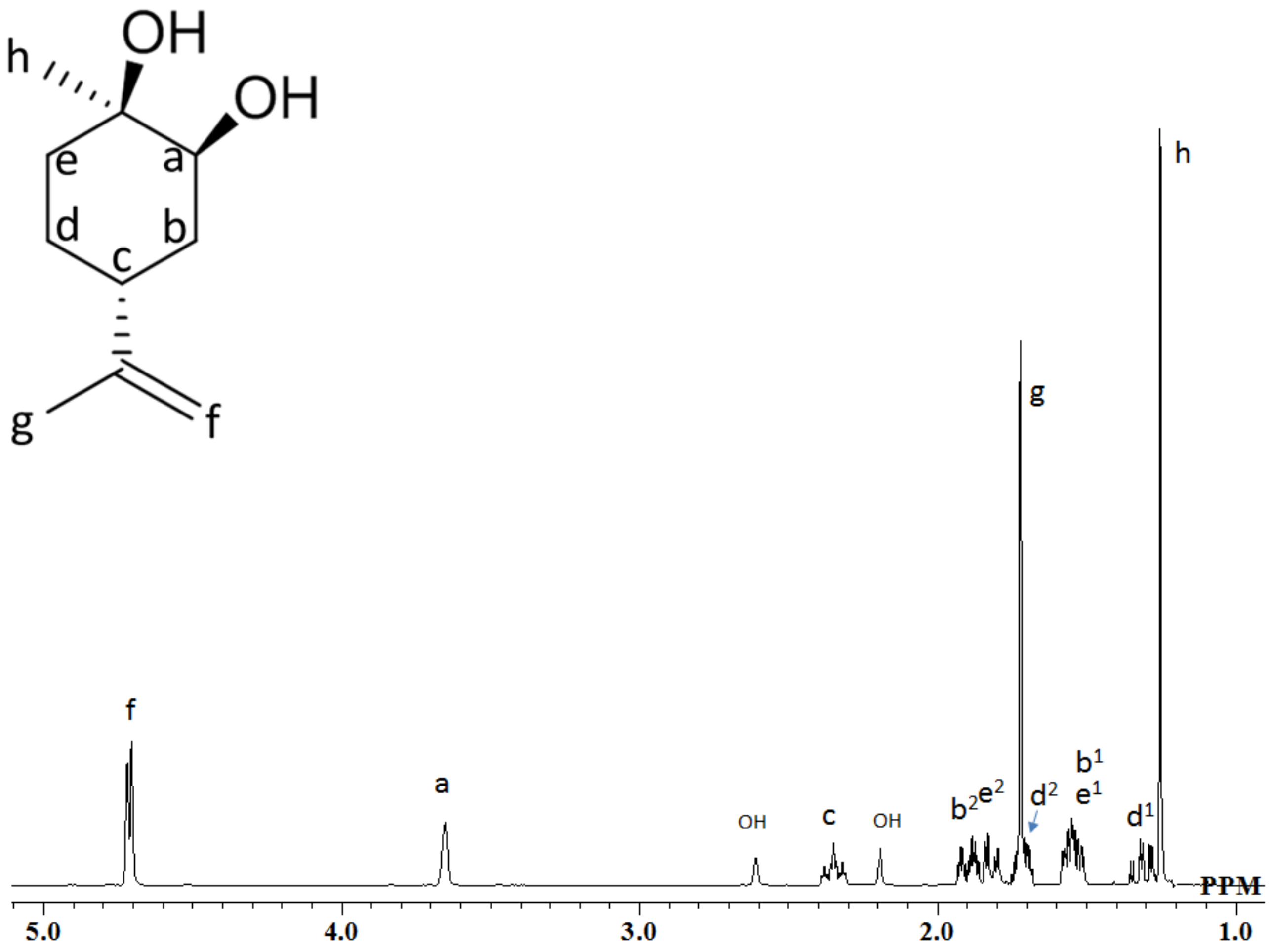

^{1}H NMR spectrum of **2d** in $CDCl_3$.

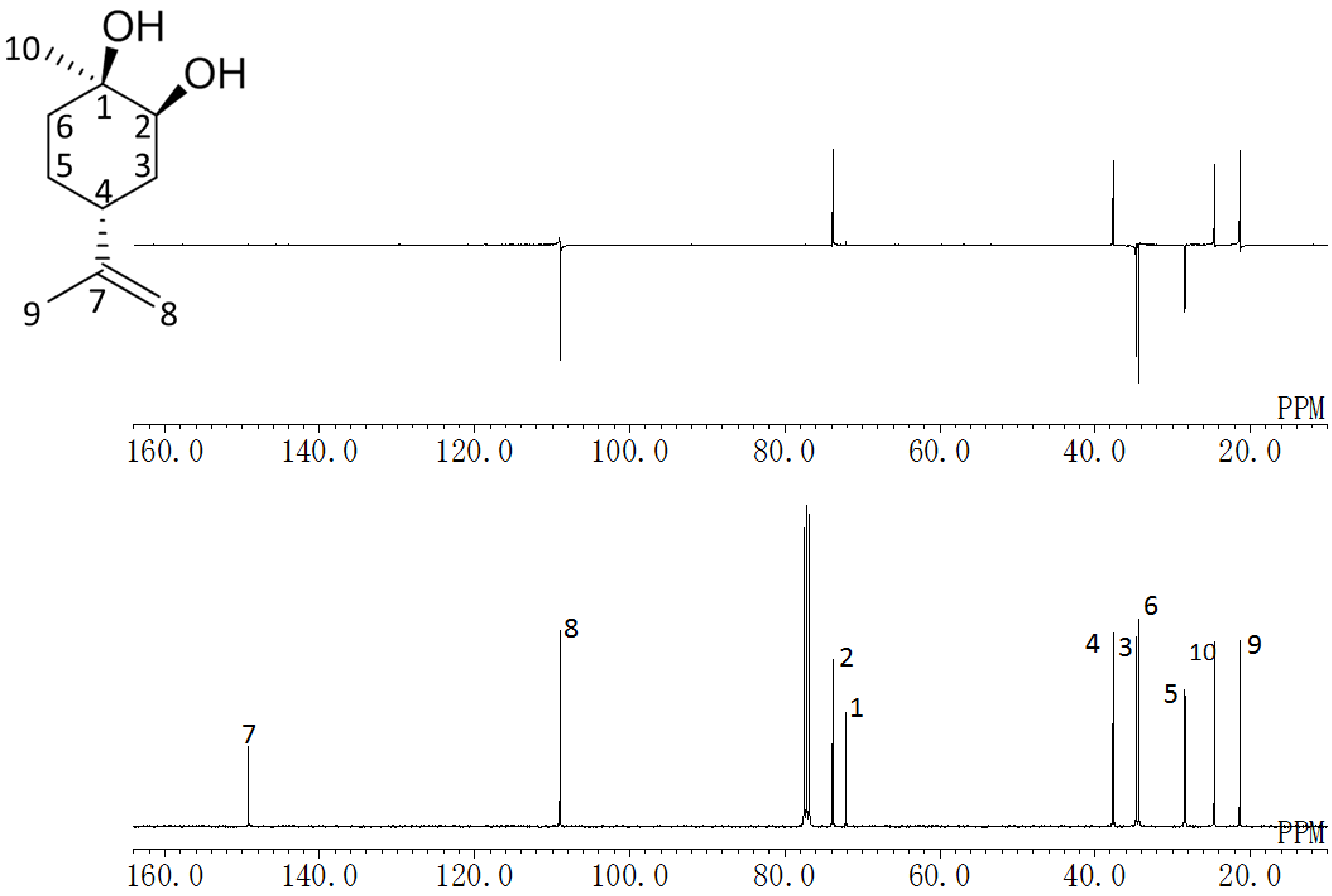

(top) DEPT135 and (bottom) ^{13}C NMR spectra of **2d** in $CDCl_3$.

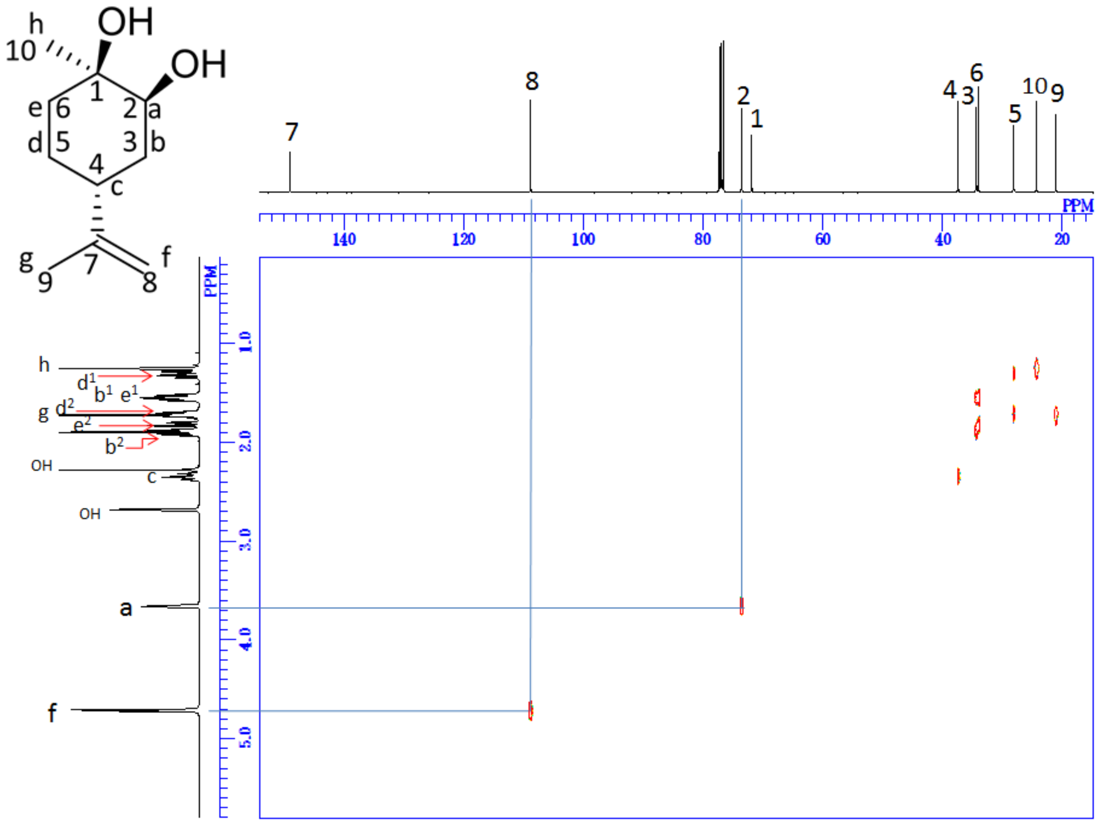

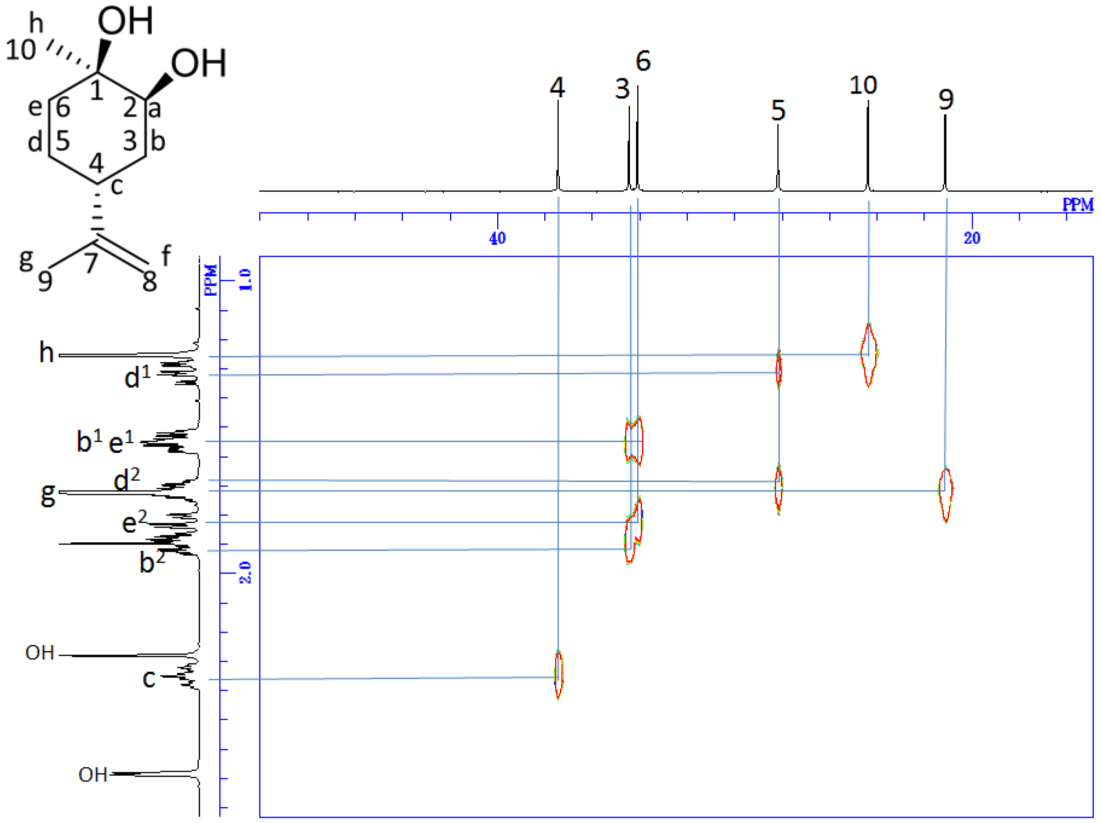

HETCOR spectra of **2d** in $CDCl_3$; (top) full range and (bottom) selected range.

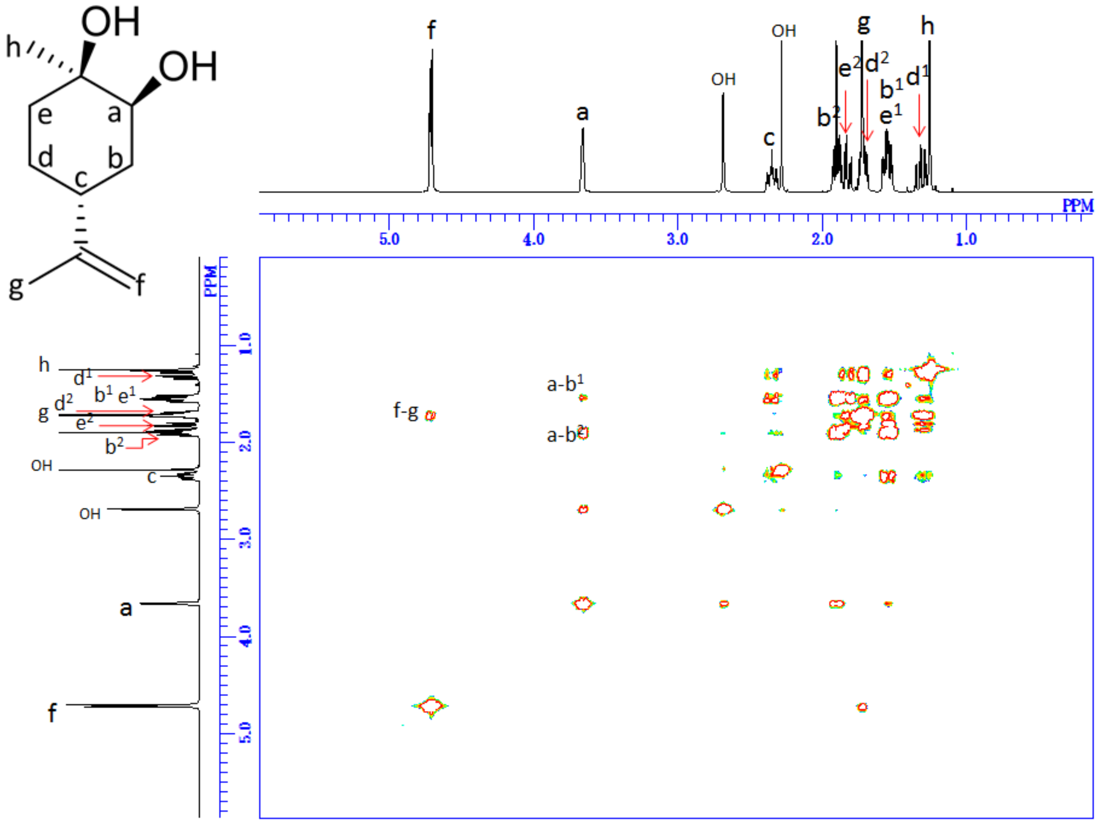

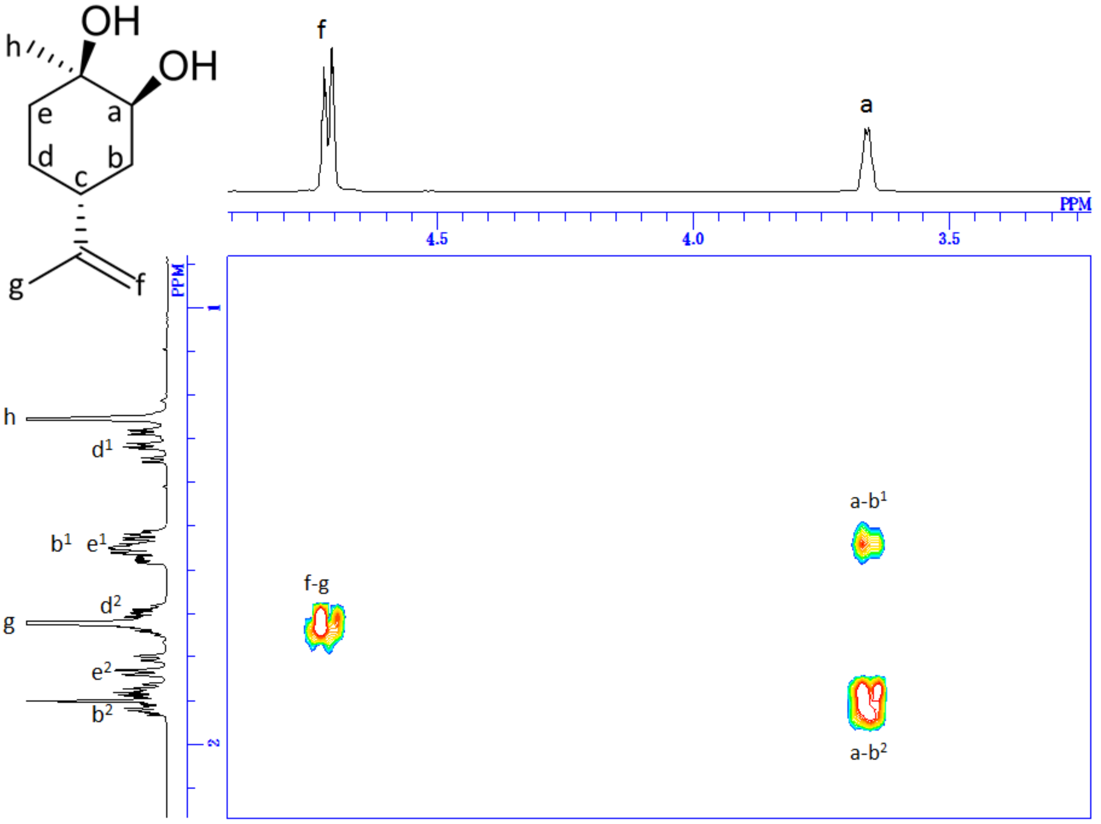

^{1}H-^{1}H COSY spectra of **2d** in $CDCl_3$; (top) full range and (bottom) selected range.

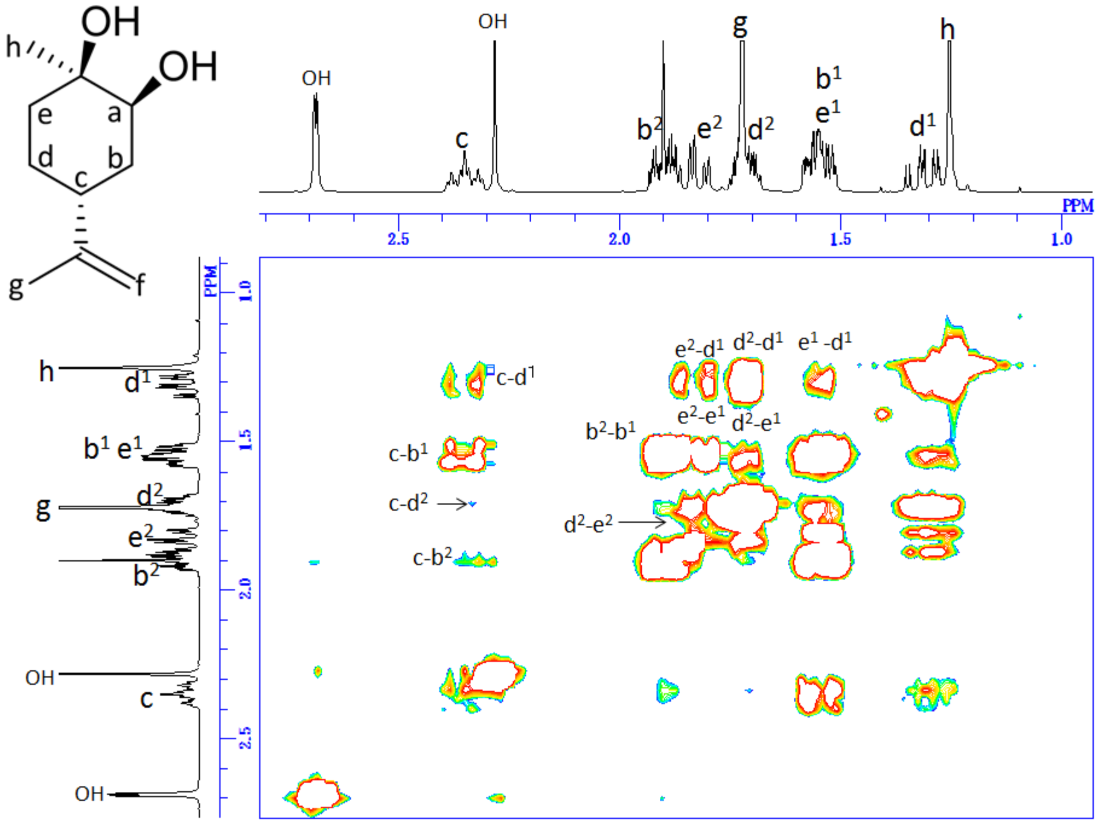

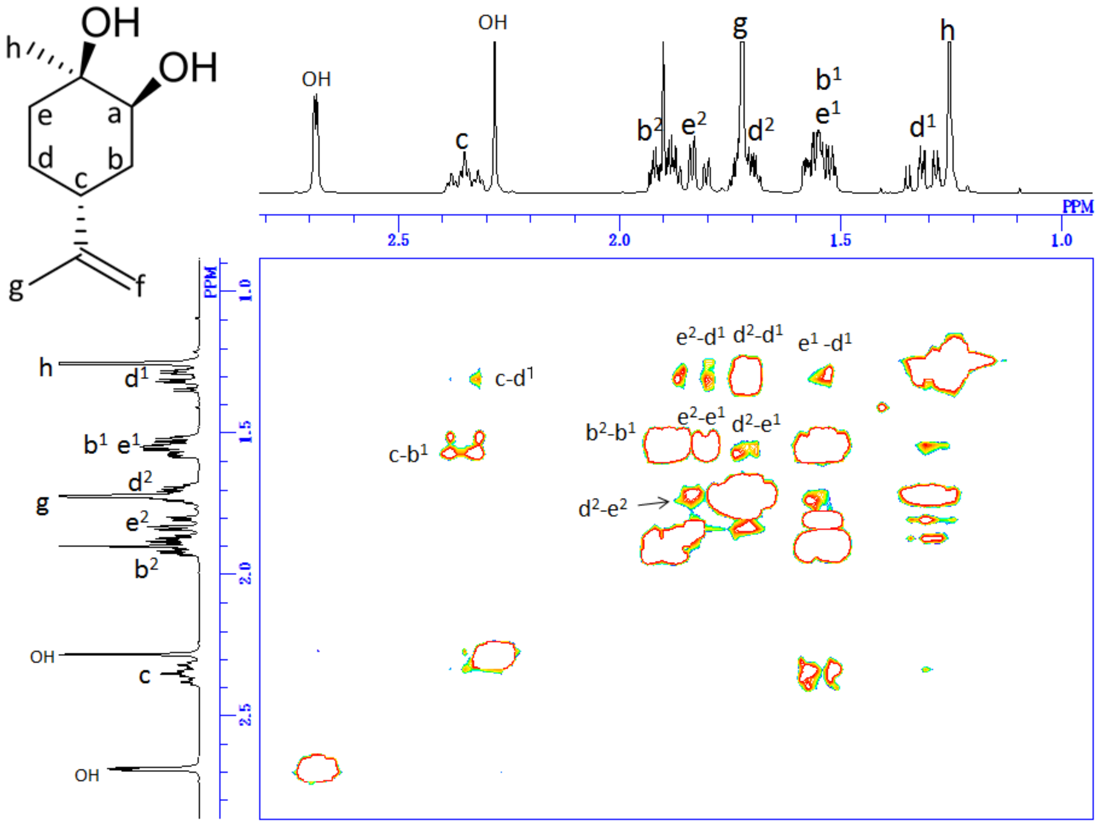

^{1}H-^{1}H COSY spectra of **2d** in $CDCl_3$; selected range with (top) high sensitivity and (bottom) low sensitivity.

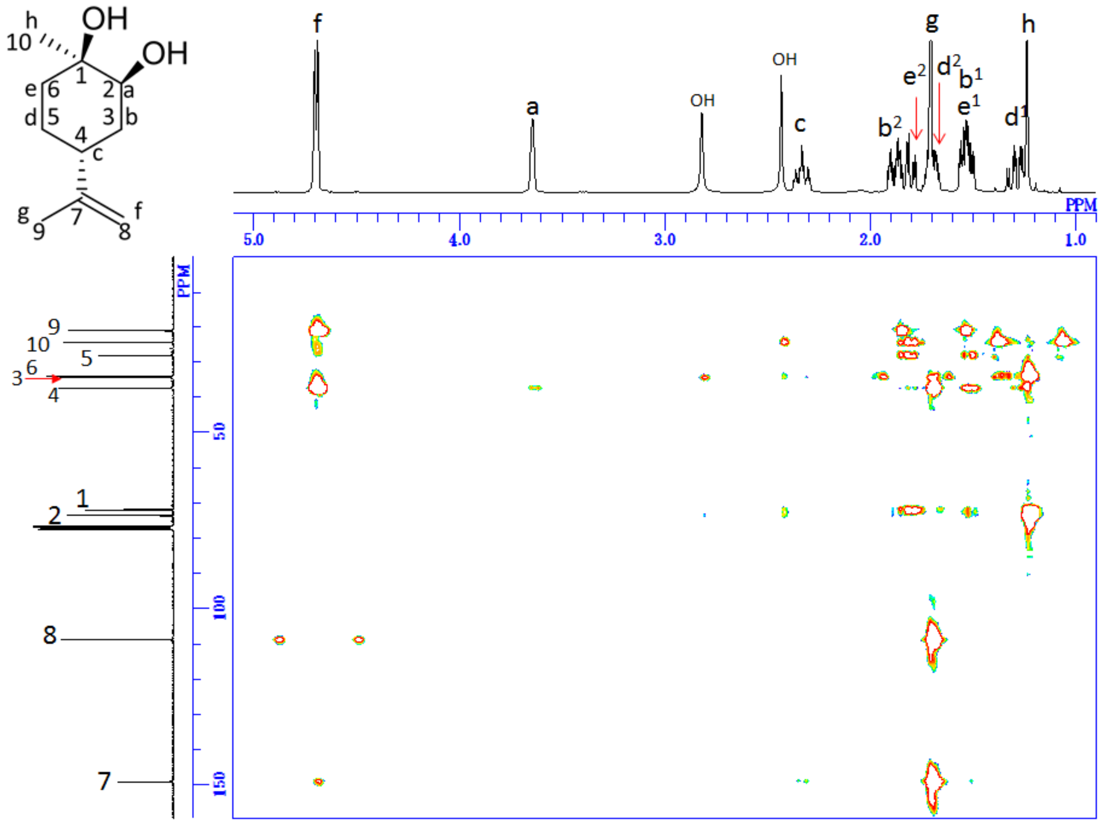

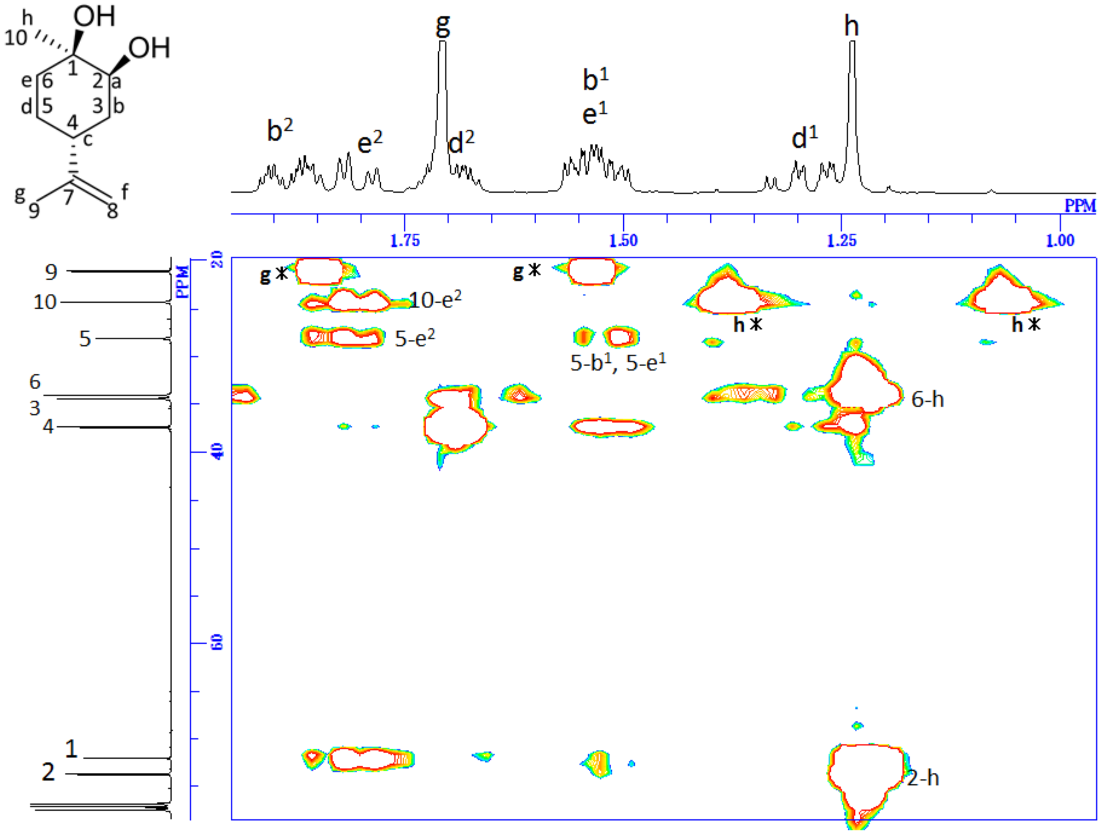

HMBC spectra of **2d** in $CDCl_3$; (top) full range and (bottom) selected range.
The marks (g* and h*) represent for side-band peak

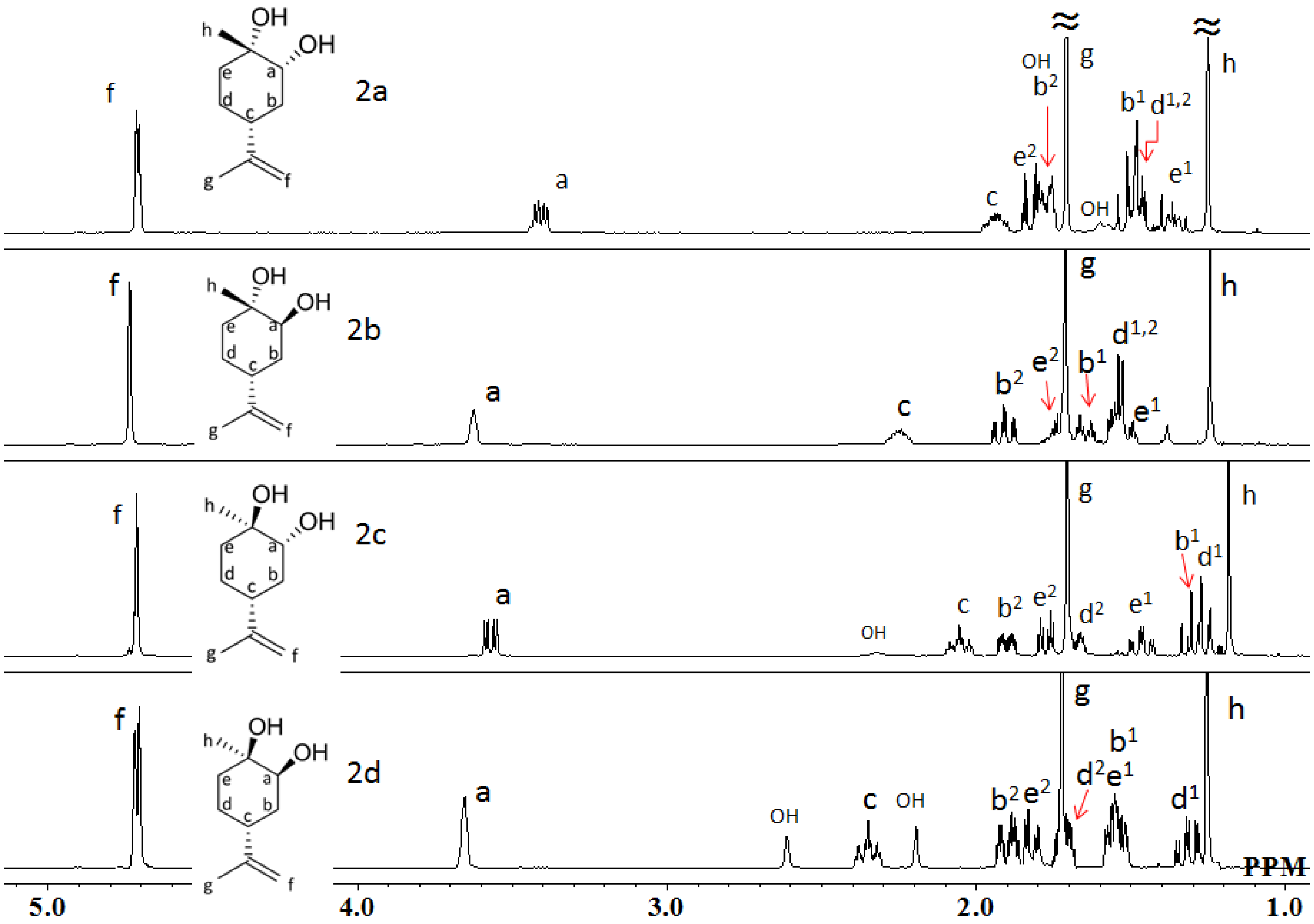

^{1}H NMR spectra of four LMdiols (**2a**–**2d**) in full scales in $CDCl_3$.

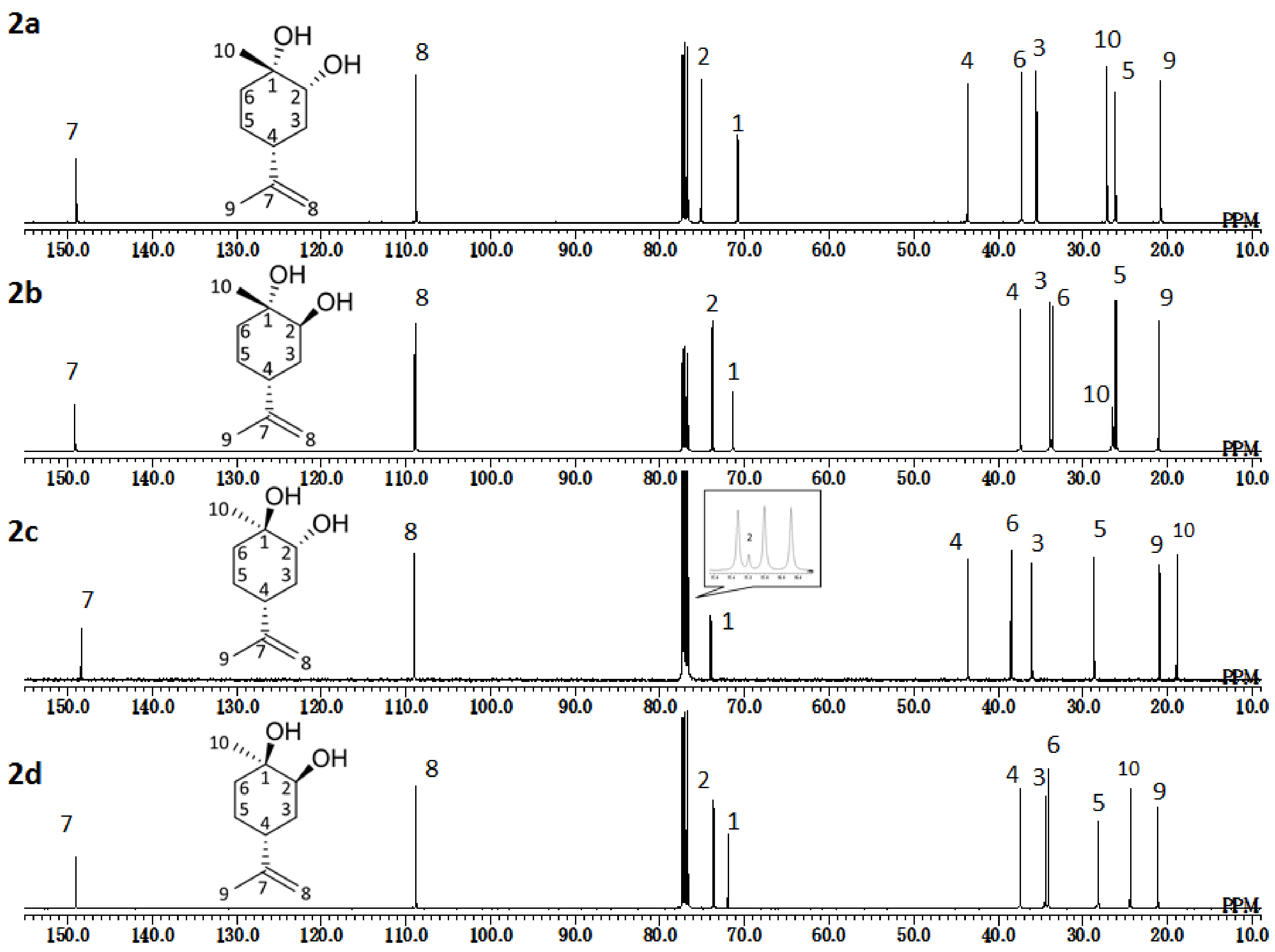

^{13}C NMR spectra of four LMdiols (**2a**–**2d**) in full scales in $CDCl_3$.

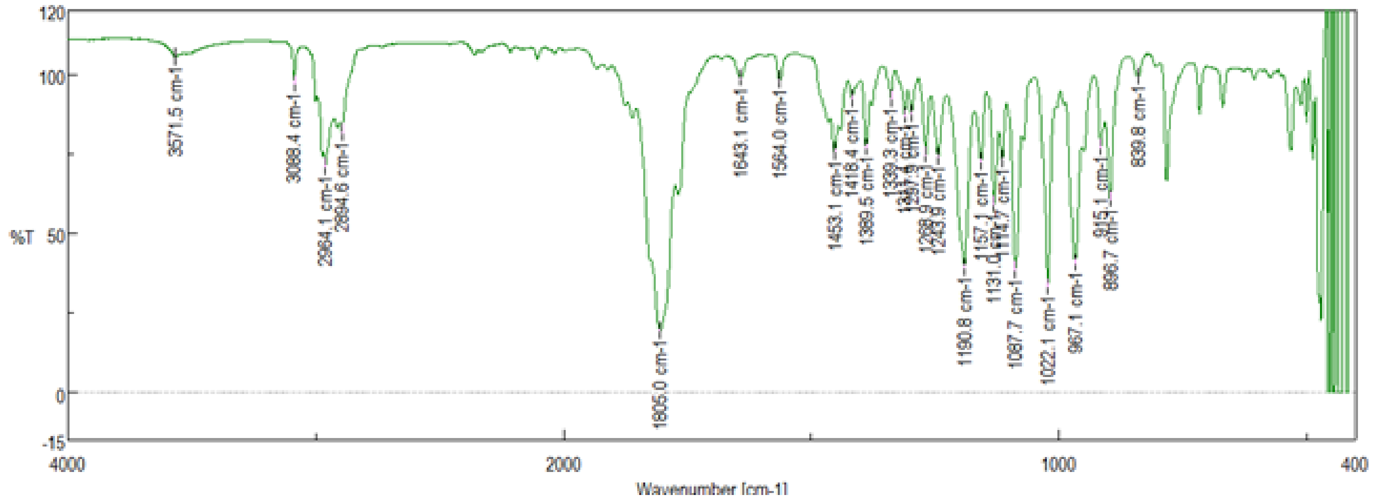

IR spectrum of **1b**

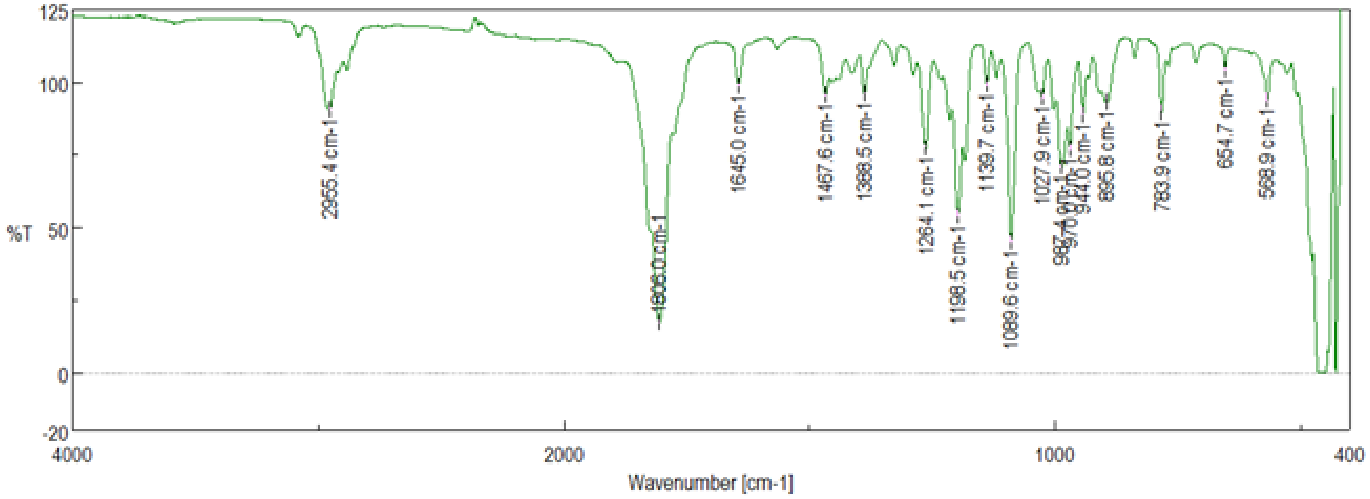

IR spectrum of **1c**.

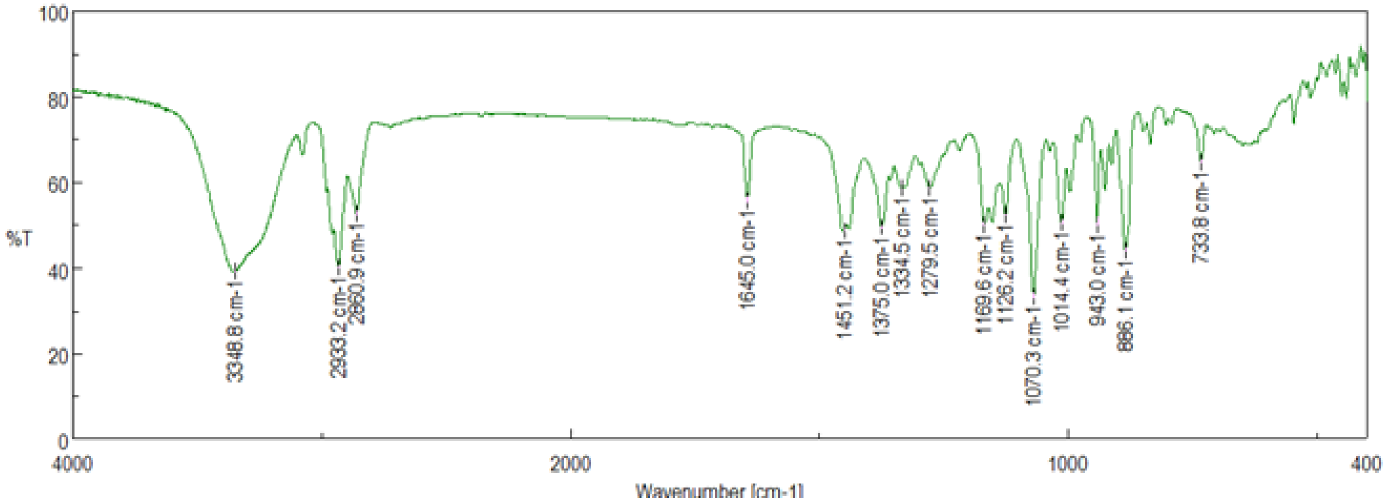

IR spectrum of **2a**.

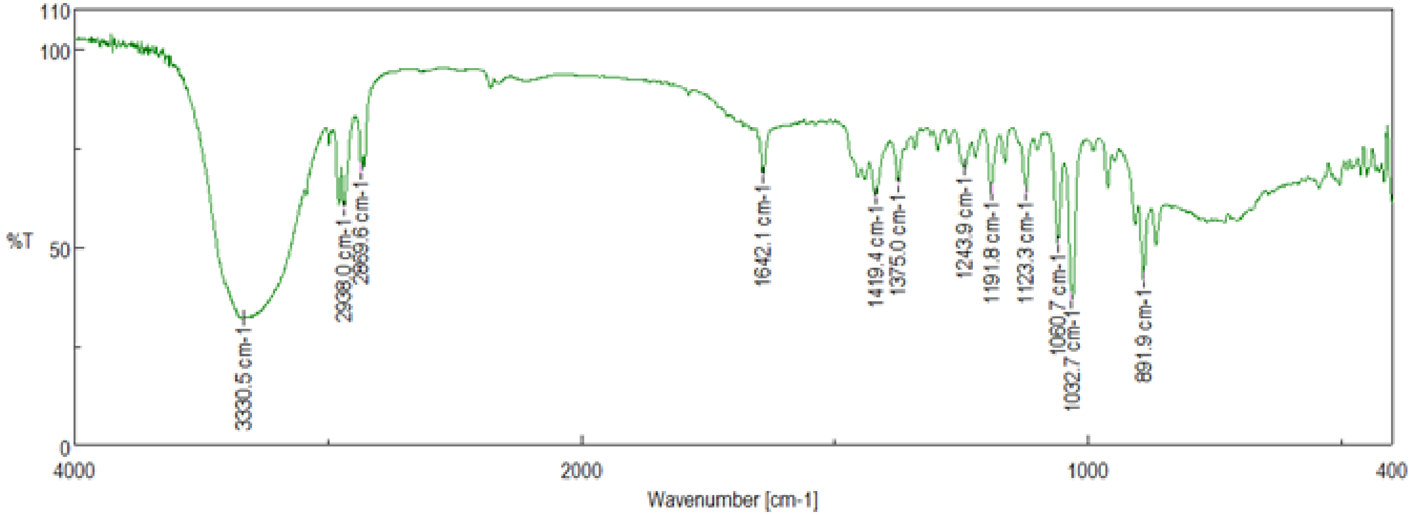

IR spectrum of **2b**.

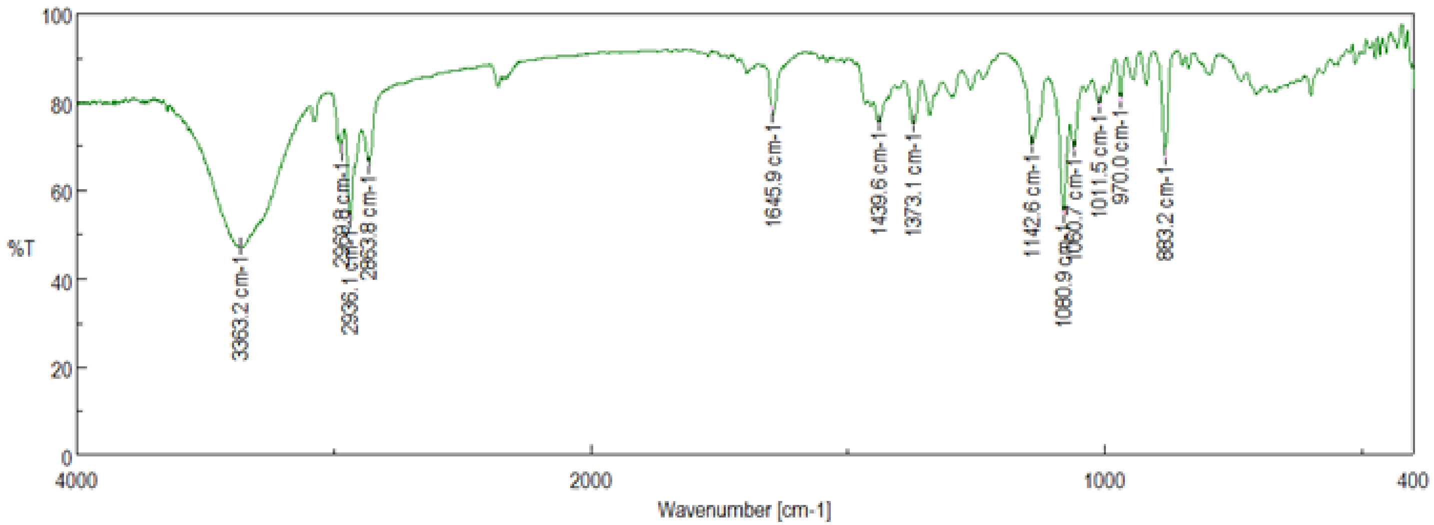

IR spectrum of **2c**.

IR spectra for **1a**, **1d**, and **2d** were reported in Supporting Information of ref.[2].

C-R8A CHROMATOPAC CH=1 DATA=1:@CHRM1.C00 ATTEN= 7 SPEED= 5.0

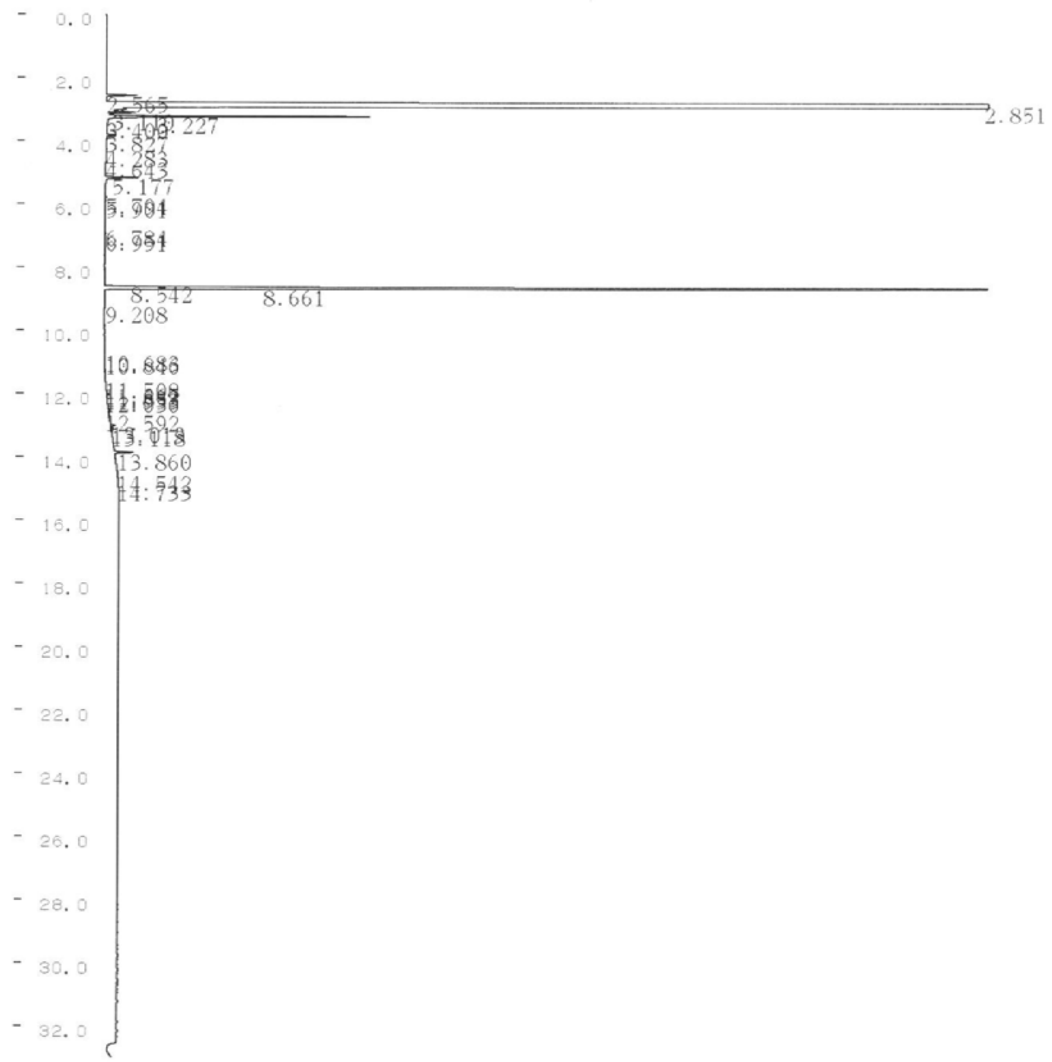

C-R8A CHROMATOPAC CH=1 Report No.=9 DATA=1:@CHRM1.C00 18/11/12 18:42:24

** CALCULATION REPORT **

CH	PKNO	TIME	AREA	HEIGHT	MK	IDNO	CONC	NAME
1	1	2.565	9257	4499			0.0026	
	2	2.851	356674816	101724392	E		99.8037	
	3	3.11	15805	4193	V		0.0044	
	4	3.227	85899	38222	SV		0.024	
	6	3.827	3244	1394	T		0.0009	
	7	4.283	374	150			0.0001	
	8	4.643	109	36			0	
	9	5.177	11519	4638			0.0032	
	10	5.794	538	252			0.0002	
	11	5.901	434	181			0.0001	
	12	6.784	306	115			0.0001	
	13	6.951	598	236	V		0.0002	
	14	8.542	491	86			0.0001	
	15	8.661	551748	197526	V		0.1544	
	16	9.208	471	84			0.0001	
	18	10.846	121	44			0	
	19	11.508	189	28			0.0001	
	20	11.667	176	29			0	
	22	11.953	291	92			0.0001	
	23	12.036	598	239	V		0.0002	
	24	12.592	703	46	V		0.0002	
	25	13.013	2756	782			0.0008	
	26	13.118	1497	481	V		0.0004	
	27	13.86	10784	2914	V		0.003	
	28	14.542	3583	60	V		0.001	
	29	14.733	659	46	V		0.0002	

GC chart of compound **1a** as a typical sample.

C-R8A CHROMATOPAC CH=1 DATA=1:@CHRM1.C00 ATTEN= 7 SPEED= 5.0

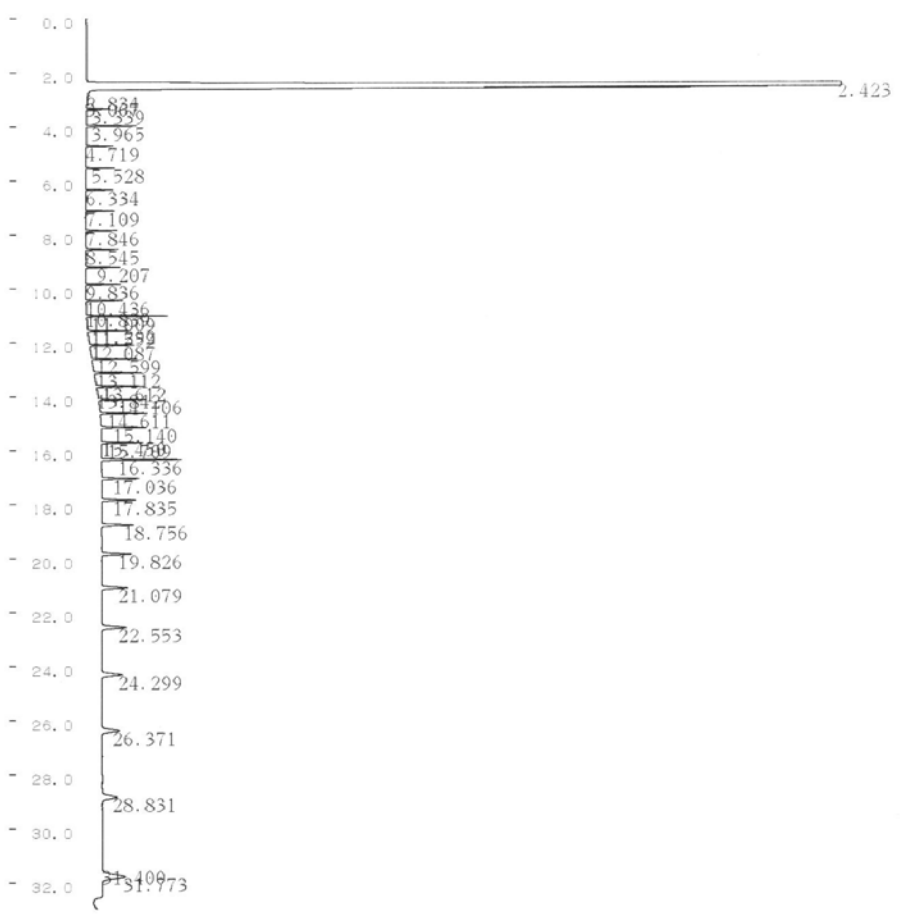

C-R8A CHROMATOPAC CH=1 Report No.=9 DATA=1:@CHRM1.C00 18/11/09 15:54:32

** CALCULATION REPORT **

CH	PKNO	TIME	AREA	HEIGHT	MK	IDNO	CONC	NAME
1	1	2.423	128961008	46932592	S		99.5101	
	2	2.834	495	227	T		0.0004	
	4	3.339	8759	4020			0.0068	
	5	3.965	17913	8508			0.0138	
	6	4.719	9117	4585			0.007	
	7	5.528	9444	4906			0.0073	
	8	6.334	9436	4762			0.0073	
	9	7.109	9996	5013			0.0077	
	10	7.846	10521	5320			0.0081	
	11	8.545	11938	5529			0.0092	
	12	9.207	12248	5757			0.0095	
	13	9.836	12510	5988			0.0097	
	14	10.436	13197	6239			0.0102	
	15	10.839	325	90			0.0003	
	16	11.009	30139	13705	V		0.0233	
	18	11.559	15622	7087			0.0121	
	19	12.087	16855	7620			0.013	
	20	12.599	18896	7709			0.0146	
	21	13.112	22005	8200	V		0.017	
	22	13.612	24882	8259	V		0.0192	
	23	13.842	2468	227	V		0.0019	
	24	14.106	23788	8250	V		0.0184	
	25	14.611	26094	8144	V		0.0201	
	26	15.14	22563	7468	V		0.0174	
	27	15.45	157	20			0.0001	
	28	15.709	20109	7049	V		0.0155	
	29	16.336	40644	13597			0.0314	
	30	17.036	20723	6327			0.016	
	31	17.835	20962	5822			0.0162	
	32	18.756	21423	5458			0.0165	
	33	19.826	22156	4959			0.0171	
	34	21.079	22163	4421			0.0171	
	35	22.553	23596	4103			0.0182	
	36	24.299	22890	3411			0.0177	
	37	26.371	23022	2977			0.0178	
	38	28.831	22772	2452			0.0176	
	39	31.4	452	46			0.0003	
	40	31.773	44746	3970	V		0.0345	
		TOTAL	129595776	47134752			100	

GC chart of mixed hydrocabon standards (C9 - C40).

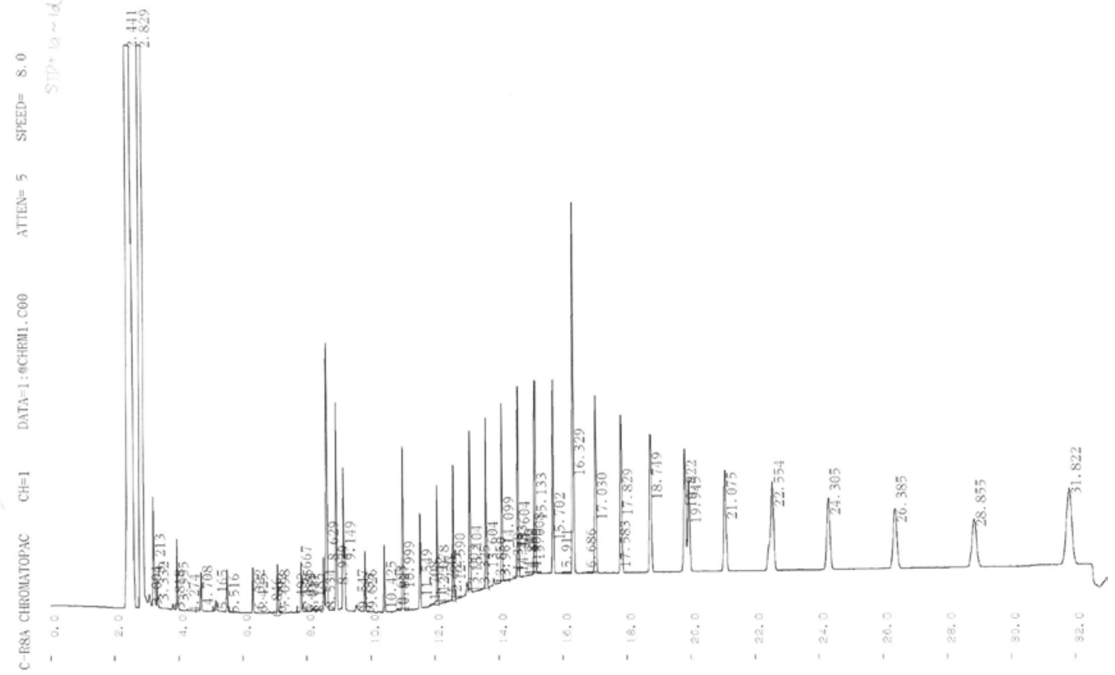

GC chart of a mixed solution of hydrocabon standards (C9 - C40) and four carbonates, **1a**-**1d**.

** CALCULATION REPORT **

CH	PKNO	TIME	AREA	HEIGHT	MK	IDNO	CONC	NAME
1	1	2.441	26859224	8218307			29.3974	
	2	2.829	63648556	25347168	V		69.6634	
	3	3.094	3671	810	V		0.004	
	4	3.213	16846	6376	V		0.0184	
	5	3.332	4039	1725	V		0.0044	
	6	3.818	899	344			0.001	
	7	3.955	8575	4081			0.0094	
	8	4.274	201	74			0.0002	
	9	4.708	4551	2201			0.005	
	10	5.165	1718	627			0.0019	
	11	5.516	4890	2406			0.0054	
	12	6.322	5059	2598			0.0055	
	14	6.846	247	98			0.0003	
	15	6.992	343	111	V		0.0004	
	16	7.098	5648	2795	V		0.0062	
	17	7.492	201	32			0.0002	
	19	7.835	5870	2895	V		0.0064	
	21	8.011	178	71	V		0.0002	
	22	8.185	335	93			0.0004	
	23	8.534	6332	3013			0.0069	
	24	8.629	43421	15306	V		0.0475	
	25	8.929	28430	11938	V		0.0311	
	26	9.149	27983	8221	V		0.0306	
	27	9.547	1719	362	S		0.0019	
	29	9.826	7453	3495			0.0082	
	30	10.425	8365	3858			0.0092	
	32	10.827	579	110	V		0.0006	
	33	10.999	21013	9441	V		0.023	
	34	11.549	12206	5467			0.0134	
	35	11.708	185	28	V		0.0002	
	36	11.942	791	143			0.0009	
	37	12.078	15989	6818	V		0.0175	
	38	12.25	252	45	V		0.0003	
	39	12.425	283	55			0.0003	
	40	12.59	18113	7736	V		0.0198	
	41	13.003	3358	785	V		0.0037	
	42	13.104	22859	9385	V		0.025	
	43	13.325	1233	129	V		0.0013	
	44	13.604	25484	9841	V		0.0279	
	45	13.85	2908	525	V		0.0032	
	46	13.967	852	182	V		0.0009	
	47	14.099	27446	10405	V		0.03	
	48	14.358	2574	193	V		0.0028	
	49	14.483	1326	175	V		0.0015	
	50	14.604	29046	11104	V		0.0318	
	51	14.717	598	135	V		0.0007	
	52	14.8	457	106	V		0.0005	
	53	14.9	607	89	V		0.0007	
	54	15.008	372	68	V		0.0004	
	55	15.133	30534	11128	V		0.0334	
	56	15.702	30870	11096			0.0338	
	57	15.911	122	38			0.0001	
	58	16.329	64025	21231			0.0701	
	60	17.03	32603	10173			0.0357	
	62	17.829	32181	9041			0.0352	
	63	18.749	31673	7934			0.0347	
	64	19.822	31611	7037			0.0346	
	65	19.945	23926	5397	V		0.0262	
	66	21.075	29731	5781			0.0325	
	67	22.554	38418	5130			0.042	
	68	24.305	28518	4112			0.0312	
	69	26.385	28093	3415			0.0307	
	70	28.855	27387	2745			0.03	
	71	31.822	53162	4376			0.0582	

References

1. Steiner, D.; Ivison, L.; Goralski, C. T.; Appell, R. B.; Gojkovic, J. R.; Singaram, B. *Tetrahedron: Asymmetry* **2002**, *13*, 2359–2363. doi:org/10.1016/S0957-4166(02)00646-8

2. Morikawa, H.; Minamoto, M.; Gorou, Y.; Yamaguchi, J.; Morinaga, H.; Motokucho, S. *Bull. Chem. Soc. Jpn*. **2018**, *91*, 92–94. doi:org/10.1246/bcsj.20170300 doi:org/10.1007/s00289-015-1546-6

3. Kováts, E. *Helv. Chim. Acta*. **1958**, *41*, 1915–1932. doi:org/10.1002/hlca.19580410703

4. Martínez, J.; Fernández-Baeza, J.; Sánchez-Barba, L. F.; Castro-Osma, J. A.; Lara-Sánchez, A.; Otero, A. *ChemSusChem* **2017**, *10*, 2886–2890. doi:org/10.1002/cssc.201700898

5. Fiorani, G.; Stuck, M.; Martín, C.; Belmonte, M. M.; Martin, E.; Escudero-Adán, E. C.; Kleij, A. W. *ChemSusChem* **2016**, *9*, 1304–1311. doi:org/10.1002/cssc.201600238

6. Plummer, C. M.; Kraft, P.; Froese, J.; Hudlický, T.; Rook, T. J.; Jones, O. A. H.; Hügel, H. M. *Asian J. Org. Chem*. **2015**, *4*, 1075–1084. doi:org/10.1002/ajoc.201500233

7. Blair, M.; Andrews, P. C.; Fraser, B. H.; Forsyth, C. M.; Junk, P. C.; Massi, M.; Tuck, K. L. *Synthesis* **2007**, 1523–1527. doi:10.1055/s-2007-966033

8. Leung, A. E.; Rubbiani, R.; Gasser, G.; Tuck, K. L. *Org. Biomol. Chem*. **2014**, *12*, 8239–8246. doi:10.1039/C4OB01662A
